# Structure–Activity Relationships of Silver(I)- and Gold(I)–NHC Complexes Reveal Distinctly Different Responses of Cisplatin-Resistant Ovarian Cancer to Bis-NHC–Gold(I) Derivatives

**DOI:** 10.1021/acs.jmedchem.5c02355

**Published:** 2026-01-27

**Authors:** Julia H. Bormio Nunes, Christina Hacker, Monika Caban, Daniel Valcanover, Patrick A. Yassemipour, Sebastian Türck, Ingo Ott, Lukas Skos, Andrea Bileck, Christopher Gerner, Samuel M. Meier-Menches, Thomas Mohr, Walter Berger, Christian R. Kowol, Petra Heffeter

**Affiliations:** † Institute of Inorganic Chemistry, Faculty of Chemistry, 27271 University of Vienna, Vienna 1090, Austria; ‡ Center for Cancer Research and Comprehensive Cancer Center, Medical University of Vienna, Vienna 1090, Austria; § Research Cluster “Translational Cancer Therapy Research”, Vienna 1090, Austria; ∥ Institute of Medicinal and Pharmaceutical Chemistry, 26527 Technische Universität Braunschweig, Braunschweig 38106, Germany; ⊥ Department of Analytical Chemistry, Faculty of Chemistry, University of Vienna, Vienna 1090, Austria; # Joint Metabolome Facility, Medical University of Vienna and University of Vienna, Vienna 1090, Austria

## Abstract

Ovarian cancer (OC) is the most lethal gynecological malignancy, with platinum resistance posing a major therapeutic challenge. To explore alternatives, we synthesized silver- and gold-based *N*-heterocyclic carbene (NHC) complexes differing only in their central metal ion and evaluated their activity in platinum-resistant OC. Structure–activity relationships revealed distinct metal-dependent behaviors. Silver complexes showed little variation with ligand modifications, whereas gold complexes displayed pronounced differences. Two bis-NHC–gold compounds were of particular interest: In an isogenic OC resistance model (A2780 and A2780/cis), [(NHC_2_)_2_Au]Br showed cross-resistance, while [(NHC_1_)_2_Au]Br induced collateral sensitivity. These effects were independent of intracellular accumulation, apoptosis induction, or TrxR inhibition. Instead, proteomic and metabolic analyses demonstrated that [(NHC_1_)_2_Au]Br inhibited oxidative phosphorylation, forcing a metabolic shift to aerobic glycolysis. As A2780/cis cells already rely on maximal glycolysis, [(NHC_1_)_2_Au]Br caused an energy collapse. These findings highlight a metabolic vulnerability in cisplatin-resistant OC that may be exploited for the development of novel therapeutic candidates.

## Graphical abstract

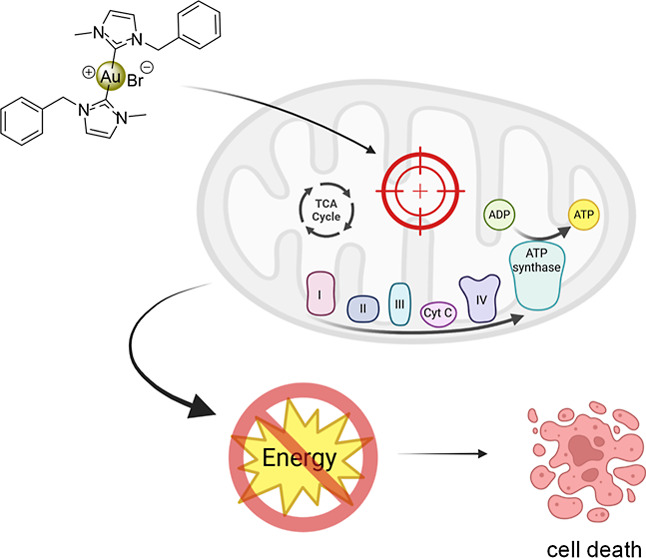

## Introduction

Cancer is the second leading cause of death worldwide and remains one of the main global health priorities.^1^ Ovarian cancer (OC) is the eighth most commonly diagnosed cancer among women and is generally detected at an advanced or late stage, when the survival rate is very low.^2^ As a result, OC continues to be the most lethal gynecological malignancy. First-line OC treatment typically involves surgery followed or/and preceded by chemotherapy, which combines a platinum- (Cisplatin or Carboplatin) and a taxane-based drug.^3^ However, recurrence of OC after initial platinum-based chemotherapy is common due to the development of platinum resistance, and treatment options in such cases are very limited.^2^ In recent years, treatment innovations such as bevacizumab, poly­(ADP-ribose) polymerase inhibitors (PARPi), and antibody drug conjugates (ADC) have emerged. Despite these advances, the 5 year survival rate for OC has not significantly improved,^3^ underscoring the urgent need for new chemotherapeutic agents to treat therapy-resistant OC.

Among metallodrugs, one nonplatinum-based compound that has been extensively studied as an anticancer agent across various cancer types is Auranofin.^4^ It is an orally administered gold compound approved for the treatment of arthritis. Notably, patients with rheumatoid arthritis who received this treatment were found to have an unexpectedly low risk to develop malignant diseases.^5^ The mechanism of action of Auranofin involves inhibition of thioredoxin reductase (TrxR) as well as induction of reactive oxygen species (ROS) and apoptotic cell death.^6,7^ Noteworthily, Auranofin is not affected by the same resistance mechanisms as Cisplatin or Carboplatin in epithelial OC.^8,9^ Consequently, the drug has been evaluated as an OC therapeutic in clinical trials (clinicaltrials.gov, NCT01747798, NCT03456700); however, so far, it has limited success. This could be associated with the insufficient stability of Auranofin under the physiological conditions. Consequently, many novel gold-based metallodrugs contain ligands that are bound strongly to the central gold atom. Among those, *N*-heterocyclic carbenes (NHCs) play a major role and the resulting gold–NHC complexes have been widely studied as anticancer drug candidates, many of them effectively inhibiting TrxR.^9–26^

Besides gold, silver has attracted considerable attention due to its diverse biological activities. Historically used in its metallic form as an antimicrobial agent with good tolerability, silver (nitrate) has also found medical applications in wound healing.^27^ Although the precise mechanism underlying silver’s activity remains unclear, its antibacterial effects involve the release of silver­(I) ions, which can penetrate cell membranes and disrupt cellular functions.^28^ Similarly to gold, silver complexes with NHC ligands have also been used to enhance stability,^29,30^ and such complexes have been explored as anticancer agents.^31–34^ For example, bis-NHC–silver­(I) compounds revealed multitargeted activity against OC cells,^35^ strong inhibition of TrxR, and significant antitumor activity in mice.^36^

However, although numerous silver and gold complexes bearing NHC ligands have been studied in various cancer cell lines, only a few studies have directly compared structurally identical silver and gold compounds,^15–17,20,21^ and none have specifically addressed their potential to break platinum resistance. Consequently, the goal of this work was to synthesize silver- and gold-NHC compounds (mono- and biscarbenes) using two different NHC ligands (NHC_1_ = 1-benzyl-3-methyl-imidazole-2-ylidene and NHC_2_ = 4,5-dichloro-1-benzyl-3-methyl-imidazole-2-ylidene) and to evaluate their effects with a special focus on drug-resistant OC. We discovered that despite their structural similarities, silver complexes differ distinctly in their anticancer activities from the gold analogues. Moreover, we identified [(NHC_1_)_2_Au]Br as a promising candidate for treating platinum-resistant OC, with cisplatin-resistant cells presenting collateral sensitivity. These effects were attributed to its specific antimetabolic activities, suggesting that certain gold-based derivatives could be particularly effective against cells that have undergone metabolic alterations during the development of resistance.

## Results and Discussion

### Synthesis and Characterization

As a starting point, a panel of mono-NHC–silver and –gold complexes with either 1-benzyl-3-methyl-imidazole-2-ylidene (NHC_1_) or 4,5-dichloro-1-benzyl-3-methyl-imidazole-2-ylidene (NHC_2_; for ligand synthesis, see Scheme S1) and a bromide ligand as the leaving group (Figure 1 and Scheme 1) was synthesized. In addition, the respective bis-NHC complexes were developed with bromide as counterion, resulting in a total of eight compounds. The chloro-NHC derivative (NHC_2_) was selected based on reports showing that strongly electronegative substituents can markedly affect biological properties.^11,26,32^

**Figure 1:**
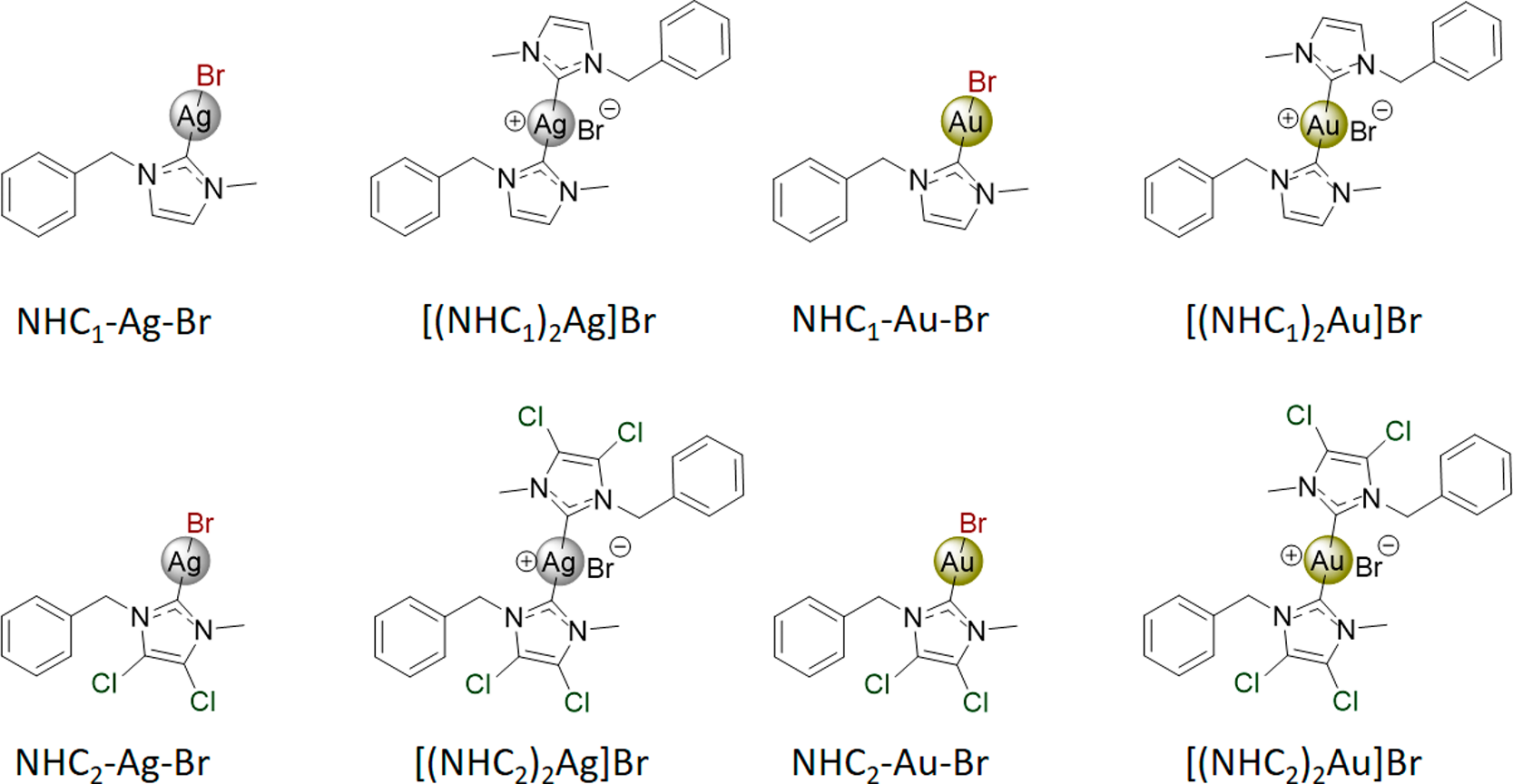
Synthesized silver­(I)- and gold­(I)–NHC complexes.

**Scheme 1:**
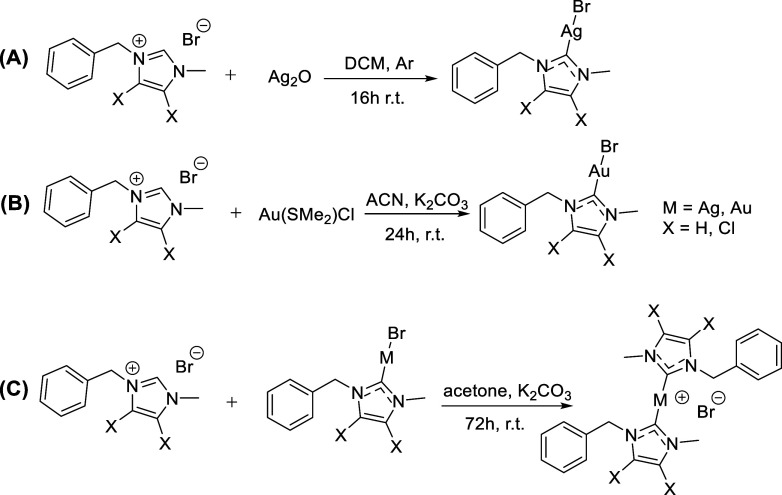
Synthesis Pathway for the Mono-NHC Complexes (A,B) and Bis-NHC Complexes (C)

The “free base route” was employed to obtain the mono-NHC–silver compounds, where a silver base (Ag_2_O) is used for the deprotonation of the imidazolium salt, also directly serving as the silver source for coordination.^15,35^ A very common route for obtaining gold–NHC compounds is by transmetalation of the silver–NHC complexes.^22,37^ However, if the reaction is not completed, then a mixture of silver–NHC with gold–NHC could be obtained. In order to avoid this mixture, a different route was employed for the synthesis of the mono-NHC–gold complexes, which involves the reaction of the imidazolium salt directly with the chloro­(dimethylsulfide)­gold­(I) precursor in the presence of potassium carbonate, called the “weak-base route”.^37,38^ Subsequently, for the synthesis of the bis-NHC complexes, an additional NHC ligand was coordinated to the mono-NHC complexes in the presence of potassium carbonate (Scheme 1).^37,38^

All compounds were analyzed by elemental analysis (±0.4%) as well as ^1^H and ^13^C nuclear magnetic resonance (NMR) spectroscopy. The disappearance of the signals at ∼9.3–9.5 ppm in the ^1^H NMR spectra of the complexes confirms the deprotonation of the imidazole salts. It is important to note that the NHC abbreviation in the formulas (either NHC_1_ or NHC_2_) represents the deprotonated ylidene ligand. In line with the literature,^39,40^ the shift in the ^13^C NMR spectra of the carbene carbon (N–C–N) in the imidazole ring from ∼135 ppm to ∼180 ppm indicated coordination of the carbene to silver­(I) and gold­(I), respectively (Figures S1–S9). For the mono- and bis-NHC–silver complexes, an equilibrium between both species occurred in solution, with the rate depending on the solvent.^41^ If this dynamic equilibrium is slow, single peaks for each carbon can be observed in the ^13^C NMR spectrum.^36,39,42,43^ For our complexes, single peaks were observed in the ^13^C NMR spectrum for the mono- and bis-NHC–silver compounds, suggesting a fast equilibrium in DMSO between both species. Although using the soft electron spray ionization mass spectrometry (ESI-MS), for all silver complexes, the free ligand could also be detected. For the mono-NHC–silver complexes, the 1:1 complex was not observed; only the [M­(NHC)_2_]^+^ species was found, consistent with the presence of the bis-species in solution. Naturally, this ion species was also observed for the bis-NHC–silver derivatives (examples in Figure S10). For the bis-NHC–gold complexes, the [Au­(NHC)_2_]^+^ ion was identified (Figure S11). Interestingly, for both mono-NHC–gold complexes, the [Au­(NHC)_2_]^+^, [(NHC)­Au­(ACN)]^+^ species, and [(NHC)­Au­(ACN)­(MeO^–^)+H]^+^ species were detected (Figure S12). The last could also be confirmed by high-resolution mass spectrometry (HRMS) for NHC_2_–Au–Br (Figure S12C). These mass spectrometry data already indicate distinct differences in the stability of the silver and gold complexes.

### Stability Studies

As the NHC complexes are not soluble in pure aqueous media, their stabilities were first evaluated by NMR spectroscopy in deuterated dimethyl sulfoxide (DMSO-*d*_6_), revealing no changes for at least 48 h (Figures S13 and S14). It was noticed that [(NHC_2_)_2_Ag]Br has a very low solubility, even in the presence of DMSO, and was therefore excluded from further experiments. Next, the stability of the complexes (100 μM) in 30% DMSO in phosphate buffer (PB) at pH 7.4 and 37 °C for 24 h was evaluated using UV–vis spectroscopy. Most of the complexes precipitated in solution over the 24 h period, as indicated by a gradual decrease in absorbance across the entire spectrum. For mono-NHC–silver complexes, we observed a slight change in the spectra between 0 and 10 min, most likely due to hydrolysis. After this period, only precipitation was observed (for NHC_1_–Ag–Br, see Figure S15). This phenomenon was also reported for other silver–NHC complexes in the literature.^43,44^ For the mono-NHC–gold compounds, changes in the spectra were observed within the first few hours with only slight precipitation over time (Figure S16A,B). According to the literature, these changes are most likely due to a ligand exchange process, resulting in the formation of solvent adducts as well as the bis-NHC complex.^12,40^ For the bis-NHC–gold complexes, no differences were seen within 24 h (Figure S16C,D), which was also confirmed by HPLC-MS (Figure S17), indicating high stability.

### TrxR Inhibition

As a first biological evaluation, the TrxR inhibition properties of seven silver– and gold–NHC complexes were investigated in a cell-free assay. As already mentioned, [(NHC_2_)_2_Ag]Br was excluded due to its very low solubility. All compounds were active against the human cytosolic TrxR1 in the low μM to nM range, except for [(NHC_1_)_2_Au]­Br, which was the least active compound (IC_50_ ∼12 μM) (Figure 2 and Table 1). Regarding the structure–activity relationships, the silver compounds were more active than the respective gold analogues. Moreover, compounds with two NHC ligands were less active than compounds with only one NHC ligand. Thus, the most active complex was NHC_2_–Ag–Br at an IC_50_ of 0.039 μM. Overall, these results are consistent with previous literature reports indicating that bis-NHC–gold compounds are more inert compared to mono-NHC–gold compounds, which explains their generally lower TrxR inhibition potential.^11,19,40^

**Figure 2:**
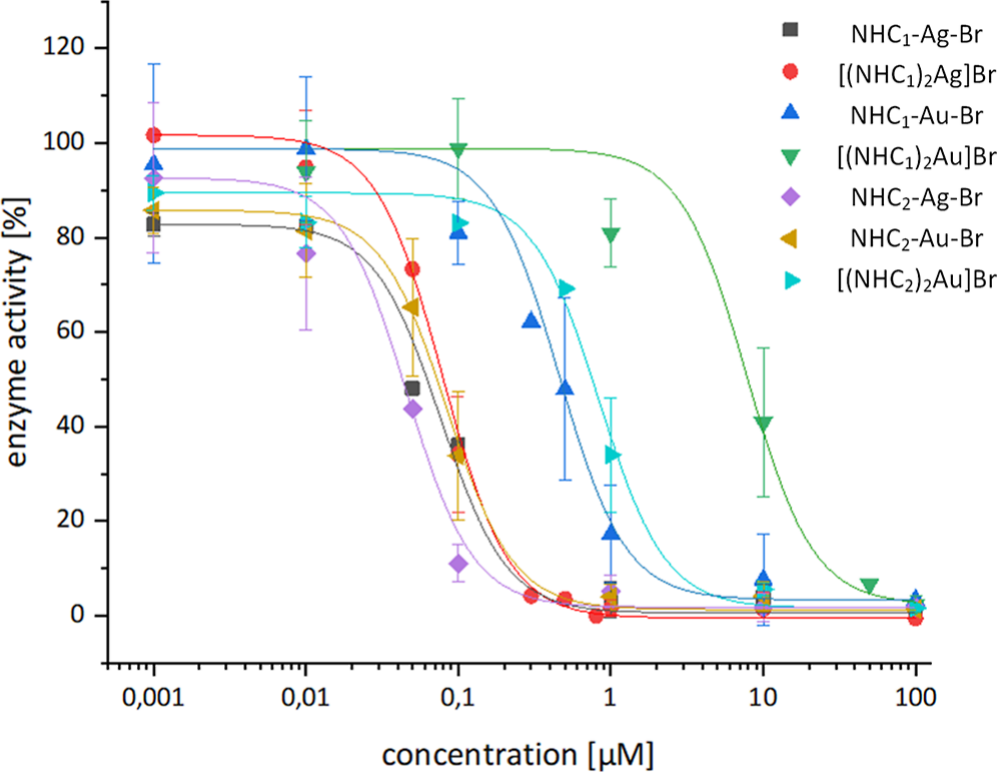
TrxR1 (cytosolic form) activity (cell-free) after incubation with the silver– and gold–NHC compounds.

**Table 1:** TrxR1 Inhibition and Antiproliferative Activity in A2780, A2780/Cis, and Hs545SK Cells after 72 h

compound	TrxR1 inhibition (μM)^a^	A2780 (IC_50_, μM)^b^	A2780/cis (IC_50_, μM)^b^	relative resistance^c^	Hs545SK (IC_50_, μM)^b^	selectivity index^d^
Cisplatin	n.d.	1.98 ± 0.26	8.05 ± 0.52	4.08**	>50	>25
Auranofin	n.d.	0.55 ± 0.06	2.68 ± 0.20	4.89**	3.47 ± 0.28	6.3
AgNO_3_	n.d.	1.91 ± 0.15	2.94 ± 0.04	1.54*	15.2 ± 0.3	7.9
NHC_1_–Ag–Br	0.052 ± 0.004	0.95 ± 0.22	0.83 ± 0.09	0.87^ns^	22.7 ± 0.4	23.9
NHC_2_–Ag–Br	0.039 ± 0.005	0.58 ± 0.25	0.62 ± 0.15	1.14^ns^	13.5 ± 1.7	23.3
[(NHC_1_)_2_Ag]Br	0.086 ± 0.003	0.90 ± 0.13	0.84 ± 0.10	0.89^ns^	16.8 ± 1.6	18.7
NHC_1_–Au–Br	0.464 ± 0.162	9.32 ± 1.25	9.29 ± 1.50	0.99^ns^	11.5 ± 1.2	1.2
NHC_2_–Au–Br	0.046 ± 0.008	6.09 ± 0.75	7.55 ± 0.84	1.26^ns^	7.74 ± 0.50	1.3
[(NHC_1_)_2_Au]Br	11.89 ± 0.44	0.88 ± 0.09	0.46 ± 0.11	0.55*	30.7 ± 1.4	34.9
[(NHC_2_)_2_Au]Br	0.831 ± 0.006	1.47 ± 0.08	1.82 ± 0.18	1.23*	4.14 ± 0.50	2.8
NHC_1_ (imidazolium salt)	n.d.	>50	>50	n.d.	n.d.	n.d.
NHC_2_ (imidazolium salt)	n.d.	>50	>50	n.d.	n.d.	n.d.

a IC_50_ values correspond to the compound concentration that inhibits 50% of enzyme activity. Values are given as mean ± SD of three independent experiments.

b IC_50_ values were calculated from concentration–response curves. Values are the mean ± SD of three independent experiments performed in triplicate.

c Differences in sensitivity calculated by the quotient of the IC_50_ values of the resistant subline by the parental line. **p ≤ 0.01, *p ≤ 0.05; ns (not significantly different), calculated by one-sample *t*-test. n.d.: not determined.

d Selectivity index calculated by the ratio IC_50_(Hs545SK)/IC_50_(A2780).

Interestingly, [(NHC_2_)_2_Au]­Br was 14-fold more active than [(NHC_1_)_2_Au]­Br. Based on the hypothesis that [(NHC_2_)_2_Au]­Br, due to its electron-withdrawing chloro substituents, is less stable than [(NHC_1_)_2_Au]­Br, and knowing that gold compounds tend to interact with thiol groups of proteins, an incubation experiment using 5 equiv of cysteine in PB was performed for both bis-NHC–gold complexes and analyzed by HPLC-MS. The experiment confirmed that [(NHC_2_)_2_Au]Br interacts with cysteine, as indicated by the presence of the free ligand and a gold–cysteine adduct, whereas no such interaction was observed for [(NHC_1_)_2_Au]Br (Figure S18). Also, incubation in the RPMI cell culture medium revealed that minor peaks were formed for [(NHC_2_)_2_Au]Br within 24 h, corresponding to the free ligand and the gold–cysteine adduct. In contrast, [(NHC_1_)_2_Au]Br was completely stable under these conditions (Figure S19).

### Activity against Chemosensitive and Platinum-Resistant OC and Nontumorigenic Cells

Continuing the investigation on how the structural differences and the observed complex stabilities impact the biological activities of the new drugs, the in vitro anticancer effect was assessed using A2780 OC cells. At the cell culture conditions and concentrations used, all compounds were soluble and did not precipitate. As shown in Figures 3 and S20 and Table 1, all compounds were active against the cancer cells in the low μM range (between 0.5 and 10 μM), comparable to Auranofin. When comparing silver and gold analogues, the mono-NHC–silver complexes were approximately 10-fold more active than the mono-NHC–gold compounds. Furthermore, the monocomplexes exhibited consistent activities regardless of the NHC ligand. However, for the bis-NHC–gold compounds, the presence of NHC_1_ resulted in enhanced activity compared to NHC_2_. The bis-NHC–silver compound had a comparable activity to that of its respective bis-NHC–gold analogue. Noteworthily, the metal-free imidazolium salts of NHC_1_ and NHC_2_ alone were not active up to concentrations of 50 μM (Figure S21). Interestingly, overall, the anticancer activity of the compounds only partly aligned with their TrxR1 inhibition potential (especially in the case of the bis-NHC–gold complexes).

**Figure 3:**
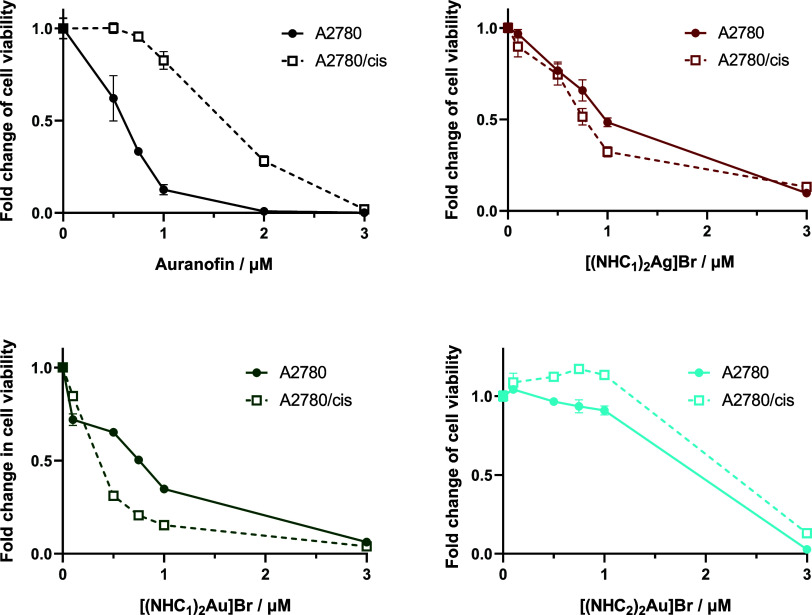
Resistance of the A2780/cis cells against Auranofin and the bis-NHC complexes. Cell viability was evaluated in A2780 vs A2780/cis cells by the MTT assay after 72 h of drug incubation. Values are given as mean ± standard deviation (SD) calculated from triplicates of one representative experiment out of three.

To confirm that the observed activity of the silver compounds in cell culture is due to the silver metal complex (i.e., resulting from the Ag–C bond rather than from the activities of isolated ligands and/or silver ions), we tested AgNO_3_ in combination with both NHC ligands (imidazolium salts) (Figure S22). As free NHC ligands are unstable under normal biological conditions, the imidazolium salts were used. Indeed, silver ions (alone as well as in combination with the imidazolium salts, i.e., the NHC precursors) were cytotoxic to A2780 cells but only at 2- to 4-fold higher concentrations compared to the synthesized metal complexes. This suggests that the activities of the new silver complexes cannot be solely attributed to the released silver (especially in the case of NHC_2_–Ag–Br).^44–46^ This data is in good agreement with a recent study by Esarev et al., which indicated that silver–NHC complexes have a lower tendency to release silver ions in solution with high chloride levels (i.e., phosphate-buffered saline, PBS) and that these silver complexes remain intact.^36^ Consequently, at least under the conditions used in our experiments, the intact Ag–NHC moiety seems to play an important role in anticancer activity.

To test the impact of platinum resistance on the compound panel, the activity against the cisplatin-resistant A2780/cis subclone was also tested (Figures 3 and S20 and Table 1). These cells were not only resistant to their selection drug, Cisplatin, but they also displayed significant cross-resistance against both Auranofin (in agreement with the literature^47^) as well as AgNO_3_. Also, for the gold compounds with the NHC_2_ ligand, a trend toward cross-resistance was observed; however, it only reached statistical significance for [(NHC_2_)_2_Au]­Br. In contrast, all silver compounds as well as NHC_1_–Au–Br were equally effective in parental and cisplatin-resistant cells, not being affected by platinum resistance. Strikingly, A2780/cis cells displayed a distinct collateral sensitivity against [(NHC_1_)_2_Au]­Br. Although other biscarbene gold­(I) compounds have been reported to be effective in drug-resistant cells,^47,48^ collateral sensitivity is rarely described. For example, collateral sensitivity was also observed for A2780/R cells against other mono- and bis-NHC–gold compounds^49^ (although the underlying mechanisms were not further investigated) and for A2780CP70 cells against a gold­(III) compound.^50^ To test whether the collateral sensitivity of the A2780/cis cells to [(NHC_1_)_2_Au]Br is related to their Cisplatin resistance, a revertant clone was generated, which regained sensitivity to Cisplatin after 1 month without selection. Indeed, the regained sensitivity also abolished collateral sensitivity to [(NHC_1_)_2_Au]­Br, suggesting a mechanistic connection (Figure S23).

Finally, the selectivity toward cancer cells was evaluated by comparing the cytotoxicity in the A2780 cell model with nontumorigenic Hs545SK fibroblasts. Interestingly, big differences were observed: the silver complexes were selective to cancer cells with selectivity indexes (SI) between 19 and 24. Also, both bis-NHC–gold compounds, especially [(NHC_1_)_2_Au]­Br, were selective for cancer cells. In contrast, all mono-NHC–gold compounds had no significant cancer cell selectivity (SI ∼1.3).

### Drug Uptake in A2780 vs A2780/Cis Cells

Next, it was investigated whether the differences in cell viability between A2780 and A2780/cis cells were attributable to drug uptake. To this end, intracellular levels of silver and gold were measured after 5 h of incubation by inductively coupled plasma mass spectrometry (ICP–MS, Figure 4A). This method allows the quantification of the total silver and gold content inside the cells. In case of the silver drugs, the uptake of mono- and bis-NHC complexes was similar and 30–50% lower levels were observed in the resistant subclone compared to the parental line. Treatment with the reference AgNO_3_ resulted in higher silver levels than treatment with [(NHC_1_)_2_Ag]Br in both A2780 and A2780/cis cells (Figure 4B). These effects are interesting as there was no correlation in the sensitivity of the cells with drug uptake, which might indicate that a large fraction of the intracellular silver is actually not involved in the mode of action.

**Figure 4:**
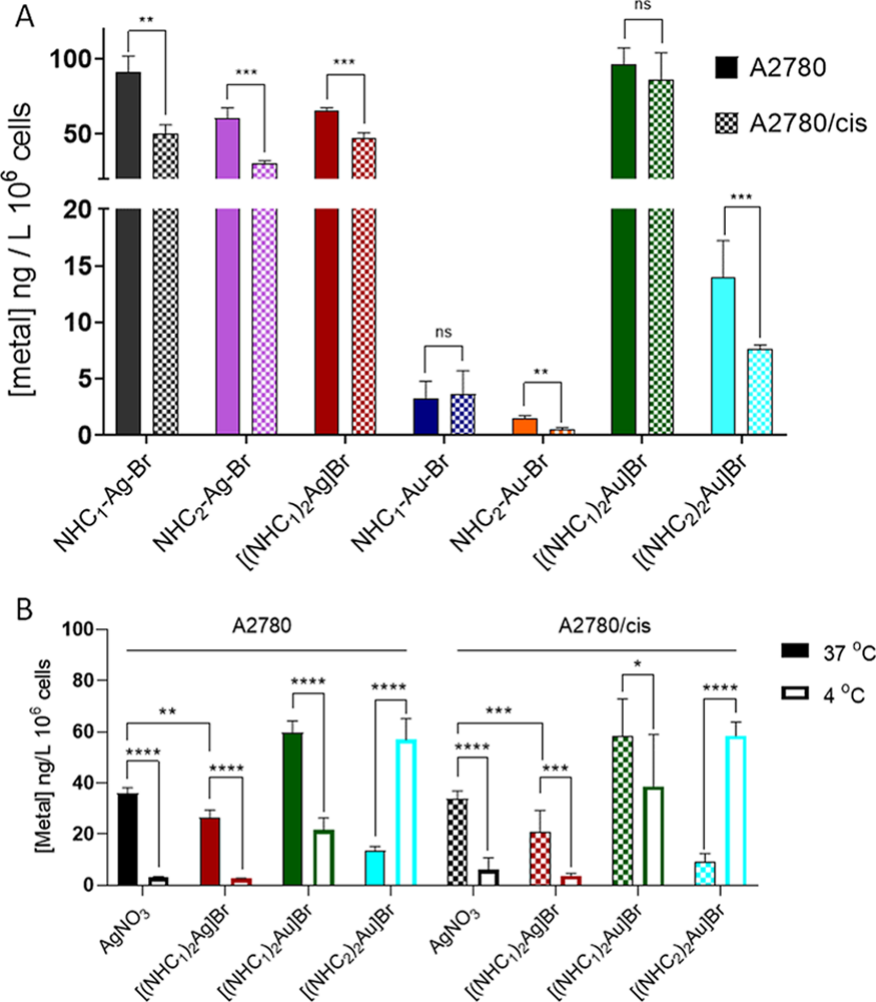
Intracellular silver and gold levels after treatment with the indicated drugs at 5 μM (A) in A2780 and A2780/cis cells after 5 h of incubation at 37 °C and (B) after 5 h of incubation at 4 °C vs 37 °C. Cells were digested and measured by ICP–MS. Results were normalized to the cell number, and values are given as mean ± SD of two or three independent experiments. Statistical significance was calculated using a paired-*t* test (*p < 0.05; **p < 0.01; ***p < 0.001; ****p < 0.0001; ns: not significant).

In contrast, there were distinct differences in the case of the gold drugs depending on the number of NHC-ligands. In parental cells, only [(NHC_1_)_2_Au]Br displayed drug uptake comparable to the silver complexes. Already in the case of [(NHC_2_)_2_Au]­Br, an about 1.9-fold reduced uptake was observed. This further dropped to <5% uptake of NHC_1_–Au–Br and NHC_2_–Au–Br in comparison to the respective silver complexes. Noteworthily, in contrast to the silver panel, a correlation between anticancer activity and intracellular drug levels was clearly visible for the gold compounds. The positively charged bis-gold complexes were taken up much more efficiently than the more neutral monogold complexes. Noteworthily, there was no difference in the gold levels of [(NHC_1_)_2_Au]Br between A2780 and A2780/cis cells, which indicates that the collateral sensitivity is not based on enhanced intracellular gold levels in the resistant subclone. For the mono-NHC–gold compounds, differences in the gold levels between A2780 and A2780/cis cells were dependent on the NHC ligand. While for the complex with NHC_1_, no difference in the gold levels was observed, the complex with the NHC_2_ ligand was characterized by a lower gold accumulation in the resistant line.

To better understand the molecular transport mechanisms involved, additional experiments comparing the uptake at 37 and 4 °C were performed for selected compounds, AgNO_3_, [(NHC_1_)_2_Ag]­Br, [(NHC_1_)_2_Au]­Br, and [(NHC_2_)_2_Au]­Br, with the hypothesis that active energy-dependent transport proteins should not be functional at lower temperatures. Indeed, the cellular metal levels of all compounds (with the exception of [(NHC_2_)_2_Au]­Br) were distinctly reduced at 4 °C compared to 37 °C in both cell lines (Figure 4B), suggesting active drug uptake. In the case of [(NHC_2_)_2_Au]­Br, a rather unusual effect occurred, as the intracellular gold accumulation was ∼4-fold and 6-fold (for A2780 and A2780/cis, respectively) higher at 4 °C than at 37 °C (Figure 4B). Interestingly, there is also one report on a gold­(III) compound, [Au­(py^b^-H)­(PTA)­Cl]­PF_6_, where a similar effect was described.^51^ Comparable to this study, also for us, one possible explanation for these unexpected results could be that [(NHC_2_)_2_Au]Br is subject to an active energy-dependent efflux mechanism in A2780 cells, which is inhibited under low-temperature conditions. Consequently, it could be speculated that the low gold levels after treatment with [(NHC_2_)_2_Au]Br (and possibly NHC_1_–Au–Br and NHC_2_–Au–Br) are based on a similar efflux mechanism that protects the cells. Noteworthily, there are literature reports that A2780 cells have (high) expression of the copper efflux transporters ATP7A/B, which are even further increased in the cisplatin-resistant subline.^52^ Consequently, we performed an uptake experiment with [(NHC_2_)_2_Au]Br in competition with the ATP7A/B substrate CuCl_2_. As shown in Figure S24, the addition of CuCl_2_ was able to dramatically increase the intracellular levels of [(NHC_2_)_2_Au]Br in a concentration-dependent manner, especially in the sensitive cell model. In the resistant subclone, the effect was less pronounced, which can be explained by the higher ATP7A/B levels, which give the cells enough capacity to simultaneously efflux both the copper ions as well as the gold drug. Of note, a similar behavior was also observed with [Au­(py^b^-H)­(PTA)­Cl)]­PF_6_,^51^ indicating that, like for this gold­(III) compound, ATP7A/B could also be responsible for the low intracellular levels of [(NHC_2_)_2_Au]­Br.

### Apoptotic Cell Death and Mitochondrial Membrane Potential

Since the data indicated distinct differences in the anticancer activities and intracellular drug accumulation across the compound panel, it was of interest whether these differences might be linked to distinct modes of action. Consequently, as a next step, efforts were made to further characterize the cell death induction potential of the compound panel. Thus, annexin-V/propidium iodide (PI) as well as 5,5′,6,6′-tetrachloro-1,1′,3,3′-tetraethylbenzimi-dazolylcarbocyanine iodide (JC-1) staining were employed. In the annexin-V/PI staining, which allows discrimination between necrosis (PI-positive, annexin-V-negative) and different stages of apoptotic cell death (early stage: annexin-positive only; late-stage PI- and annexin-positive), no induction of relevant amounts of necrotic cell death was observed for any of the tested drugs (Figure 5A). Moreover, in most cases, the apoptosis-inducing potential directly correlated with the drug accumulation observed above. Consequently, at the tested concentration of 5 μM after 24 h, all silver complexes induced ∼40–50% cell death (Figure 5A). Significant apoptosis induction was also seen for [(NHC_1_)_2_Au]Br (∼30%), while the two mono-NHC–gold drugs were widely inactive. Noteworthily, despite its lower intracellular drug levels, [(NHC_2_)_2_Au]Br was the drug with the most efficient cell death induction (∼85% in A2780 cells). In line with the drug resistance, this effect was distinctly reduced in A2780/cis cells (Figure 5A). Interestingly, despite their collateral sensitivity, A2780/cis cells did not display enhanced apoptosis levels after [(NHC_1_)_2_Au]Br treatment, at least not during the first 24 h.

**Figure 5:**
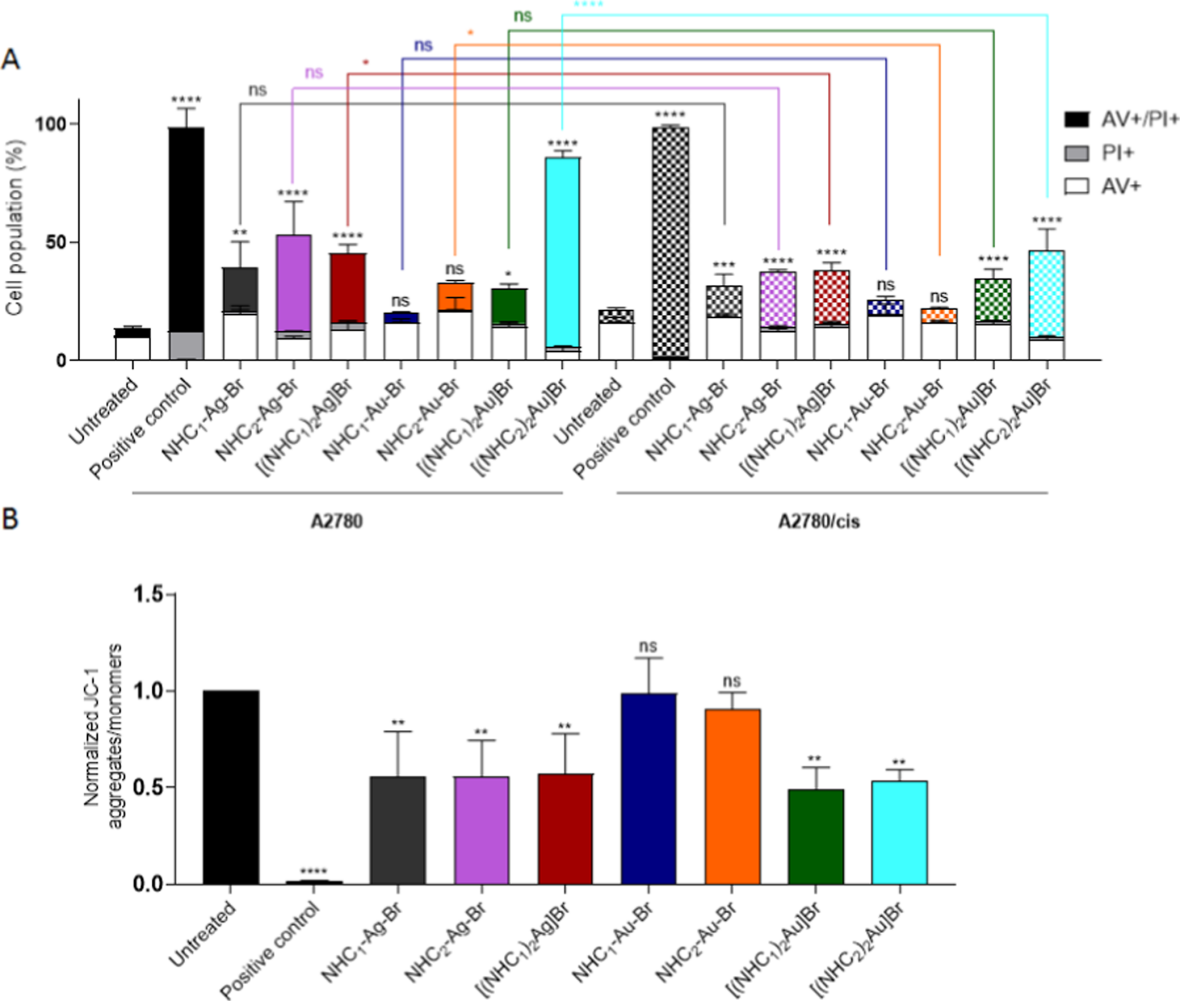
(A) Cell death evaluated by annexin-V/PI staining and (B) mitochondrial membrane potential evaluated by JC-1 staining (in A2780 cells). Cells were measured by flow cytometry after 24 h incubation with 5 μM of the drugs. Positive controls are cells heated up to 60 °C for 20–30 min. Results were normalized to untreated cells within the same cell line. Values are given as mean ± SD of three independent experiments. Statistical significance to untreated cells was calculated using two-way ANOVA and Dunnett’s multiple comparison test (*p < 0.05; **p < 0.01; ***p < 0.001; ****p < 0.0001; ns: not significant).

The JC-1 stain was used to investigate the effect of the drugs on mitochondria. This is important, as the mitochondrial membrane potential (MMP) plays a crucial role in regulating cell death and serves as an indicator of mitochondrial health. There are already literature reports that gold compounds can target mitochondria.^23,24^ Indeed, as Figure 5B shows (with the exception of the two mono-NHC–gold compounds, which are limited in their intracellular accumulation), all drugs reduced the MMP to a similar extent, indicating that depolarization of the mitochondria is not responsible for the observed differences in the gold compound panel.

Summarizing, intriguing differences between the two bis-NHC–gold derivatives were noted, despite their structural similarities, which prompted us to continue the investigation with a focus on these two compounds.

### Proteomics

To get more information about the mechanisms underlying the activities of the two bis-NHC–gold compounds as well as the reasons underlying the collateral sensitivity of the A2780/cis cells to [(NHC_1_)_2_Au]­Br, proteomic analyses were performed. Both cell clones were treated with 1.0 μM [(NHC_1_)_2_Au]Br or [(NHC_2_)_2_Au]Br or solvent for 16 h, and protein fractions from whole-cell lysates were collected in six biological replicates per condition. These concentrations and time points were selected based on preliminary assessment by time-lapse microscopy (data not shown) and viability assays after 24 h (Figure S25). The whole-cell lysates were proteolytically digested, and the peptide mixtures were analyzed by a data-dependent analysis strategy based on label-free quantification (LFQ) proteomics. The instrumentation included a nanoflow liquid chromatography-tandem mass spectrometry (nLC-MS/MS) system coupled to a TimsTOF Pro mass spectrometer operated in parallel accumulation-serial fragmentation (PASEF) mode. A total of 4622 proteins were identified in this data set. A principal component analysis revealed appropriate data homogeneity and clear separation of the individual cell lines and treatments (Figure 6A). A total of 3392 (73%) significantly regulated proteins were observed between untreated A2780/cis and A2780 cells, supporting previous studies that have already shown that resistance in this cell line resulted in large changes in whole-cell^53^ and mitochondrial^54^ proteomes. With our approach, the resistant clone showed upregulated gene ontology sets of biological processes (GOBP) corresponding to rRNA processing and translation, together with upregulated metabolic processes, including glutamate, pentose-phosphate, and fructose 6-phosphate (Table S1). Moreover, the resistant clone featured a down-regulation of proteins belonging to oxidative phosphorylation (OXPHOS), ROS, and focal adhesion compared to the sensitive clone. Consequently, resistance in A2780 cancer cells is associated with an increased reductive and metabolic capacity, which comes at the cost of mitochondrial processes, including ATP generation. This might increase the susceptibility of A2780/cis cells against mitochondrial interference.

**Figure 6:**
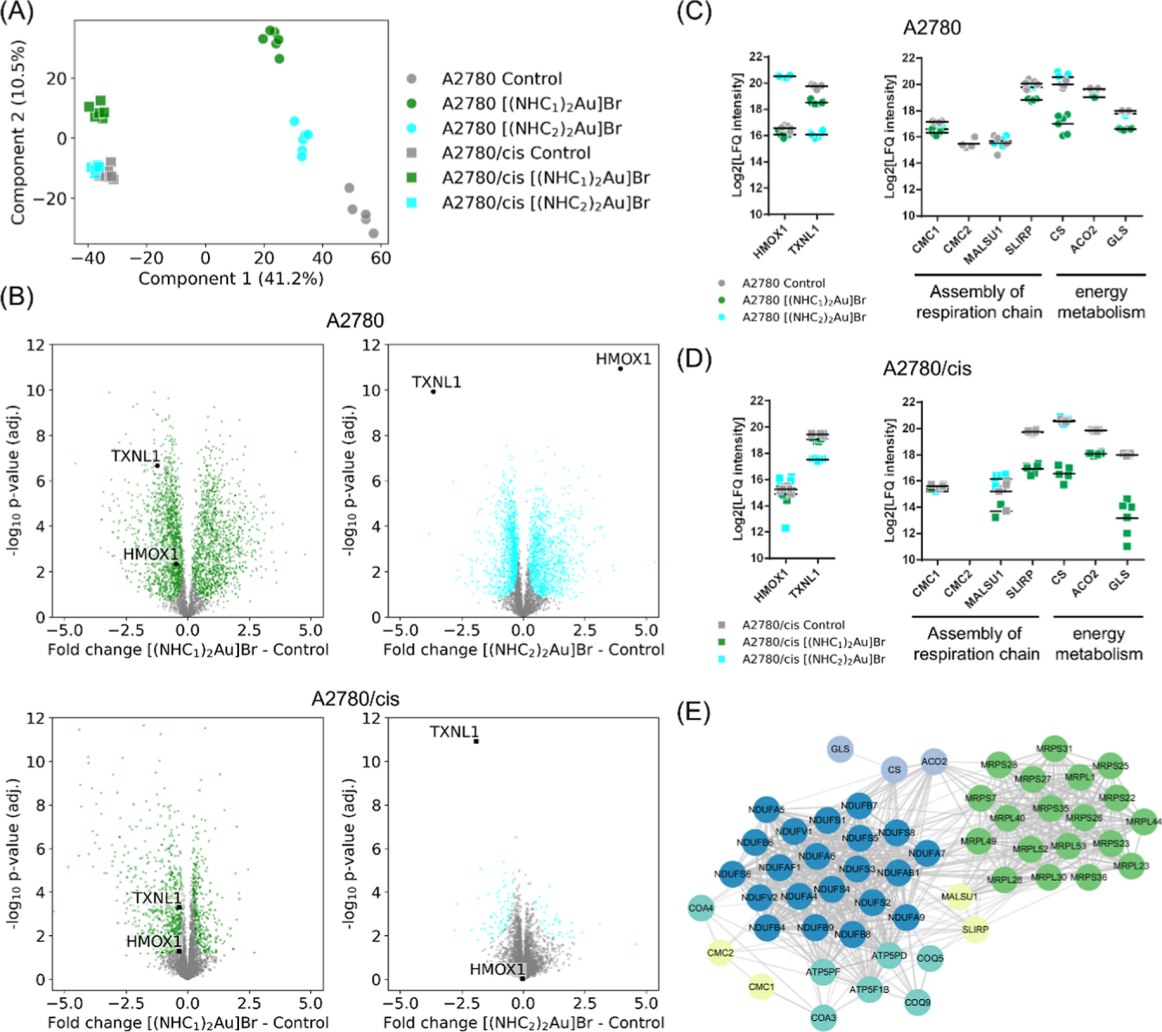
(A) Principal component analysis of proteome profiles of whole cell lysates of untreated (control) A2780 and A2780/cis cells and treated either with [(NHC_1_)_2_Au]Br or [(NHC_2_)_2_Au]Br at 1 μM for 16 h. (B) Volcano plots of sensitive A2780 and cisplatin-resistant A2780/cis cells treated with [(NHC_1_)_2_Au]Br and [(NHC_2_)_2_Au]­Br. (C) Round dot plots show the regulation of HMOX1, TXNL1 as characteristic treatment effects in A2780 cancer cells, as well as specific mitochondrial proteins related to the assembly of the respiratory chain and energy metabolism. Same coloring as in (A). (D) Plots showing a weaker effect of treatment on HMOX1 and TXNL1 in A2780/cis cancer cells and the same mitochondrial proteins related to the assembly of the respiratory chain and energy metabolism. Same coloring as in (A). (E) Protein network of significantly down-regulated proteins upon treatment with [(NHC_1_)_2_Au]­Br, highlighting its specificity toward impairing mitochondrial function.

With respect to the compound treatments, a pronounced perturbation was observed in parental A2780 cells for [(NHC_1_)_2_Au]Br and [(NHC_2_)_2_Au]Br (Figure 6B), for which 2849 and 2097 significantly regulated proteins were observed, respectively (Table S2). A2780/cis cells treated with [(NHC_1_)_2_Au]Br and [(NHC_2_)_2_Au]Br showed 983 and 107 significantly regulated proteins, respectively (Figure 6B). While the perturbation of A2780/cis cells by [(NHC_1_)_2_Au]Br was still considerable, there were only a few comparable effects upon treatment with [(NHC_2_)_2_Au]­Br. This implies that the resistant clone generally has a higher capacity to cope with the compound treatment and that the cellular adaptation to [(NHC_1_)_2_Au]Br treatment in the resistant clone was much stronger compared to [(NHC_2_)_2_Au]­Br.

A number of previous studies with gold­(I/III) derivatives found induction of heme oxygenase 1 (HMOX1) and down-regulation of thioredoxin-like 1 (TXNL1) as a characteristic signature of the treatment.^55–57^ This indicates a cytoprotective response of the cells against gold treatment. HMOX1 is part of the NRF2 pathway that protects cells from oxidative stress. More recently, TXNL1 was described to be degraded and not down-regulated by a gold­(III) cyclometalated compound.^58^ TXNL1 was reported to function as a redox-active chaperone.^59^ In our study, this effect was also observed for [(NHC_2_)_2_Au]Br treatment in the parental A2780 cells, whereas only TXNL1 down-regulation remained in the A2780/cis cells, underscoring the increased metabolic/reductive competence of the resistant clone. Interestingly, this signature was completely absent for [(NHC_1_)_2_Au]Br despite the structural similarity to [(NHC_2_)_2_Au]Br (Figure 6B,C). Despite this redox signature, the compounds did not considerably affect cytoplasmic heat shock or NRF2-mediated detoxification pathways (Figure S27).

To better understand the observed proteome perturbations, the top 15 changed GOBP term sets of the individual treatments were generated. GOBP terms represent molecular programs that an organism tries to achieve and span various levels of biological organization.^60^ They are therefore useful to categorize global cellular adaptation to drug treatment based on the set of significantly regulated proteins. More than half of the top GOBP terms in A2780 cells treated with [(NHC_1_)_2_Au]Br were linked to RNA processing, RNA metabolism, and ribosome biogenesis (Figure S26A). The [(NHC_2_)_2_Au]Br treatment shared similar top GOBP terms, indicating a similar perturbation (Figure S26B). However, the most interesting differences were observed in A2780/cis cells treated with the bis-NHC–gold compounds. While for [(NHC_2_)_2_Au]­Br, there were no significant GOBP terms enriched, probably due to the limited number of significantly regulated proteins, [(NHC_1_)_2_Au]­Br-treated cells presented top GOBP terms related to metabolism, energy, and cellular respiration (Figure S26C,D), which were key features of the resistance signature.

The effects of [(NHC_1_)_2_Au]Br on mitochondrial proteins, related to energy metabolism and OXPHOS, were of particular interest. [(NHC_1_)_2_Au]Br down-regulated mitochondrial ribosomal proteins in both parental and resistant clones (Figure S28). Moreover, proteins of NADH dehydrogenase (complex I) of the OXPHOS cascade were down-regulated by [(NHC_1_)_2_Au]Br also in both clones, while complex III and V were not affected in a comparable manner (Figure S29). Especially in the resistant clones, several complex I proteins were quantitatively down-regulated so that they were not detectable anymore. Additionally, proteins responsible for the assembly of the mitochondrial respiration chain were also downregulated by [(NHC_1_)_2_Au]­Br, including COX assembly mitochondrial protein 1/2 homologues (CMC1/2), mitochondrial assembly of ribosomal large subunit protein 1 (MALSU1) and mitochondrial SRA stem-loop-interacting RNA-binding protein (SLIRP) but not necessarily by [(NHC_2_)_2_Au]Br (Figure 6D). Those were accompanied by down-regulation of the citrate cycle-related proteins citrate synthase (CS), mitochondrial aconitate hydratase (ACO2), and mitochondrial glutaminase (GLS). In summary, [(NHC_1_)_2_Au]Br seems to perturb A2780 and A2780/cis cells by specifically targeting their essential mitochondrial functions, based on OXPHOS and metabolism, including mitochondrial translation (Figure 6D). This could be responsible for the exceptional collateral sensitivity of A2780/cis cells toward the treatment with [(NHC_1_)_2_Au]­Br.

With respect to other gold drugs, recently, a report on A2780 (parental) cells compared the effects of a mono- and its respective bis-NHC–gold compound (the ligand differs from NHC_1_ with a butyl substituent instead of a benzyl). Treatment with these gold­(I) compounds showed proteins involved in gene expression regulation, nucleoside metabolic processes, and the influence of the bis-NHC–gold compound on energy metabolism. However, the evaluation was done only in parental cells and revealed different enriched GOBP terms compared to those observed with our compounds in the same cell line. Interestingly, both compounds were potent TrxR inhibitors, which was not observed for [(NHC_1_)_2_Au]­Br.^25,61^

### Cell Metabolism

Based on the proteomics results for [(NHC_1_)_2_Au]­Br-treated A2780/cis cells, the impact of [(NHC_1_)_2_Au]Br on cellular metabolism was investigated. In more detail, real-time Seahorse measurements of the cell metabolism using the Mito Stress Test were performed. As is obvious from Figure 7A, the oxygen consumption rate (OCR), indicative of mitochondrial respiration in untreated A2780/cis, was lower when compared to A2780 cells. This is particularly evident at maximal respiration, following carbonyl cyanide-*p*-trifluoromethoxyphenylhydrazone (FCCP)-mediated uncoupling. This compromised mitochondrial function in A2780/cis is in agreement with data from the literature.^62^ When both cell lines were treated with [(NHC_1_)_2_Au]­Br, strong OXPHOS inhibition was observed at 0.1 μM, independent of the cell line, and was connected to a dose-dependent decrease in the spare respiratory capacity. As expected, in control cells, the addition of the mitochondrial ATP-synthase inhibitor oligomycin blocked ATP production from the respiration chain, forcing the cells to shift toward aerobic glycolysis. Indeed, both cell models enhanced the extracellular acidification rate (ECAR), indicative of lactate release. Noteworthily, ECAR was already enhanced upon [(NHC_1_)_2_Au]­Br treatment and did not further increase following oligomycin addition (Figure 7B). This indicates that the switch to maximal aerobic glycolysis was already induced under [(NHC_1_)_2_Au]Br treatment before the Seahorse analysis.

**Figure 7:**
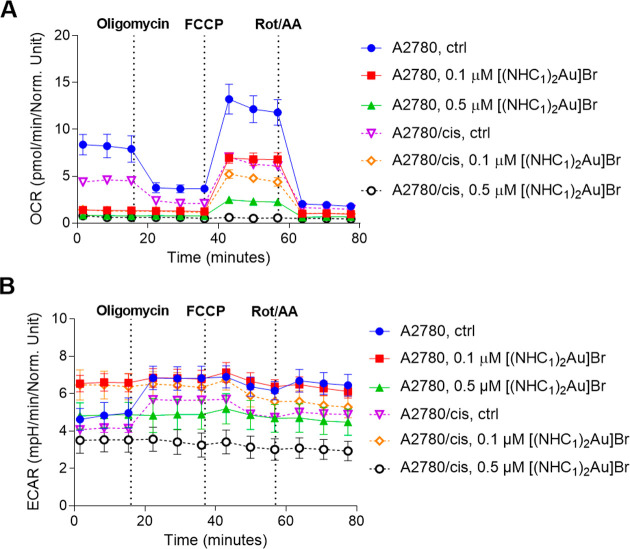
Mitochondrial respiration and aerobic glycolysis. (A) Oxygen consumption rate (OCR) and (B) extracellular acidification rate (ECAR) in a mitochondria stress test of A2780 and A2780/cis cells treated with 0.1 μM or 0.5 μM [(NHC_1_)_2_Au]Br or the solvent control (ctrl) for 24 h before the start of the Seahorse analyses. Measurements are from a real-time Seahorse experiment (Mito stress test) performed under basal conditions and in response to the mitochondrial inhibitors oligomycin (1.5 μM), FCCP (1.0 μM), and rotenone/antimycin A (0.5 μM).

Based on the indications that [(NHC_1_)_2_Au]­Br inhibits the development of OXPHOS, we hypothesized that cells become dependent on efficient aerobic glycolysis to stabilize their metabolic homeostasis and ATP production. Glycolysis can be efficiently inhibited by 2-deoxy- *d* -glucose (2DG). Indeed, A2780/cis cells per se were more sensitive to 2DG treatment (Figure 8A), in agreement with the lower glycolysis levels seen in the Seahorse assay. Even more importantly, the combination of 2DG with [(NHC_1_)_2_Au]Br resulted in distinct synergistic effects (Figure 8B,C), pointing toward synthetic lethality. As this combination was more toxic in A2780/cis cells, this suggests that the resistant subclone lacks the required metabolic flexibility to compensate for [(NHC_1_)_2_Au]­Br-induced OXPHOS inhibition. This elegantly explains the observed collateral sensitivity of this subclone toward [(NHC_1_)_2_Au]Br and indicates a special vulnerability of highly glycolysis-dependent cells to the NHC-gold compound developed in this study.

**Figure 8:**
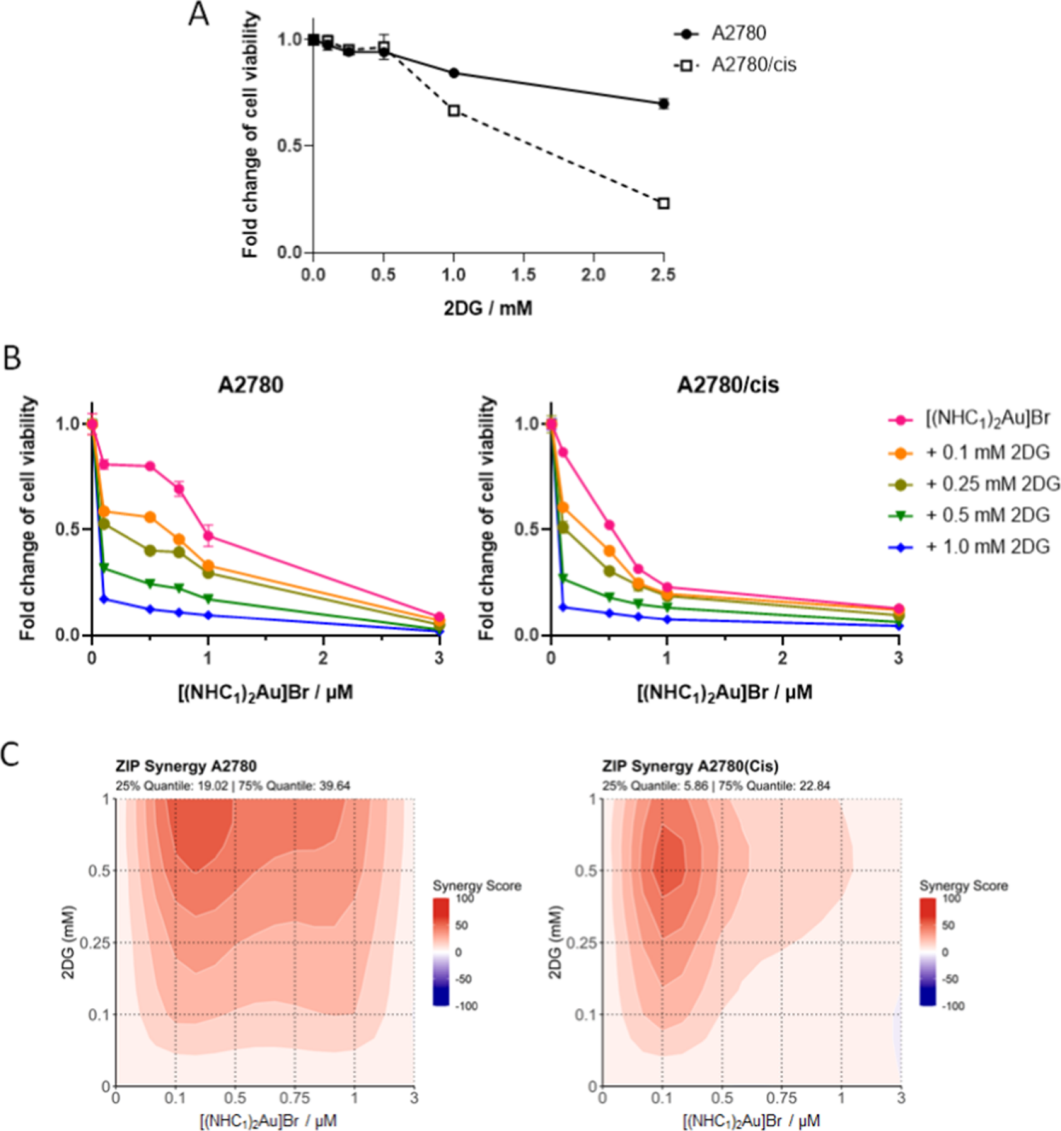
(A) Sensitivity of the A2780/cis cells against 2DG and (B) concentration–response curves of the combination of [(NHC_1_)_2_Au]Br with 2DG. Note that each 2DG-mono-treated control is set to 1. Anticancer activity was evaluated in A2780 vs A2780/cis cells by the MTT viability assay after 72 h of drug incubation. Values are given as mean ± SD calculated from triplicates of one representative experiment. (C) ZIP synergy contour plot of [(NHC_1_)_2_Au]Br and 2DG in A2780 and A2780/cis cells. The plots show the synergy distribution across a dose–response matrix based on the Zero Interaction Potency (ZIP) model. Positive scores (red areas) indicate synergistic interactions. Combination effects were derived from cell viability measurements using the MTT assay after 72 h of drug incubation.

## Conclusion

Treatment failure due to platinum resistance remains a major limitation in OC therapy. Thus, the development of improved therapeutic strategies is urgently needed. There is already some evidence that silver and gold complexes possess potential activity against OC.^35,47,61,63–65^ In particular, NHC complexes have attracted considerable attention. Interestingly, although NHC ligands can be used for both silver and gold complexes, only a few studies have directly compared the structurally identical silver and gold compounds,^15–17,20,21^ and none have focused specifically on overcoming platinum resistance. Consequently, in the present study, eight silver- and gold-based NHC compounds were synthesized to investigate structure–activity relationships and assess their potential to treat platinum-resistant OC. All silver complexes exhibited comparable behavior in solution, regardless of the number of NHC ligands (mono- or bis-compounds) or the nature of the ligand (NHC_1_ or NHC_2_), showing similar TrxR inhibition potential and antiproliferative activity in cell culture. Among them, NHC_2_–Ag–Br emerged as the most effective derivative. In contrast, the gold compounds displayed marked differences in physicochemical and biological properties, which were influenced by both the number of NHC ligands and the ligand type. Indeed, [(NHC_2_)_2_Au]Br exhibited reactivity toward cysteine residues and inhibited TrxR, whereas [(NHC_1_)_2_Au]Br did not, indicating distinct differences in their modes of action.

Noteworthily, while the silver complexes were not affected by Cisplatin resistance, [(NHC_2_)_2_Au]Br shared some cross-resistance with Auranofin in the cisplatin-resistant A2780/cis subclone. In sharp contrast, collateral sensitivity was observed for [(NHC_1_)_2_Au]­Br. Moreover, [(NHC_1_)_2_Au]­Br showed the highest selectivity index when tested toward nontumorigenic cells, identifying it as the most promising candidate among the tested compounds. Notably, the only structural difference between [(NHC_1_)_2_Au]Br and [(NHC_2_)_2_Au]­Br is the presence of two chloro substituents in the NHC backbone, leading to profoundly different biological profiles. Cellular accumulation revealed similar uptake levels among the silver compounds. In contrast, the mono-NHC–gold complexes had a strongly reduced accumulation, well in agreement with their reduced antiproliferative effects. [(NHC_1_)_2_Au]Br had the highest cellular accumulation, being similar in both parental and resistant cells, whereas the lowest intracellular gold levels were seen after treatment with [(NHC_2_)_2_Au]­Br. Noteworthily, subsequent analysis revealed that the distinct differences in cellular drug accumulation can be attributed to active efflux by the copper efflux transporters ATP7A/B. Interestingly, it seems that there is only one report on a gold­(III) compound, Au­[(py^b^-H)­(PTA)­Cl]­PF_6_, where a similar ATP7A/B-mediated efflux was described.^51^ This indicates that the impact of these efflux transporters on NHC complexes in general definitely needs to be better characterized in future studies to better understand their role in the biological activity of this compound class.

To further understand the differential effects of the two bis-NHC–gold compounds, proteomic analysis was performed. The data indicated that both compounds had comparable effects in the parental cell line but elicited distinctly different responses in the resistant A2780/cis subclone. Specifically, [(NHC_2_)_2_Au]Br treatment enriched GOBP terms associated with cellular transport (which is in good agreement with the indications for active [(NHC_2_)_2_Au]Br efflux seen in the drug accumulation experiments), whereas [(NHC_1_)_2_Au]Br significantly influenced metabolic pathways. Further validation using Seahorse Mito Stress analysis revealed a strong OXPHOS inhibition by [(NHC_1_)_2_Au]­Br, which induced a metabolic shift toward aerobic glycolysis. Given that A2780/cis cells are already glycolytically adapted compared to their parental counterpart, the additional stress imposed by [(NHC_1_)_2_Au]Br led to an energy collapse, resulting in collateral sensitivity. Noteworthily, there are also some other gold compounds where an impact on the mitochondrial activity, and especially, OXPHOS inhibition has been reported.^66–68^

In general, our data are in good agreement with the already available literature on the structure–activity relationships of silver– and gold–NHC compounds. Mono-NHC and bis-NHC complexes of silver­(I) and gold­(I) exhibit markedly different physicochemical properties and biological activities (e.g., TrxR inhibition properties), reflecting both the nature of the metal center and the ligand architecture. In the literature, silver­(I) mono-NHC complexes generally show high cytotoxicity due to facile dissociation and release of Ag^+^ ions, leading to oxidative stress, broad protein modification, and disruption of redox homeostasis, including thioredoxin and peroxiredoxin systems.^15,20^ In contrast, gold­(I)–mono-NHC complexes are more kinetically stable, and their cytotoxic effects are supposed to be predominantly mediated through selective inhibition of thiol-dependent enzymes, particularly TrxR, resulting in controlled induction of oxidative stress.^10,17,26^ Bis-NHC complexes of both metals were reported to further enhance stability, but gold–bis-NHC complexes consistently demonstrate superior antiproliferative potency compared to their silver counterparts, likely due to stronger Au–carbene bonds, and more specific targeting of mitochondrial and redox-sensitive pathways.^11,12,15,17,19,21^ In our hands, when directly comparing silver and gold analogues, the mono-NHC–silver complexes were approximately 10-fold more active than the mono-NHC–gold compounds, which was found to be due to higher cellular accumulation of the mono-NHC–silver when compared to the mono-NHC–gold. Furthermore, the monocomplexes exhibited consistent activity regardless of the NHC ligand. However, for the bis-NHC–gold compounds, the presence of NHC_1_ resulted in enhanced activity compared to NHC_2_. The bis-NHC–silver compound had a comparable activity to its respective bis-NHC–gold analogue.

Mechanistic studies using proteomics and NMR metabolomics have shown that gold–bis-NHC complexes induce extensive mitochondrial impairment, modulate glycolytic enzymes, and trigger metabolic shifts toward glycolysis, whereas silver–bis-NHC complexes retain potent TrxR inhibition and oxidative stress induction but with less pronounced mitochondrial effects.^20,69^ Interestingly, also in the case of [(NHC_1_)_2_Au]­Br, the reduction on OXPHOS resulted in enhanced glycolysis. However, in contrast to suggestions regarding other gold–NHC complexes linking these effects to TrxR1 inhibition (e.g., Gao et al.^66^), [(NHC_1_)_2_Au]Br was the weakest TrxR1 inhibitor of our compound panel, indicating that these effects are not connected to inhibition of this enzyme but to other cellular targets.

Noteworthily, when comparing gold–NHC complexes with the clinically applied Auranofin, they exhibit fundamentally different mechanistic behaviors, reflecting the strong electronic donation and enhanced stability of NHC ligands compared to the phosphine–thioglucose structure of Auranofin. While Auranofin is well known to disrupt thiol-based redox systems, most notably by inhibiting TrxR, NMR metabolomics data from Ghini et al.^70^ showed that this interaction leads to pronounced glutathione dysregulation and oxidative stress responses in A2780 cancer cells. In contrast, studies of gold–NHC complexes revealed distinct cellular effects, including broader mitochondrial impairment and altered metabolic routing, which differ from the metabolic signature characteristic of Auranofin. Consequently, the comparative NMR analysis by Ghini et al.,^69^ comparable to our data, underscores that gold-based anticancer agents generate compound-specific metabolic fingerprints.^71^ This supports the view that gold–NHC drugs operate through mechanistic pathways that only partially overlap with those of Auranofin and in several aspects diverge significantly. This also impacts the respective resistance profile of the drugs.

Overall, the role of cellular metabolism in cancer chemotherapy has been gaining attention over the last years,^72–75^ and there is growing evidence that metallodrugs can interfere with mitochondria and metabolic pathways.^76–78^ This study uncovers a less frequently discussed mechanism of cisplatin resistance: a shift toward Warburg-like metabolic phenotype.^79,80^ This adaptation represents a potential vulnerability in platinum-resistant OC that can be therapeutically exploited. The novel gold complex [(NHC_1_)_2_Au]Br presented here stands out as the first promising candidate for further evaluation in preclinical settings for treatment of this resistant OC subtype. Finally, these findings also underscore the importance of systematically evaluating the metabolism-targeting potential of metallodrugs in future studies.

## Experimental Section

### Materials and Methods

All solvents and reagents were obtained from commercial suppliers and used without further purification. Chloro­(dimethylsulfide)­gold­(I) and 1-benzyl-3-methyl-imidazolium bromide (NHC_1_ imidazolium salt) were purchased from BLD Pharm. Compounds were prepared at the Institute of Inorganic Chemistry and characterized at the Faculty of Chemistry, both at the University of Vienna (Vienna, Austria). Elemental analyses were performed on a PerkinElmer 2400 CHN elemental analyzer and are within ±0.4% (purity is >95%). Electrospray ionization (ESI) mass spectra were recorded on a Bruker amaZon SL ion trap mass spectrometer in positive mode by direct infusion using a mixture of ACN/MeOH +1% H_2_O. One-dimensional ^1^H NMR spectra of the precursors were recorded on a Bruker Avance III 500 MHz spectrometer at 298 K. One-dimensional ^1^H NMR and ^13^C NMR spectra of the final products were recorded on a Bruker Avance III 600 MHz spectrometer at 298 K. For ^1^H NMR spectra, the solvent residual peak was taken as an internal reference (s = singlet, d = doublet, m = multiplet, imi = imidazole, ph = phenyl). For biological studies, if not otherwise specified, all reagents were obtained from Sigma-Aldrich.

### Synthesis of the NHC_2_ Ligand

#### Synthesis of 4,5-Dichloro-1-methyl-imidazole

1.37 g of 4,5-dichloroimidazole (10 mmol) was solubilized in 55 mL of acetonitrile (ACN), and 2.28 g of potassium hydroxide (40 mmol) was added. The mixture was stirred for 2 h. After that, the excess potassium hydroxide was filtered off, and the solution was transferred to a Schlenk flask, where 0.65 mL of methyl iodide (10 mmol) was added under an argon atmosphere. The reaction was stirred overnight, and on the next day, a white precipitate of potassium iodide formed. The precipitate was filtered off, and water (5 mL) was added to quench any residual methyl iodide. The solvent was evaporated, and dichloromethane (DCM) was added to extract the product. The aqueous phase was separated from the organic one in a separation funnel, and the organic phase was dried with magnesium sulfate, which was filtered off. The solvent was evaporated, and the product was dried under vacuum.^81^ Yield: 78%.

^1^H NMR (500 MHz, CDCl_3_): 3.54 (s, 3H, CH_3_), 7.28 (s, 1H, NCHN).

#### Synthesis of 4,5-Dichloro-1-benzyl-3-methyl-imidazolium Bromide

1.18 g of 4,5-dichloro-1-methyl-imidazole (7.8 mmol) obtained previously was solubilized in 30 mL of toluene, followed by the addition of 2 mL of benzyl bromide (15 mmol). The reaction was stirred under reflux for 2 days, which yielded a light-yellow solid. The solid was washed with diethyl ether and dried under vacuum. Yield: 69%.

^1^H NMR (500 MHz, DMSO-*d*_6_): 3.85 (s, 3H, CH_3_), 5.53 (s, 2H, CH_2_), 7.41–7.47 (m, 5H, CH_ph_), 9.55 (s, 1H, NCHN). ^13^C (125 MHz, DMSO-*d*_6_): 35.6 (CH_3_), 51.7 (CH_2_), 128.7 (C_ph_), 129.4 (C_ph_), 129.5 (C_ph_), 133.3 (Cq_ph_), 137.2 (NCHN) (C–Cl are not seen).

### Synthesis of Metal Complexes

#### Synthesis of Bromo­[1-methyl-3-(benzyl)­imidazole-2-ylidene]­silver­(I) (NHC_1_–Ag–Br)

2.80 g of 1-benzyl-3-methyl-imidazolium bromide (11 mmol) was added to a Schlenk flask and solubilized in 30 mL of dry DCM under an argon atmosphere, followed by the addition of 1.22 g of silver oxide (5.5 mmol). The flask was protected from light, and the reaction mixture was stirred overnight. The next day, the content was filtered through Celite. The DCM was evaporated, yielding a light-pink solid, which was resolubilized in DCM and filtered again through Celite (yellow solution). The addition of Et_2_O yielded a white crystalline solid, which was washed with Et_2_O and dried under vacuum. Yield: 73%.

Elemental analysis calcd for C_11_H_12_AgBrN_2_ (%): C, 36.70; H, 3.36; N, 7.78. Found (%): C, 36.42; H, 3.27; N, 7.69. ESI-MS in ACN/MeOH + 1% H_2_O (positive): *m*/*z* 568.15 [(NHC)_2_AgBr + K]^+^. ^1^H NMR (600 MHz, DMSO-*d*_6_): 3.77 (s, 3H, CH_3_), 5.31 (s, 2H, CH_2_), 7.29–7.31 (m, 3H, CH_ph_), 7.34–7.36 (m, 2H, CH_ph_), 7.44 (d, *J* = 1.7 Hz, 1H, CH_imi_), 7.53 (d, *J* = 1.7 Hz, 1H, CH_imi_). ^13^C NMR (151 MHz, DMSO-*d*_6_): 38.1 (CH_3_), 54.0 (CH_2_), 122.1 (CH_imi_), 123.2 (CH_imi_), 127.6 (CH_ph_), 127.9 (CH_ph_), 128.7 (CH_ph_), 137.3 (Cq_ph_), 180.1 (C–Ag). Solubility in DMSO: 143 mM.

#### Synthesis of Bis­[1-methyl-3-(benzyl)­imidazole-2-ylidene]­silver­(I) Bromide ([(NHC_1_)_2_Ag]­Br)

A 96 mg portion of bromo­[1-methyl-3-(benzyl)­imidazole-2-ylidene] silver­(I) (0.25 mmol) was dissolved in 10 mL of DCM, and 69 mg of 1-benzyl-3-methyl-imidazolium bromide (0.25 mmol) was added, followed by 74 mg of potassium carbonate (0.5 mmol). The reaction mixture was stirred for 48 h and protected from light. Then, potassium carbonate was filtered through Celite, and the DCM was evaporated, yielding a white solid. The obtained precipitate was stirred in acetone for 3 h for purification (ligand excess can be washed out in acetone, while the final product is insoluble in acetone). The white solid was filtered, washed with acetone, and dried under vacuum. Yield: 67%.

Elemental analysis Calcd for C_22_H_24_AgBrN_4_·0.25 H_2_O (%): C, 49.23; H, 4.58; N, 10.44. Found (%): C, 49.21; H, 4.50; N, 10.46. ESI-MS in ACN/MeOH + 1% H_2_O (positive): *m*/*z* 451.11 [Ag­(NHC)_2_]^+^. ^1^H NMR (600 MHz, DMSO-*d*_6_): 3.77 (s, 3H, CH_3_), 5.34 (s, 2H, CH_2_), 7.27–7.33 (m, 5H, CH_ph_), 7.46 (d, *J* = 1.7 Hz, 1H, CH_imi_), 7.55 (d, *J* = 1.7 Hz, 1H, CH_imi_). ^13^C NMR (151 MHz, DMSO-*d*_6_): 38.1 (CH_3_), 53.9 (CH_2_), 122.3 (CH_imi_), 123.2 (CH_imi_), 127.5 (CH_ph_), 127.9 (CH_ph_), 128.7 (CH_ph_), 137.4 (Cq_ph_), 180.6 (C–Ag). Solubility in DMSO: 23.2 mM.

#### Synthesis of Bromo­[1-methyl-3-(benzyl)­imidazole-2-ylidene]­gold­(I) (NHC_1_–Au–Br)

270 mg of 1-benzyl-3-methyl-imidazolium bromide (1.0 mmol) was solubilized in 15 mL of ACN, followed by the addition of 300 mg (1.0 mmol) of chloro­(dimethylsulfide)­gold­(I) and 277 mg (2.0 mmol) of potassium carbonate. The mixture was stirred at room temperature for 24 h. After that, the potassium carbonate was filtered through Celite, and the ACN was evaporated. A small amount of DCM was added, and the product was purified by column chromatography (silica) using DCM as the mobile phase. After purification, the DCM volume was reduced, and hexane was added, yielding white crystalline needles, which were dried under vacuum. Yield: 56%.

Elemental analysis Calcd for C_11_H_12_AuBrN_2_ (%): C, 29.42; H, 2.69; N, 6.24. Found (%): C, 29.14; H, 2.64; N, 6.20. ESI-MS in ACN/MeOH + 1% H_2_O (positive): *m*/*z* 541.21 [Au­(NHC)_2_]^+^. ^1^H NMR (600 MHz, DMSO-*d*_6_): 3.77 (s, 3H, CH_3_), 5.34 (s, 2H, CH_2_), 7.31–7.39 (m, 5H, CH_ph_), 7.47 (d, *J* = 1.9 Hz, 1H, CH_imi_), 7.54 (d, *J* = 1.9 Hz, 1H, CH_imi_). ^13^C NMR (151 MHz, DMSO-*d*_6_): 37.6 (CH_3_), 53.5 (CH_2_), 121.6 (CH_imi_), 123.2 (CH_imi_), 127.5 (CH_ph_), 128.0 (CH_ph_), 128.7 (CH_ph_), 136.7 (Cq_ph_), 172.2 (C–Au). Solubility in DMSO: 236 mM.

#### Synthesis of Bis­[1-methyl-3-(benzyl)­imidazole-2-ylidene]­gold­(I) Bromide ([(NHC_1_)_2_Au]­Br)

A 92 mg portion of bromo­[1-methyl-3-(benzyl)­imidazole-2-ylidene]­gold­(I) (0.2 mmol) was dissolved in 10 mL of acetone. Next, 52 mg of 1-benzyl-3-methyl-imidazolium-bromide (0.2 mmol) was added, followed by 56 mg of potassium carbonate (0.4 mmol). The reaction mixture was stirred at room temperature over the weekend (∼70 h). Subsequently, DCM (10 mL) was added, and potassium carbonate was filtered through Celite and washed with DCM. The solvent was evaporated, and a white solid appeared. This solid was solubilized in a small amount of DCM, and hexane was added for precipitation. The afforded white precipitate was filtered, washed with hexane, and dried under vacuum. Yield: 89%.

Elemental analysis Cald for C_22_H_24_AuBrN_4_ (%): C, 42.53; H, 3.89; N, 9.02. Found (%): C, 42.15; H, 3.82; N, 8.81. ESI-MS in ACN/MeOH + 1% H_2_O (positive): *m*/*z* 541.21 [Au­(NHC)_2_]^+^. ^1^H NMR (600 MHz, DMSO-*d*_6_): 3.81 (s, 3H, CH_3_), 5.38 (s, 2H, CH_2_), 7.28–7.34 (m, 5H, CH_ph_), 7.53 (d, *J* = 1.8 Hz, 1H, CH_imi_), 7.63 (d, *J* = 1.8 Hz, 1H, CH_imi_). ^13^C NMR (151 MHz, DMSO-*d*_6_): 37.5 (CH_3_), 53.4 (CH_2_), 122.4 (CH_imi_), 123.6 (CH_imi_), 127.4 (CH_ph_), 128.0 (CH_ph_), 128.7 (CH_ph_), 137.0 (Cq_ph_), 183.0 (C–Au). Solubility in DMSO: 77 mM.

#### Synthesis of Bromo­[4,5-dichloro-1-methyl-3-(benzyl)­imidazole-2-ylidene]­silver­(I) (NHC_2_–Ag–Br)

A 163 mg portion of 4,5-dichloro-1-benzyl-3-methyl-imidazolium bromide (0.5 mmol) was solubilized in 15 mL of dry DCM under an argon atmosphere. Then, 65 mg (0.25 mmol) of silver oxide was added, and the mixture was stirred at room temperature under an argon atmosphere overnight, with the flask being protected from light. The next day, the silver oxide excess was filtered through Celite, and the solvent was evaporated. As the product was still dark (due to silver reduction), it was filtered again through Celite by adding a small amount of the solvent. Cold Et_2_O was added to start precipitation, yielding a white solid, which was washed with Et_2_O and dried under vacuum. Yield: 52%.

Elemental analysis Calcd for C_11_H_10_AgBrCl_2_N_2_ (%): C, 30.81; H, 2.35; N, 6.53. Found (%): C, 30.57; H, 2.29; N, 6.39. ESI-MS in ACN/MeOH + 1% H_2_O (positive): *m*/*z* 588.97 [Ag­(NHC)_2_]^+^. ^1^H NMR (600 MHz, DMSO-*d*_6_): 3.81 (s, 3H, CH_3_), 5.44 (s, 2H, CH_2_), 7.28–7.36 (m, 5H, CH_ph_). ^13^C NMR (151 MHz, DMSO-*d*_6_): 37.7 (CH_3_), 53.2 (CH_2_), 117.8 (C–Cl), 116.5 (C–Cl), 127.2 (CH_ph_), 128.2 (CH_ph_), 128.8 (CH_ph_), 135.4 (Cq_ph_), 181.9 (C–Ag). Solubility in DMSO: 37.6 mM.

#### Synthesis of Bis­[4,5-dichloro-1-methyl-3-(benzyl)­imidazole-2-ylidene]­silver­(I) Bromide ([(NHC_2_)_2_Ag]­Br)

130 mg of bromo­[4,5-dichloro-1-methyl-3-(benzyl)­imidazole-2-ylidene]­silver­(I) (0.3 mmol) was dissolved in 15 mL of DCM. Next, 100 mg of 4,5-dichloro-1-benzyl-3-methyl-imidazolium bromide (0.3 mmol) was added, followed by 90 mg of potassium carbonate (0.6 mmol). The reaction mixture was protected from light and stirred at room temperature for 48 h. After that, the potassium carbonate was filtered off through Celite, which was washed with DCM. After solvent evaporation, the remaining white solid was stirred for 3 h in acetone to eliminate any ligand excess, filtered, washed with acetone, and dried under vacuum. Yield: 88%.

Elemental analysis Calcd for C_22_H_24_AgBrCl_2_N_4_·0.25 H_2_O (%): C, 39.17; H, 3.06; N, 8.31. Found (%): C, 38.79; H, 2.90; N, 8.30. ESI-MS in ACN/MeOH + 1% H_2_O (positive): *m*/*z* 588.98 [Ag­(NHC)_2_]^+^. ^1^H NMR (600 MHz, DMSO-*d*_6_): 3.80 (s, 3H, CH_3_), 5.47 (s, 2H, CH_2_), 7.25–7.26 (m, 2H, CH_ph_), 7.30–7.32 (m, 3H, CH_ph_). ^13^C NMR (151 MHz, DMSO-*d*_6_): 37.6 (CH_3_), 52.9 (CH_2_), 116.6 (C–Cl), 117.7 (C–Cl), 127.2 (CH_ph_), 128.1 (CH_ph_), 128.8 (CH_ph_), 135.5 (Cq_ph_), 182.9 (C–Ag). Solubility in DMSO: <5 mM.

#### Synthesis of [4,5-Dichloro-1-methyl-3-(benzyl)­imidazole-2-ylidene]­gold­(I) Bromide (NHC_2_–Au–Br)

A 161 mg portion of 4,5-dichloro-1-benzyl-3-methyl-imidazolium bromide (0.5 mmol) was solubilized in 6 mL of ACN, followed by the addition of 151 mg (0.5 mmol) of chloro­(dimethylsulfide)­gold­(I) and 144 mg (1.0 mmol) of potassium carbonate. The mixture was stirred at room temperature for ∼24 h. The potassium carbonate was filtered through Celite, and the ACN was evaporated. A small amount of DCM was added, and the product was purified by column chromatography (silica) using DCM as the mobile phase. After purification, the DCM volume was reduced, and hexane was added, yielding a white crystalline powder, which was dried under vacuum. Yield: 68%.

Elemental analysis Calcd for C_11_H_10_AuBrCl_2_N_2_ (%): C, 25.51; H, 1.95; N, 5.41. Found (%): C, 25.24; H, 1.88; N, 5.29. ESI-MS in ACN/MeOH + 1% H_2_O (positive): *m*/*z* 478.05 [(NHC)_2_Au + ACN]^+^; 679.06 [Au­(NHC)_2_]^+^. ^1^H NMR (600 MHz, DMSO-*d*_6_): 3.82 (s, 3H, CH_3_), 5.47 (s, 2H, CH_2_), 7.34–7.36 (m, 3H, CH_ph_), 7.39–7.41 (m, 2H, CH_ph_).^13^C NMR (151 MHz, DMSO-*d*_6_): 37.1 (CH_3_), 52.9 (CH_2_), 116.3 (C–Cl), 117.9 (C–Cl), 127.1 (CH_ph_), 128.3 (CH_ph_), 128.8 (CH_ph_), 134.8 (Cq_ph_), 173.7 (C–Au). Solubility in DMSO: 41 mM.

#### Synthesis of Bis­[4,5-dichloro-1-methyl-3-(benzyl)­imidazole-2-ylidene]­gold­(I) Bromide ([(NHC_2_)_2_Au]­Br)

A 26.5 mg portion of [4,5-dichloro-1-methyl-3-(benzyl)­imidazole-2-ylidene]­gold­(I) bromide (0.05 mmol) was dissolved in 6 mL of acetone. Next, 18.7 mg of 4,5-dichloro-1-benzyl-3-methyl-imidazolium bromide (0.05 mmol) was added, followed by 15.8 mg of potassium carbonate (0.1 mmol). The reaction mixture was stirred at room temperature for 3 days. After 72 h, the potassium carbonate was filtered off through Celite and washed with ACN. The solvent was evaporated, and the obtained light-yellow solid was filtered, washed with hexane, and dried under vacuum. Yield: 77%.

Elemental analysis Calcd for C_22_H_24_AuBrCl_2_N_4_ (%): C, 34.81; H, 2.66; N, 7.38. Found (%): C, 34.64; H, 2.64; N, 7.33. ESI-MS in ACN/MeOH + 1% H_2_O (positive): *m*/*z* 679.07 [Au­(NHC)_2_]^+^. ^1^H NMR (600 MHz, DMSO-*d*_6_): 3.84 (s, 3H, CH_3_), 5.49 (s, 2H, CH_2_), 7.27–7.28 (m, 2H, CH_ph_), 7.31–7.32 (m, 3H, CH_ph_).^13^C NMR (151 MHz, DMSO-*d*_6_): 37.1 (CH_3_), 52.6 (CH_2_), 117.3 (C–Cl), 118.7 (C–Cl), 127.1 (CH_ph_), 128.3 (CH_ph_), 128.4 (CH_ph_), 134.9 (Cq_ph_), 182.4 (C–Au). Solubility in DMSO: 42 mM.

### Stability Studies by UV–Vis Spectroscopy

For checking the stability under physiological conditions, all complexes were investigated with an Agilent 8453 UV–vis spectrophotometer (Agilent Technologies, Germany) using 10 mm path-length quartz cuvettes. Ten mM stock solutions of the compounds in DMSO were diluted to final concentrations of 100 μM in 30% DMSO in PB (pH 7.4). Compounds were incubated at 37 °C, and spectra were measured at 0, 1, 3, 6, 12, and 24 h. NHC_1_–Ag–Br and NHC_1_–Au–Br were additionally measured under these conditions in cycles (each cycle, ∼1 min).

### Stability Studies by HPLC–MS

[(NHC_1_)_2_Au]Br and [(NHC_2_)_2_Au]Br were evaluated by HPLC–MS. Ten mM stock solutions (10 mM) in DMSO were diluted to final concentrations of 10 μM in PB (pH 7.4) and RPMI and incubated at 37 °C for up to 24 h. In addition, incubation with 5 equiv of l-cysteine in PB was performed following the same procedure. The experiments were monitored on an Agilent 1260 Infinity system using a Waters Acquity UPLC HSS T3 Column 50 mm × 3 mm coupled to an Agilent 6230 Time of Flight mass spectrometer. Milli-Q water containing 0.1% formic acid and ACN containing 0.1% formic acid were used as eluents. A gradient of 5–95% ACN in 5 min was used, with a total run time of 9 min.

### Inhibition of Mammalian TrxR

To determine the inhibition of mammalian TrxR, an established microplate reader-based assay was performed. For this purpose, commercially available recombinant human TrxR (from SeLENOZYME) was used and diluted with distilled water to achieve a concentration of 0.15 U/mL. The compounds were freshly dissolved as stock solutions in DMSO. A volume of 25 μL aliquots of the enzyme solution and 25 μL of potassium phosphate buffer (pH 7.0) containing the compounds in graded concentrations (1% DMSO) were mixed. Positive controls: 25 μL aliquots of the enzyme solution mixed with 25 μL of 1% DMSO in buffer solution (no compounds). The final concentration of DMSO was 0.5% v/v in all samples. Blank solution: the highest used concentration of compound in 0.5% DMSO in buffer solution (no enzyme). All resulting solutions were incubated with moderate shaking for 75 min at 37 °C in a 96-well plate. To each well, 50 μL of the reaction mixture [1 mL of the reaction mixture consists of 930 μL of potassium phosphate buffer (50 mM, pH 7.0), 10 μL of ethylenediaminetetraacetic acid (EDTA) solution (100 mM, pH 7.5), 20 μL of bovine serum albumin (BSA) solution (0.2%), and 40 μL of nicotinamide adenine dinucleotide phosphate (NADPH) solution (25 mM)] were added, and the reaction was started immediately by addition of 25 μL of a 20 mM ethanolic 5,5′-dithiobis­(2-nitrobenzoic acid) solution. After proper mixing, the formation of 2-nitro-5-thiobenzoate (5-TNB) was monitored with a microplate PerkinElmer 2030 Multilabel Reader VICTORX4 at 405 nm, 10 times in 35 s intervals for about 6 min. The increase in 5-TNB concentration over time followed a linear trend (r2 > 0.990), and the enzymatic activities were calculated as the slopes (increase in absorbance per second) thereof. For each tested compound, the noninterference with the assay components was confirmed, as there was no 2-nitro-5-thiobenzoate formation with the blank solution. The IC_50_ values were calculated as the concentration of compound that decreased the enzymatic activity of the untreated control by 50% and are given as the means and error of three repeated experiments.

### Cell Culture

The human OC cell line A2780 and its cisplatin-resistant subline A2780/cis (from Sigma-Aldrich, MO, USA), in addition to the nontumorigenic human skin fibroblast cell line Hs545SK (ATCC; CRL-7318), were used. A2780 parental and resistant cells were grown in an RPMI-1640 cell culture medium supplemented with 10% fetal bovine serum (FBS), while Hs545SK cells were grown in DMEM with 10% FBS. Cell cultures were periodically checked for contamination. Cultures were maintained at 37 °C in a humidified atmosphere with 5% CO_2_. Resistance of A2780/cis was maintained by selecting the subclones once a week with 1.0 μM cisplatin. A revertant line was generated by leaving A2780/cis cells for over a month without selection (A2780/cis-REV).

### Viability Assays

Cells were seeded (5 × 10^4^ cells/well for A2780 and Hs545SK and 7 × 10^4^ cells/well for A2780/cis and A2780/cis-REV) in 100 μL/well in 96-well plates and allowed to attach at 37 °C and 5% CO_2_ for 24 h. Compounds were diluted in DMSO (stock solutions of 10 mM) and then further diluted in a growth medium (DMSO concentration <1%). Drug dilutions were added in 100 μL/well. For modulator experiments using 2DG in combination with [(NHC_1_)_2_Au]­Br, drugs were added 50 μL/well. After drug treatment, cells were incubated for 72 h at 37 °C and 5% CO_2_. The proportion of viable cells was determined by a 3-(4,5-dimethylthiazole-2-yl)-2,5-diphenyltetrazolium assay (MTT) following the manufacturer’s recommendations (EZ4U, Biomedica, Vienna, Austria). Anticancer activity was expressed as IC_50_ values (drug concentrations inducing a 50% reduction of cell survival in comparison to the control) calculated from full dose–response curves using GraphPad Prism software. Results are expressed as the mean ± the SD of three independent experiments.

### Cellular Ag and Au Uptake Levels

A2780 and A2780/cis cells (1 × 10^6^/well) were seeded into six-well plates, allowed to settle for 24 h, and exposed to the drugs in 5 μM for 5 h at 37 and 4 °C (the last temperature only for treatments with silver nitrate, [(NHC_1_)_2_Ag]­Br, [(NHC_1_)_2_Au]­Br, and [(NHC_2_)_2_Au]­Br), in triplicate. Cells were trypsinized, counted, and washed once with cold PB or PBS. Cells treated with silver compounds were then lysed at room temperature in 300 μL of HNO_3_ (≥69%, Rotipuran Supra, Carl Roth, Karlsruhe, Germany), being diluted in 5.7 mL of ultrapure water (18.2 MΩcm, Milli-Q Advantage, Darmstadt, Germany). Cells treated with gold compounds were lysed in a mixture of 68 μL of HCl (30%, Rotipuran Supra, Carl Roth, Karlsruhe, Germany) and 370 μL of HNO_3_ and diluted in 8.5 mL of ultrapure water. The metal concentrations (μg/L) were determined by ICP-MS and normalized to the cell amount (ng of metal/10^6^ cells). Statistical analysis was done by paired *t* tests. Results are expressed as the mean ± SD of at least two independent experiments. The measurements were performed on an Agilent 7800 ICP-QMS instrument (Agilent Technologies, Tokyo, Japan) equipped with an Agilent SPS 4 autosampler (Agilent Technologies, Tokyo, Japan) and a MicroMist nebulizer at a sample uptake rate of approximately 0.2 mL min^–1^. The Agilent MassHunter software package (Workstation Software, Version C.01.04, 2018) was used for data evaluation. All measured samples were blank-corrected. The instrumental parameters for the ICP-MS are summarized in Table S3. Elemental standard solutions were purchased from Labkings (Hilversum, The Netherlands). The instrument was tuned daily.

### Flow Cytometry

For cell death analysis, A2780 and A2780/cis cells were seeded 5 × 10^5^ cells/well in 6-well plates and treated with the compounds (5 μM) for 24 h. Cells were harvested and stained with annexin-V-APC/PI (APC, allophycocyanin; PI, propidium iodide) (BD Biosciences), and the protocol is described elsewhere.^82^ For mitochondrial membrane potential, A2780 and A2780/cis cells were seeded 5 × 10^5^ cells/well in 6-well plates and treated with the compounds (5 μM) for 24 h. For mitochondrial health evaluation, cells were harvested and stained with 5,5′,6,6′-tetrachloro-1,1′,3,3′-tetraethylbenzimidazolylcarbocyanine iodide (JC-1). A 10 μg/mL solution (in medium) of JC-1 was added to the cells, which were incubated for 15 min at 37 °C and afterward washed with PBS before measurement. In both flow cytometry experiments, cells were measured in the flow cytometer LSR Fortressa (BD Biosciences), and data were analyzed either in BD FACSDiva (BD Biosciences) or in FlowJo software (Treestar). Results are expressed as the mean ± SD of three independent experiments.

### Proteomics

A2780 and A2780/cis cells were seeded 2.5 × 10^5^ cells/well in 6-well plates in sextuplicate and left to recover for 24 h. The next day, cells were treated with either medium, [(NHC_1_)_2_Au]Br or [(NHC_2_)_2_Au]Br (both at 1.0 μM), for 16 h in sextuplicate. The medium was removed from the wells, and cells were washed two times with 1 mL of PBS. Next, 80 μL of 4% w/v sodium deoxycholate in Tris–HCl 2 M (pH 8.5; SDC buffer) was added to each well, and cells were scraped and transferred to Eppendorf tubes. Fifty microliters of SDC buffer were used to wash the wells, and this content was also transferred to the tubes, totaling 130 μL per replicate. The tubes were heated on a heating block and shaken for 5 min at 95 °C and 3.3 *g*. Samples were stored at −20 °C until further processing.

Then, a bicinchoninic acid (BCA) colorimetric assay was used for protein quantification, and the samples were adjusted to 20 μg of protein. The samples were digested according to an in-solution protocol using the StageTip workflow.^83^ In short, peptide samples were reduced with (tris­(2-carboxyethyl)­phosphine) and alkylated with 2-chloroacetamide (1400 rpm, 45 °C). After the mixture reached room temperature, trypsin/Lys-C (1 μL, 0.2 μg^•^μL^–1^, enzyme-to-substrate ratio of 1:100) was added to each sample and incubated overnight (1400 rpm, 30 °C). The samples were dried in a SpeedVac (40 min, 40 °C). The StageTips were prepared by stacking two disks of a polystyrenedivinylbenzene-reversed phase sulfonate material (Empore 2241 SDB-RPS, ∼12 μm particle size, 47 mm; CDS Analytical LLC) into a pipet tip. SDB-RPS loading buffer (99% IPA, 1% TFA) was added to each sample and centrifuged (1500 g, 8 min). Then, loading buffer (100 μL, 99% IPA, 1% TFA) and SDB-RPS wash buffer 2 (100 μL, 94.8% water, 5% ACN, 0.2% TFA) were sequentially added and centrifuged. The peptides were directly eluted into the inlet using SDB-RPS elution buffer (60 μL, 39.8% water, 59.7% ACN, and 0.5% NH_4_OH), followed by centrifugation (1500 g, 5 min). The samples were finally dried in a vacuum concentrator (40 °C) and stored at −20 °C until analysis.

Dried peptide samples were reconstituted in the loading solvent (40 μL, 97.95% water, 2% ACN, 0.05% TFA), and synthetic peptide standards were added. Samples were briefly vortexed and centrifuged (10 000*g*, 5 min). Chromatography was performed using a Dionex UltiMate 3000 RSLCnano system (Thermo Fisher Scientific). The injection volume was 5 μL. The precolumn was an Acclaim PepMap C18 100 (Thermo Fisher Scientific). Peptides were separated on an Aurora emitter column (1.6 μm C18, 25 cm × 75 μm, IonOpticks) by applying a gradient ranging from 12% to 42% solvent B (79.9% ACN, 20% water, 0.1% FA) over the course of 90 min at a flow rate of 300 nL^•^min^–1^. Solvent A was 99.9% water and 0.1% formic acid. Mass spectrometric analysis was performed on a timsTOF Pro (Bruker Daltonics) mass spectrometer running in parallel accumulation-serial fragmentation mode and data-dependent acquisition. An *m*/*z* scan range of 100–1700 was set to acquire MS1 and MS2 spectra.

Data was processed using MaxQuant (version 1.6.17.0) with the Andromeda search engine to enable identification and label-free quantification (LFQ) of proteins and searched against the SwissProt *Homo sapiens* database (14.12.2019 with 20380 canonical entries). False discovery rates for peptide-spectrum match (PSM) and protein were set to 0.01; the “match between runs” setting was enabled with a matching time window of 0.7 min and an alignment time window of 20 min. Further criteria included an MS/MS mass tolerance of 40 ppm and a maximum of two missed cleavages. A minimal requirement for protein identification was set to two peptides, with one of them unique for the protein. Carbamidomethylation of cysteine was set as a fixed modification, while methionine oxidation and the acetylation of the protein *N*-terminus were included in the search as variable modifications. Perseus (ver. 1.6.14.0) was used for filtering and missing value imputation. Proteins only identified by site, common contaminants, and proteins matching reversed sequences were filtered out. LFQ-values of remaining entries were log 2-transformed. Proteins had to be identified in at least 70% of the samples under at least one condition. Entries with missing values were then imputed with values from a normal distribution (downshift: 1.8, width: 0.3). Volcano plots were generated using a two-sided *t* test with a s0 = 0.1, FDR = 0.05, and 250 permutations.

Proteomics data were put into a biological context using the Gene Ontology database. Briefly, data were loaded into the clusterProfiler package of R.^84^ The molecular signature database,^85^ subcollection “gene ontology, and biological process”, was queried using differentially expressed proteins as input for the enricher function of the cluster profiler. Default parameters, except for a *p*-value and *q*-value cutoff of 1, were used. *P*-values were adjusted for multiple testing according to Benjamini–Hochberg.^86^

### Cellular Respiration

Cells were seeded into 96-well plates (XFe96/XF Pro Cell Culture Microplates, Agilent, USA) at a cell density of 8 × 10^4^ cells/well for A2780 and 1 × 10^5^ cells/well for A2780/cis in 80 μL/well of cell culture medium supplemented with 10% FBS and were allowed to settle overnight. Cells were treated either with solvent or with 0.1 or 0.5 μM [(NHC_1_)_2_Au]Br for 24 h. The Seahorse Mito Stress Test (Seahorse XFp Cell Mito Stress Test Kit, Agilent, USA) was used for OCR and ECAR measurement. The assay was performed according to the manufacturer’s recommendations. After the incubation period, the medium was replaced with the Seahorse XF RPMI assay medium (pH 7.4, Agilent, Santa Clara, CA, USA) supplemented with 10 mM glucose and 2 mM glutamine, as well as 1 mM pyruvate, and incubated for 1 h in a CO_2_-free incubator at 37 °C. The kit reagents were sequentially added from the injection ports of the sensor cartridges (XFe96/XF Pro sensor cartridges, Agilent, USA) to a final concentration of 1.5 μM oligomycin, 1.0 μM FCCP, and 0.5 μM rotenone/antimycin A. For cell number quantification, 4 μM Hoechst 33258 (1 mg/mL in PBS, pH 7.4) was added. Following the Seahorse analysis, cells were imaged, and Hoechst fluorescence was measured in the DAPI channel using the Cytation5 Cell Imaging Multimode Reader (BioTek as part of Agilent, Santa Clara, CA, USA) for normalization. Data were processed with the Seahorse Wave Pro Software (version 10.0.1, Agilent, Santa Clara, CA, USA). OCR and ECAR levels are displayed per 1000 cells.

## Supplementary Material





## Data Availability

Proteomic data was submitted to the ProteomeXchange Consortium (https://proteomecentral.proteomexchange.org/) and is available in the PRIDE partner repository with identifier PXD067379.

## Notes

NMR spectra, MS spectra, UV–vis spectra, HPLC-MS chromatograms + spectra, concentration–response curves, ICP–MS data, proteomics data, dot plots, and ICP–MS parameters (PDF)
Molecular formula strings (CSV)

## Abbreviations

2DG,: 2-deoxy- *d* -glucose
APC,: allophycocyanin
BCA,: bicinchoninic acid
BP,: biological process
ECAR,: extracellular acidification rate
FCCP,: carbonyl cyanide-*p*-trifluoromethoxyphenylhydrazone
FDR,: false discovery rate
GO,: Gene Ontology
HMOX1,: heme oxygenase
JC-1,: 5,5′,6,6′-tetrachloro-1,1′,3,3′-tetraethylbenzimi-dazolylcarbocyanine iodide
LQF,: label-free quantification
MMP,: mitochondrial membrane potential
NHC,: *N*-heterocyclic carbine
OC,: ovarian cancer
OCR,: oxygen consumption rate
OXPHOS,: oxidative phosphorylation
PASEF,: parallel accumulation-serial fragmentation
PI,: propidium iodide
RPMI,: Roswell Park Memorial Institute cell culture medium
SDC,: sodium deoxycholate
SI,: selectivity index
TrxR,: thioredoxin reductase
TXNL1,: thioredoxin-like 1
