# Supplementary material for: Structure–Activity Relationships of Silver(I)- and Gold(I)–NHC Complexes Reveal Distinctly Different Responses of Cisplatin-Resistant Ovarian Cancer to Bis-NHC–Gold(I) Derivatives
Source: J Med Chem. 2026 Jan 27;69(3):2462–80. doi: 10.1021/acs.jmedchem.5c02355 (PMC12910662; doi:10.1021/acs.jmedchem.5c02355)
Supplement: Supplementary file 1 [file jm5c02355_si_001.pdf]

Supporting Information File

Structure-activity relationships of silver(I)- and gold(I)-NHC complexes reveal distinctly different responses of cisplatin-resistant ovarian cancer to bis-NHC gold(I) derivatives

Julia H. Bormio Nunes,[1,2,3*] Christina Hacker,[2] Monika Caban,[2] Daniel Valcanover,[2] Patrick A. Yassemipour,[1] Sebastian Türck,[4] Ingo Ott,[4] Lukas Skos,[5] Andrea Bileck,[5,6] Christopher Gerner,[5,6] Samuel M. Meier-Menches,[1,5,6] Thomas Mohr,[2] Walter Berger,[2,3] Christian R. Kowol,[1,3*§] Petra Heffeter[2,3§]

[1]Institute of Inorganic Chemistry, Faculty of Chemistry, University of Vienna, Vienna, 1090, Austria.

[2]Center for Cancer Research, Medical University of Vienna, Vienna, 1090, Austria.

[3]Research Cluster "Translational Cancer Therapy Research", Vienna, 1090, Austria.

[4]Institute of Medicinal and Pharmaceutical Chemistry, Technische Universität Braunschweig, Braunschweig, 38106, Germany.

[5]Department of Analytical Chemistry, Faculty of Chemistry, University of Vienna, Vienna, 1090, Austria

[6]Joint Metabolome Facility, Medical University of Vienna and University of Vienna, Vienna, 1090, Austria.

* Corresponding authors: christian.kowol@univie.ac.at and julia.helena.bormio.nunes@univie.ac.at

§ These authors share last authorship

Table of contents

**Scheme S1**	Synthesis of NHC_2	S3
**Figure S1**	^{1}H-NMR spectra of (A) NHC_1 and (B) NHC_2 in DMSO-d6	S3
**Figure S2**	(A) ^{1}H-NMR spectrum and (B) ^{13}C-NMR spectrum of NHC_1-Ag-Br in DMSO-d6	S4
**Figure S3**	(A) ^{1}H-NMR spectrum and (B) ^{13}C-NMR spectrum of $[(NHC_1)_2Ag]Br$ in DMSO-d6	S5
**Figure S4**	(A) ^{1}H-NMR spectrum and (B) ^{13}C-NMR spectrum of NHC_1-Au-Br in DMSO-d6	S6
**Figure S5**	(A) ^{1}H-NMR spectrum and (B) ^{13}C-NMR spectrum of $[(NHC_1)_2Au]Br$ in DMSO-d6	S7
**Figure S6**	(A) ^{1}H-NMR spectrum and (B) ^{13}C-NMR spectrum of NHC_2-Ag-Br in DMSO-d6	S8
**Figure S7**	(A) ^{1}H-NMR spectrum and (B) ^{13}C-NMR spectrum of $[(NHC_2)_2Ag]Br$ in DMSO-d6	S9
**Figure S8**	(A) ^{1}H-NMR spectrum and (B) ^{13}C-NMR spectrum of NHC_2-Au-Br in DMSO-d6	S10
**Figure S9**	(A) ^{1}H-NMR spectrum and (B) ^{13}C-NMR spectrum of $[(NHC_2)_2Au]Br$ in DMSO-d6	S11
**Figure S10**	MS spectra of silver-NHC compounds (A) $(NHC_1)_2Ag$ and (B) NHC_2-Ag-Br	S12
**Figure S11**	MS spectra of bis-NHC gold compounds (A) $[(NHC_1)_2Au]Br$ and (B) $[(NHC_2)_2Au]Br$	S13

**Figure S12**	MS spectra of mono-NHC gold compounds. (A) NHC_1-Au-Br, (B) NHC_2-Au-Br, and (C) HRMS of NHC_2-Au-Br	S14
**Figure S13**	^{1}H-NMR stability studies of mono-NHC complexes for 48 h in DMSO-d6	S15
**Figure S14**	^{1}H-NMR stability studies of bis-NHC complexes for 48 h in DMSO-d6	S15
**Figure S15**	UV-Vis stability measurements of NHC_1-Ag-Br	S16
**Figure S16**	UV-Vis stability measurements of NHC-gold(I) compounds	S16
**Figure S17**	HPLC-MS measurement of (A+B) $[(NHC_1)_2Au]Br$ and (C+D) $[(NHC_2)_2Au]Br$ at 0 h (black) and after 24 h (red) in PB (10 µM, < 1 % DMSO)	S17
**Figure S18**	HPLC-MS measurement of (A+B) $[(NHC_1)_2Au]Br$ and (C+D+E+F) $[(NHC_2)_2Au]Br$ at 0 h (black) and after 24 h (red) with 5 eq. of L-cysteine (10 µM of gold compound, < 1 % DMSO)	S18
**Figure S19**	HPLC-MS measurement of (A+B) $[(NHC_1)_2Au]Br$ and (C+D+E+F) $[(NHC_2)_2Au]Br$ at 0 h (black) and after 24 h (red) in RPMI (10 µM, < 1 % DMSO)	S19-S20
**Figure S20**	Resistance of A2780/cis cells against mono-NHC silver and gold complexes, Auranofin, and Cisplatin	S20
**Figure S21**	Anticancer activity of NHC_1 and NHC_2	S21
**Figure S22**	Anticancer activity of $AgNO_3$ alone and in combination with the free ligands	S22
**Figure S23**	Activity of Cisplatin and $[(NHC_1)_2Au]Br$ in A2780/cis-REV (revertant) cells	S22
**Figure S24**	Intracellular gold levels after treatment with $[(NHC_2)_2Au]Br$ at 5 µM alone or in combination with 10 µM or 50 µM of $CuCl_2$ in A2780 and A2780/cis cells after 5 h incubation at 37 °C	S23
**Figure S25**	Viability assays of A2780 and A2780/cis cells after 24 h treatment with $[(NHC_1)_2Au]Br$ and $[(NHC_2)_2Au]Br$	S23
**Figure S26**	Top 15 GOBP terms from (A+B) A2780 or (C+D) A2780/cis cells treated with (A+C) $[(NHC_1)_2Au]Br$ or (B+D) $[(NHC_2)_2Au]Br$ compared to the solvent-treated group	S24
**Figure S27**	Dot plots of proteins related to the NRF2-KEAP1 (A) and heat shock (B) stress responses. Both A2780 and A2780/cis cells were treated with vehicle control (CON), $[(NHC_1)_2Au]Br$, or $[(NHC_2)_2Au]Br$ in hexuplicates	S25
**Figure S28**	Dot plots of proteins related to the mitochondrial translation. Both A2780 and A2780/cis cells were treated with vehicle control (CON), $[(NHC_1)_2Au]Br$, or $[(NHC_2)_2Au]Br$ in hexuplicates	S26
**Figure S29**	Dot plots of proteins related to oxidative phosphorylation, especially complex I, complex III and complex V. Both A2780 and A2780/cis cells were treated with vehicle control (CON), $[(NHC_1)_2Au]Br$, or $[(NHC_2)_2Au]Br$ in hexuplicates	S27
**Table S1**	Gene ontology biological processes (GOBP) that are either upregulated in A2780/cis or A2780 are shown, including the number of proteins per term and adjusted p-value according to Benjamini-Hochberg	S27
**Table S2**	Number of identified proteins in each perturbation of $[(NHC_1)_2Au]Br$- and $[(NHC_2)_2Au]Br$-treated parental A2780 and resistant A2780/cis cells	S28
**Table S3**	ICP-MS equipment parameters for the measurement of silver and gold	S28

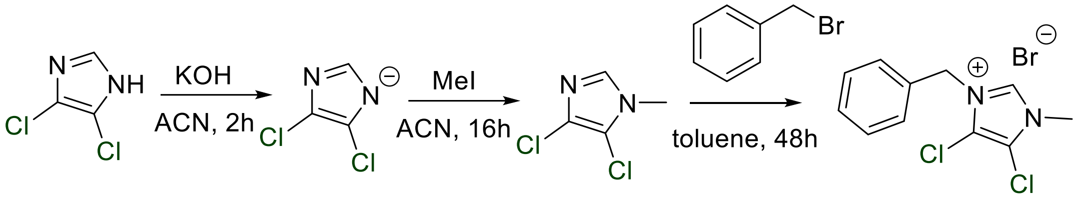

Scheme S1. Synthesis of NHC_2.

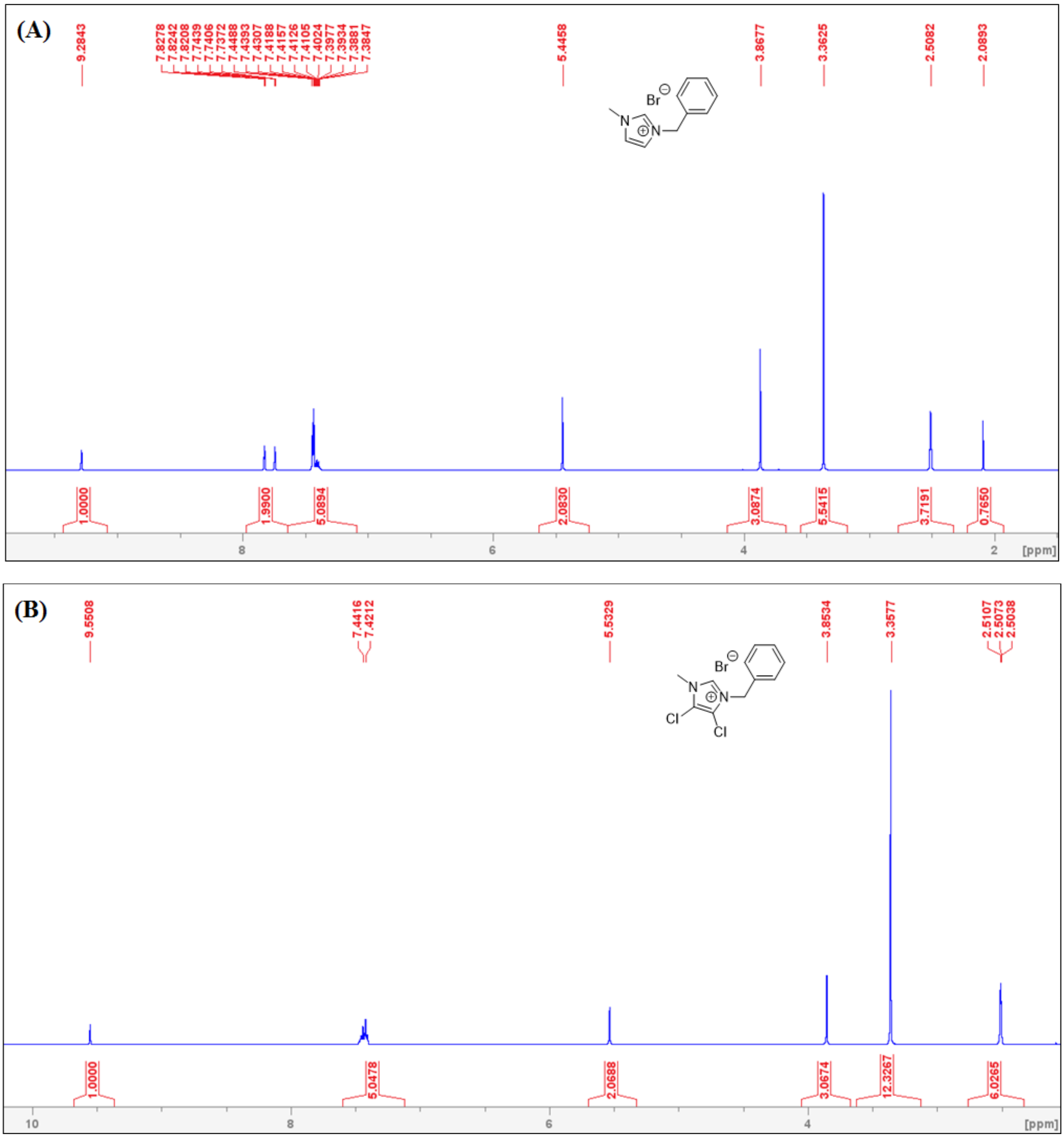

Figure S1. ^{1}H-NMR spectra of (A) NHC_1 and (B) NHC_2 in DMSO-d6.

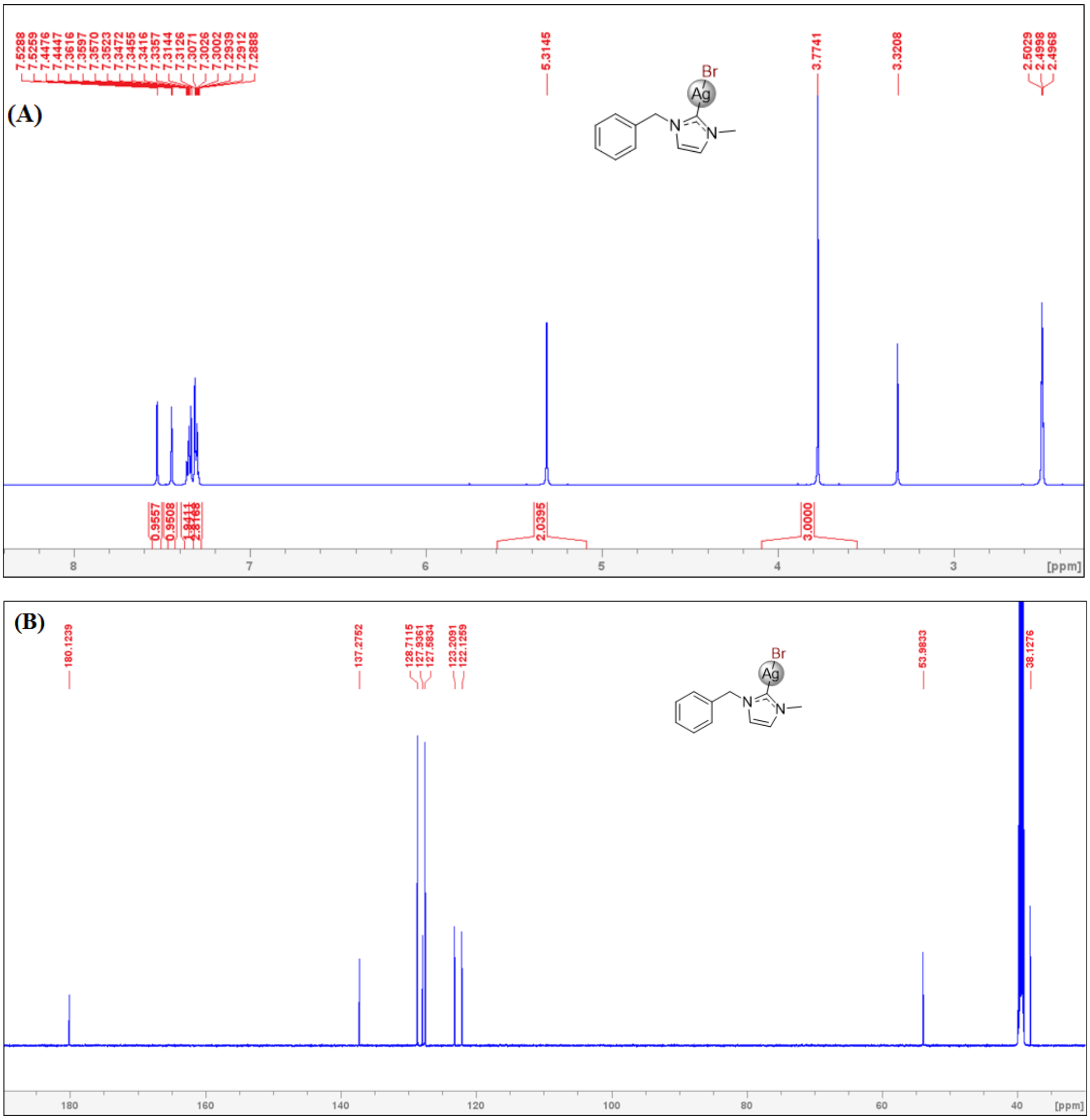

Figure S2. (A) ^{1}H-NMR spectrum and (B) ^{13}C-NMR spectrum of NHC$_1$-Ag-Br in DMSO-d6.

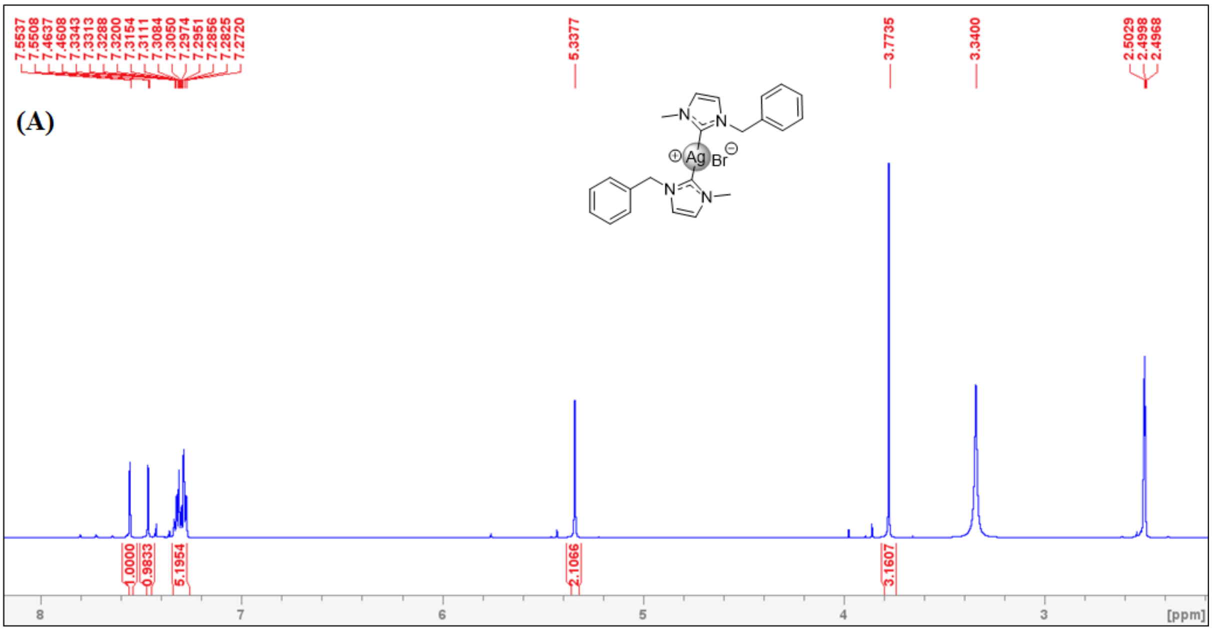

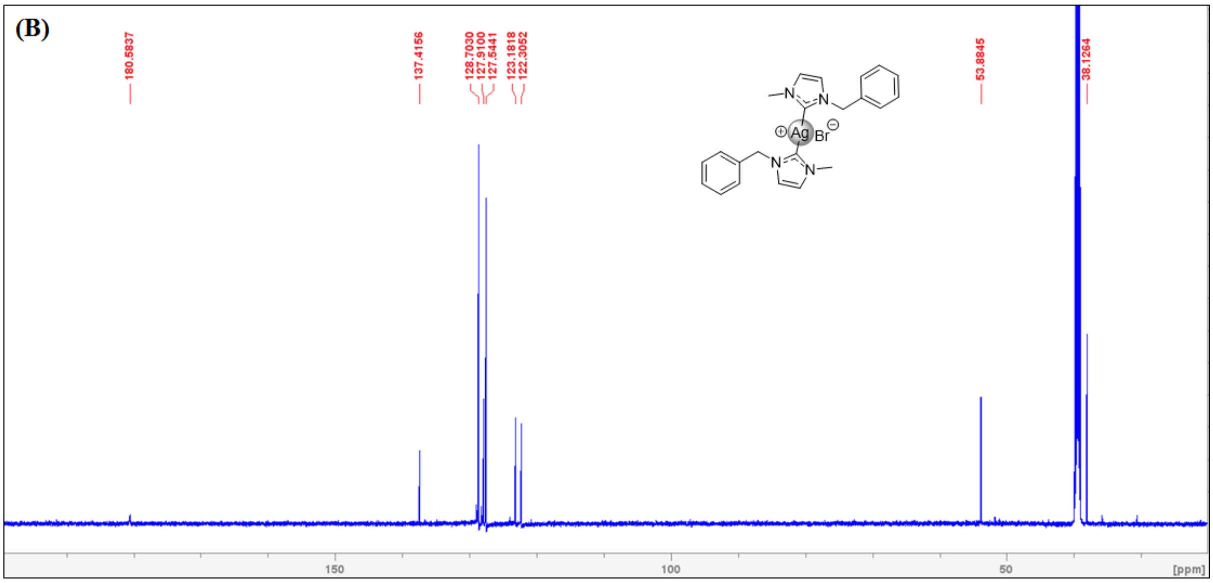

Figure S3. (A) ^{1}H-NMR spectrum and (B) ^{13}C-NMR spectrum of $[(NHC_1)_2Ag]Br$ in DMSO-d6.

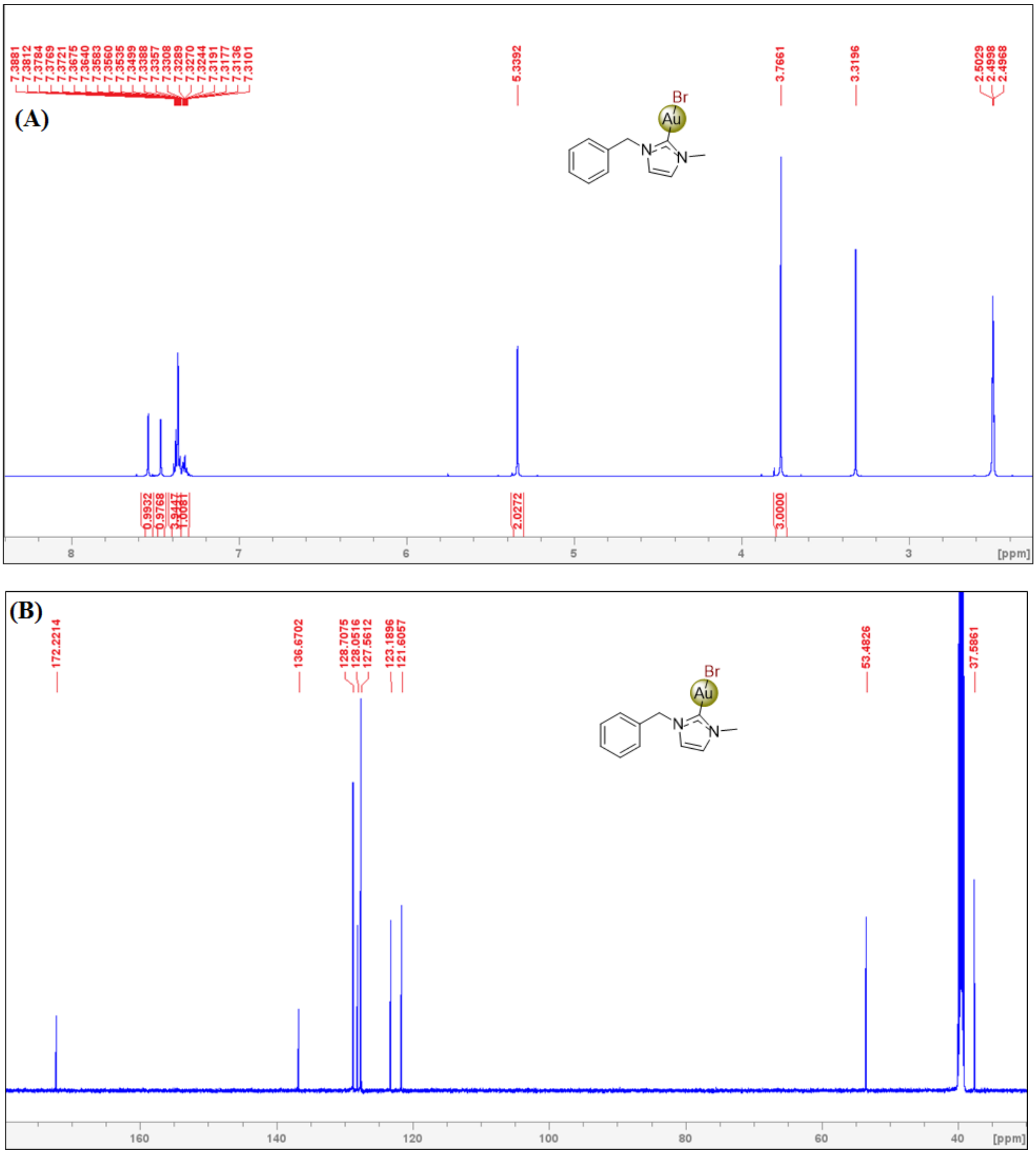

Figure S4. (A) ^{1}H-NMR spectrum and (B) ^{13}C-NMR spectrum of NHC$_1$-Au-Br in DMSO-d6.

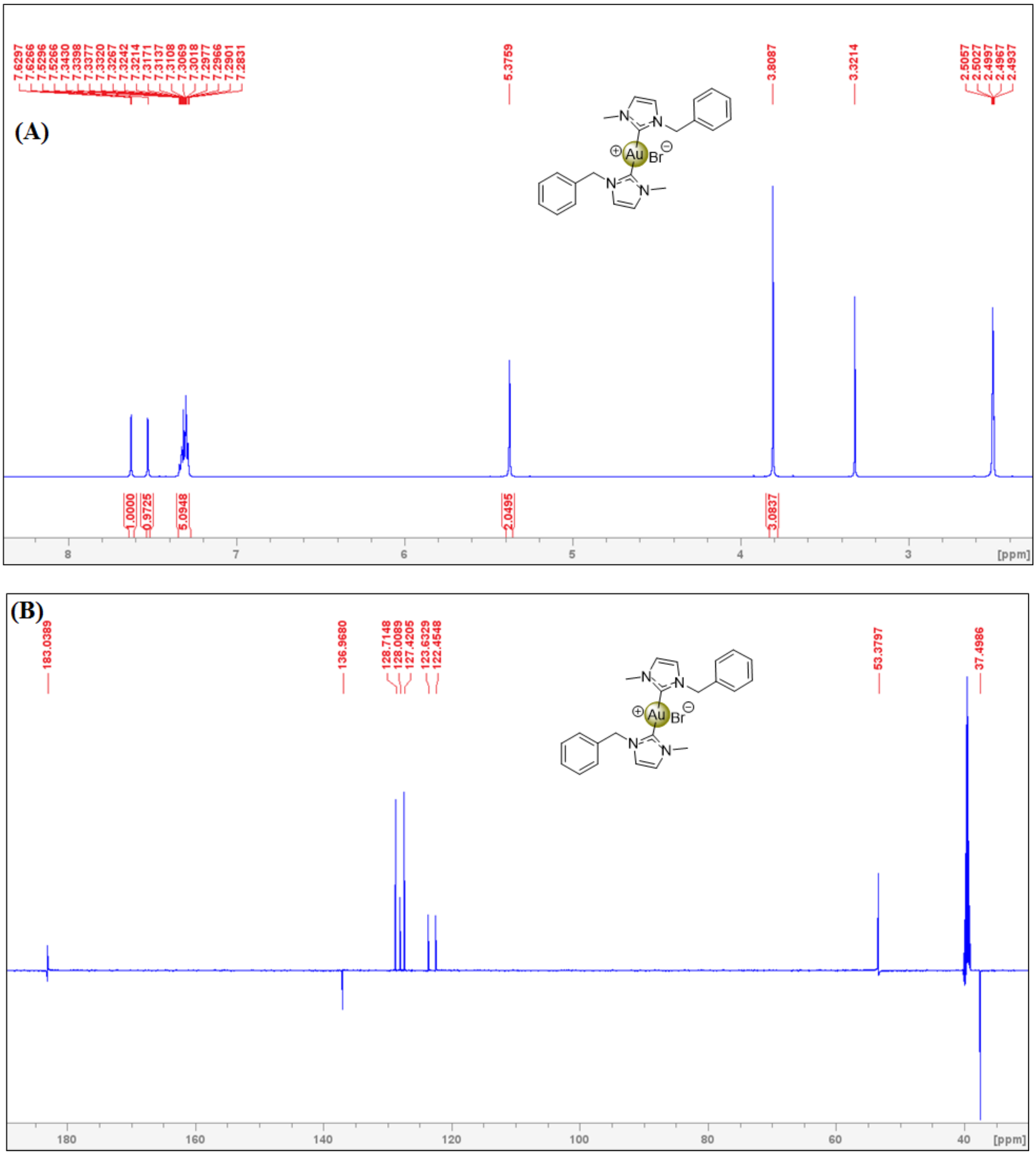

Figure S5. (A) ^{1}H-NMR spectrum and (B) ^{13}C-NMR spectrum of $[(NHC_1)_2Au]Br$ in DMSO-d6.

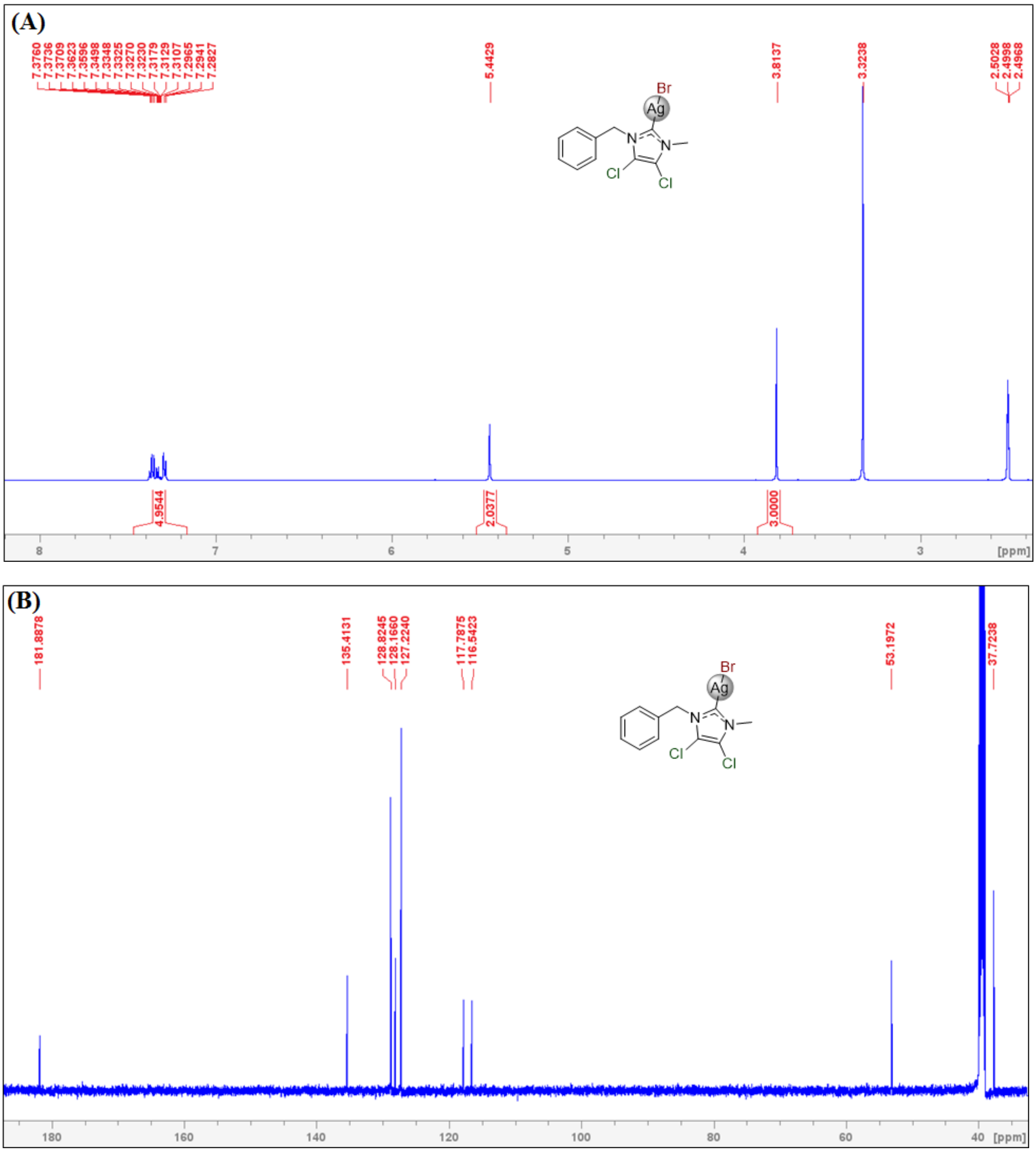

Figure S6. (A) ^{1}H-NMR spectrum and (B) ^{13}C-NMR spectrum of NHC$_2$-Ag-Br in DMSO-d6.

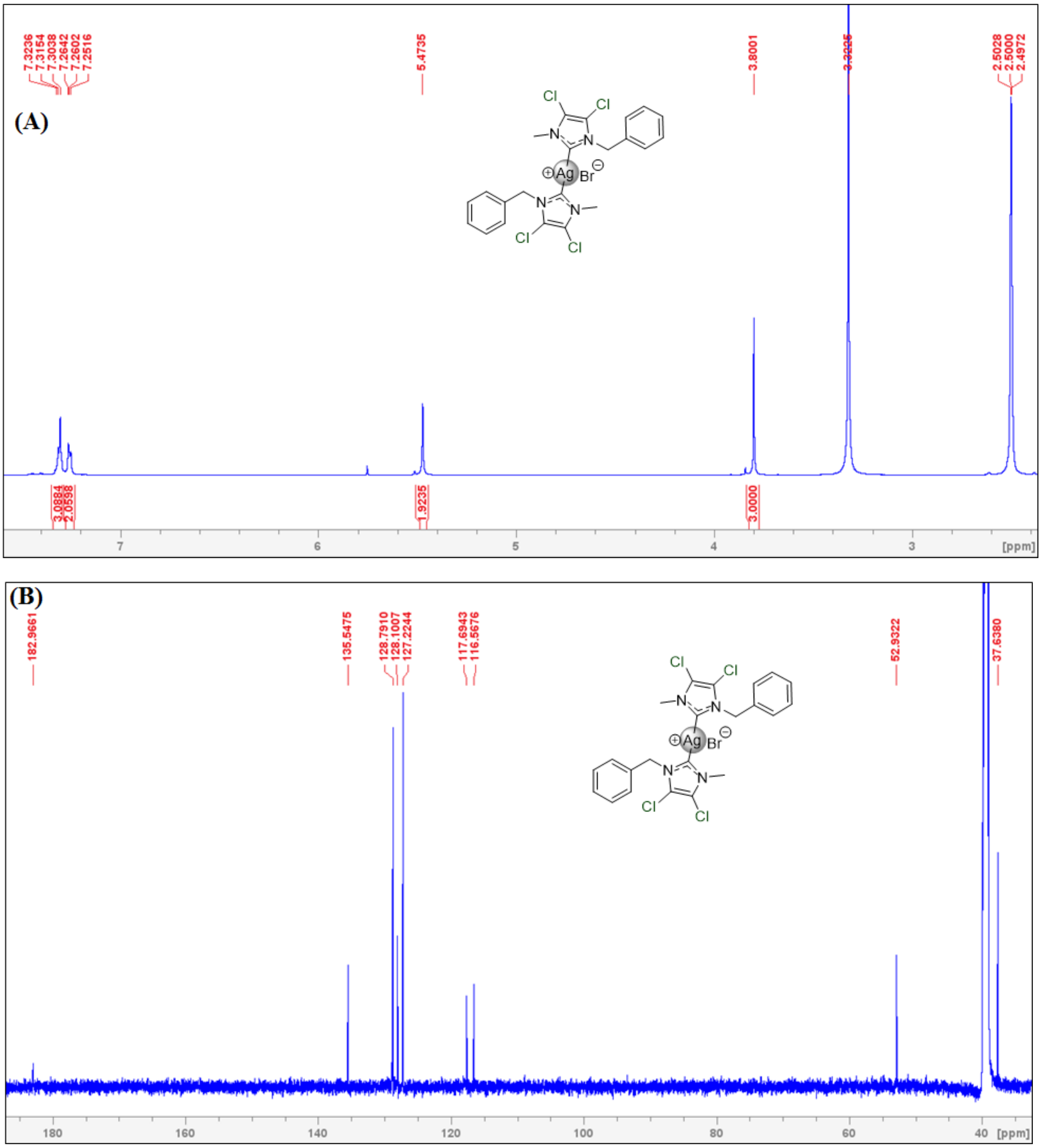

Figure S7. (A) ^{1}H-NMR spectrum and (B) ^{13}C-NMR spectrum of [(NHC$_2$)$_2$Ag]Br in DMSO-d6.

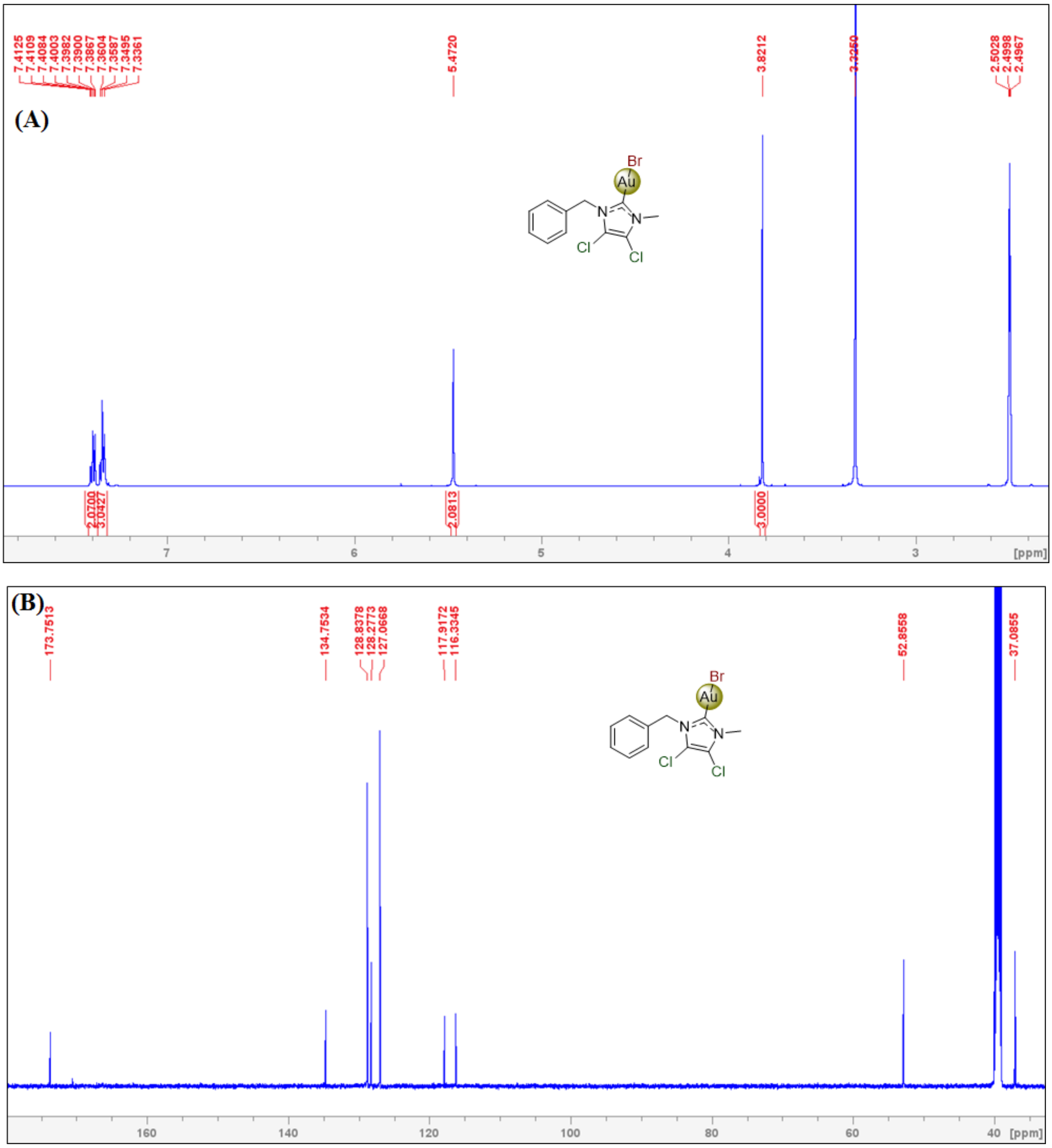

Figure S8. (A) ^{1}H-NMR spectrum and (B) ^{13}C-NMR spectrum of NHC_2-Au-Br in DMSO-d6.

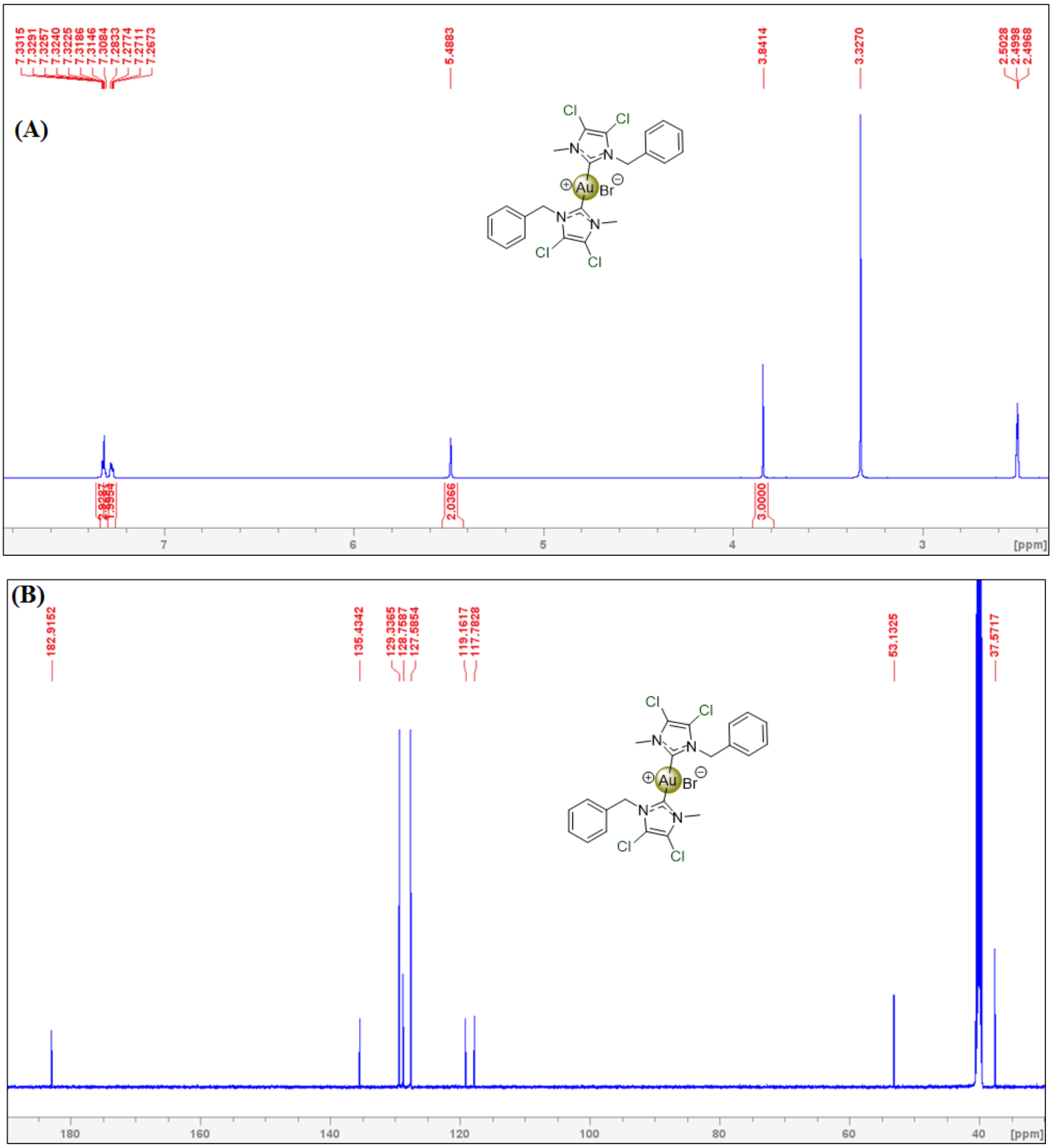

Figure S9. (A) ^{1}H-NMR spectrum and (B) ^{13}C-NMR spectrum of $[(NHC_2)_2Au]Br$ in DMSO-d6.

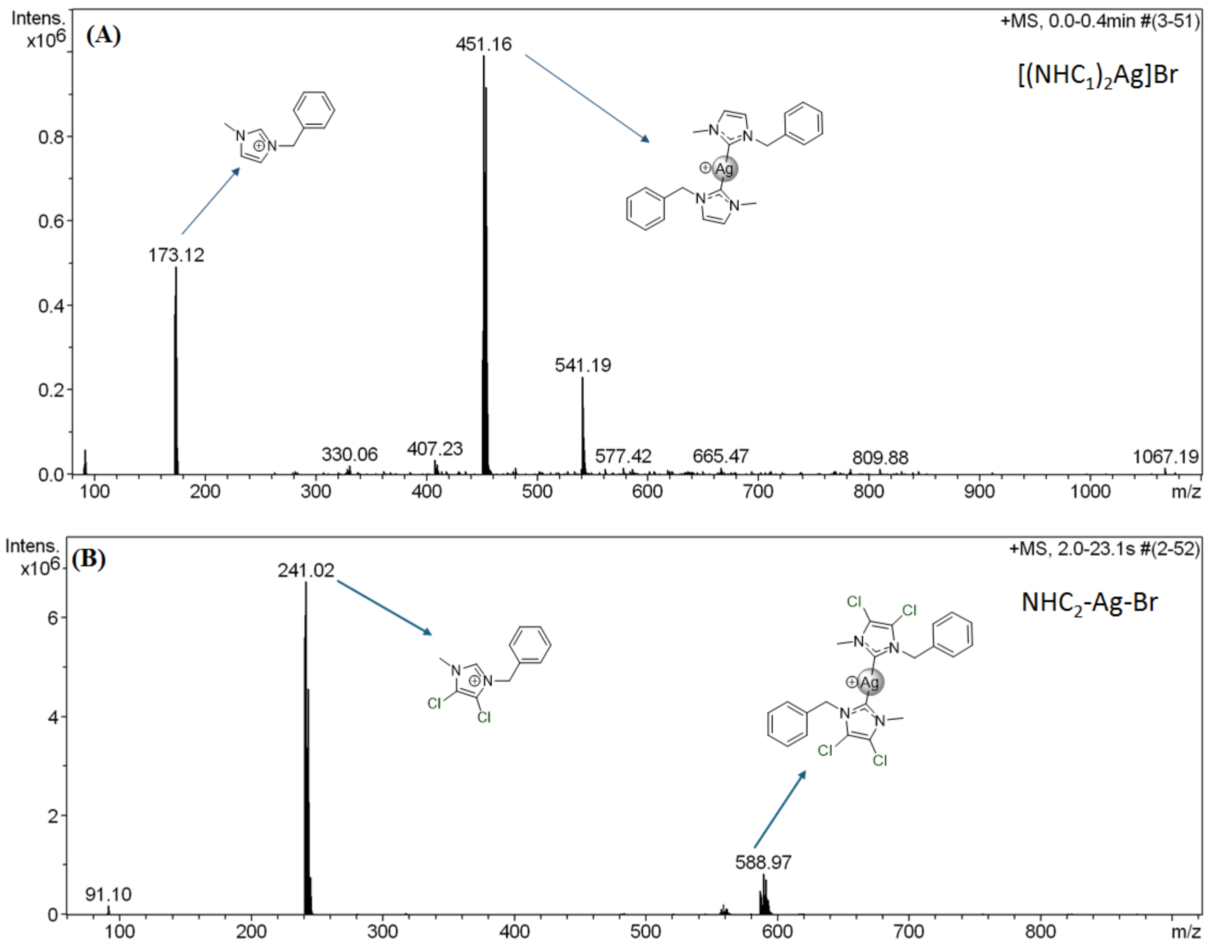

Figure S10. MS spectra of silver-NHC compounds (A) $[(NHC_1)_2Ag]Br$ and (B) NHC_2-Ag-Br.

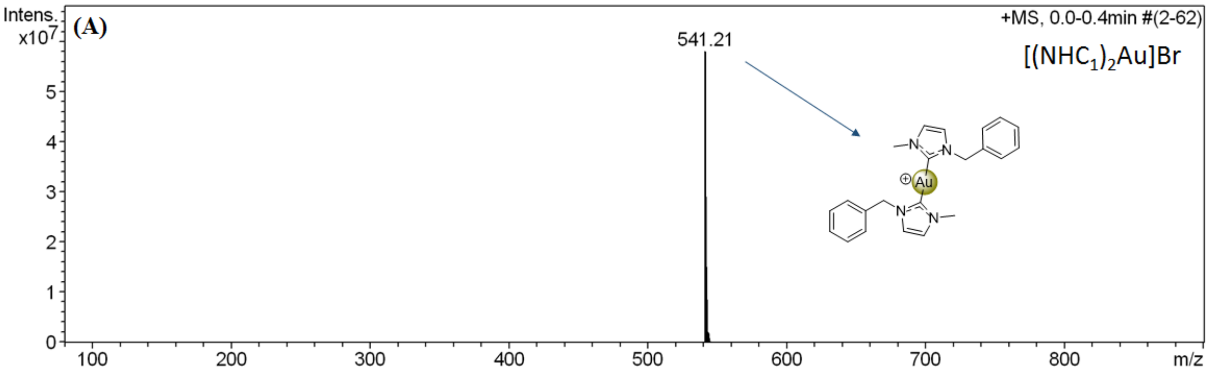

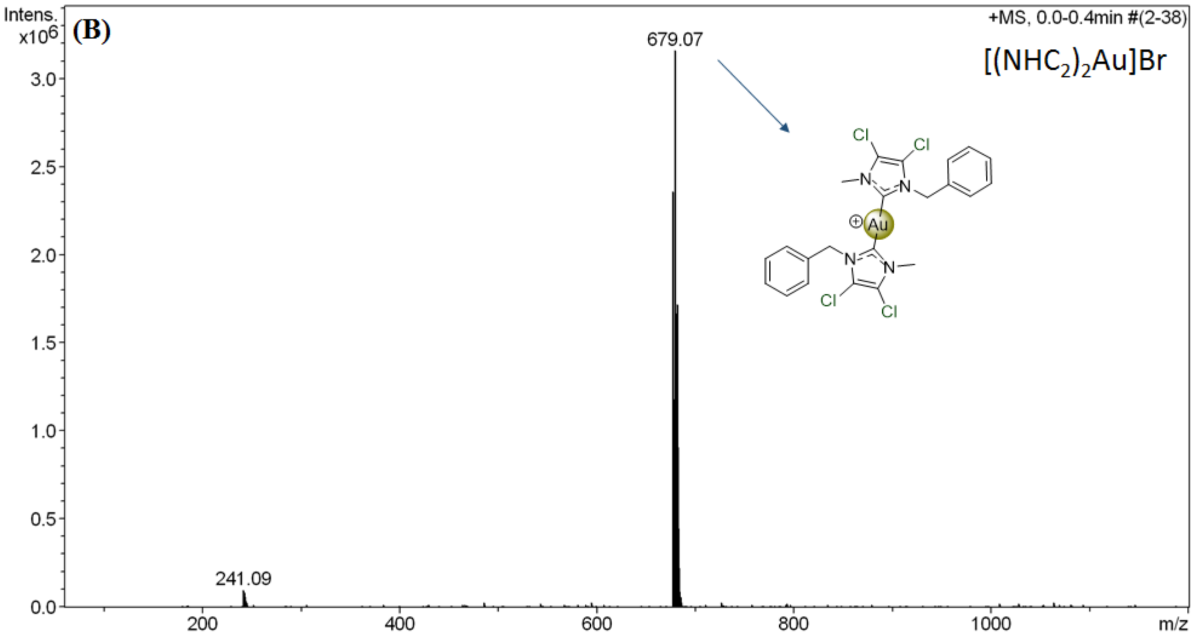

Figure S11. MS spectra of bis-NHC gold compounds (A) $[(NHC_1)_2Au]Br$ and (B) $[(NHC_2)_2Au]Br$.

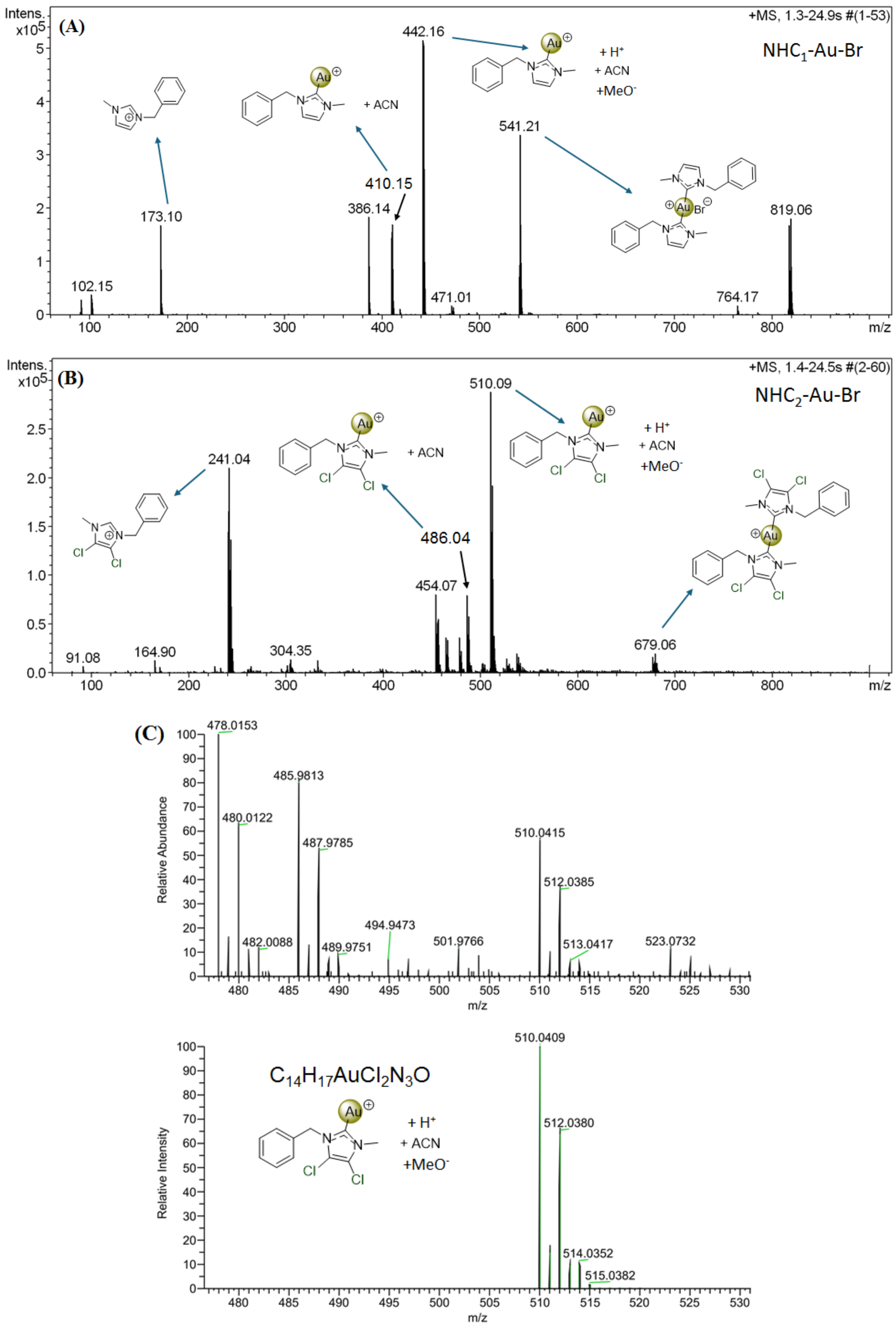

Figure S12. MS spectra of mono-NHC gold compounds. (A) NHC_1-Au-Br, (B) NHC_2-Au-Br, and (C) HRMS of NHC_2-Au-Br, confirming the aduct with ACN and MeO^-.

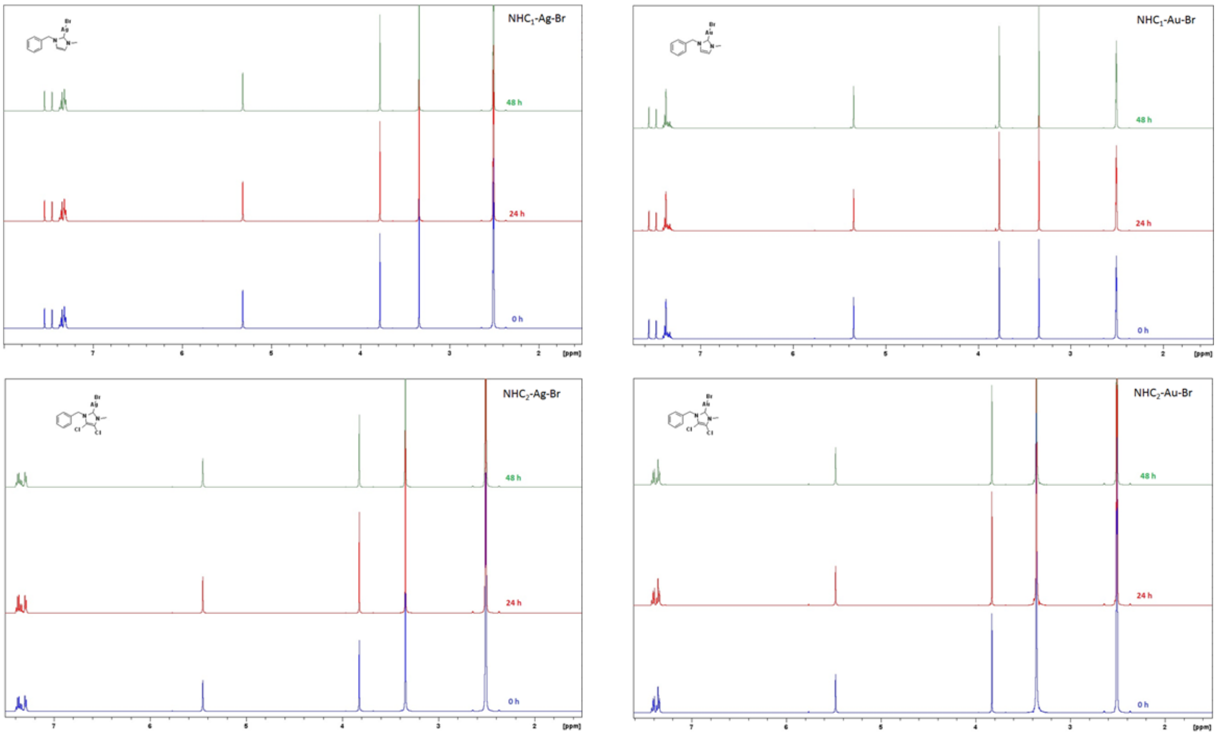

Figure S13. ^{1}H-NMR stability studies of mono-NHC complexes for 48 h in DMSO-d6.

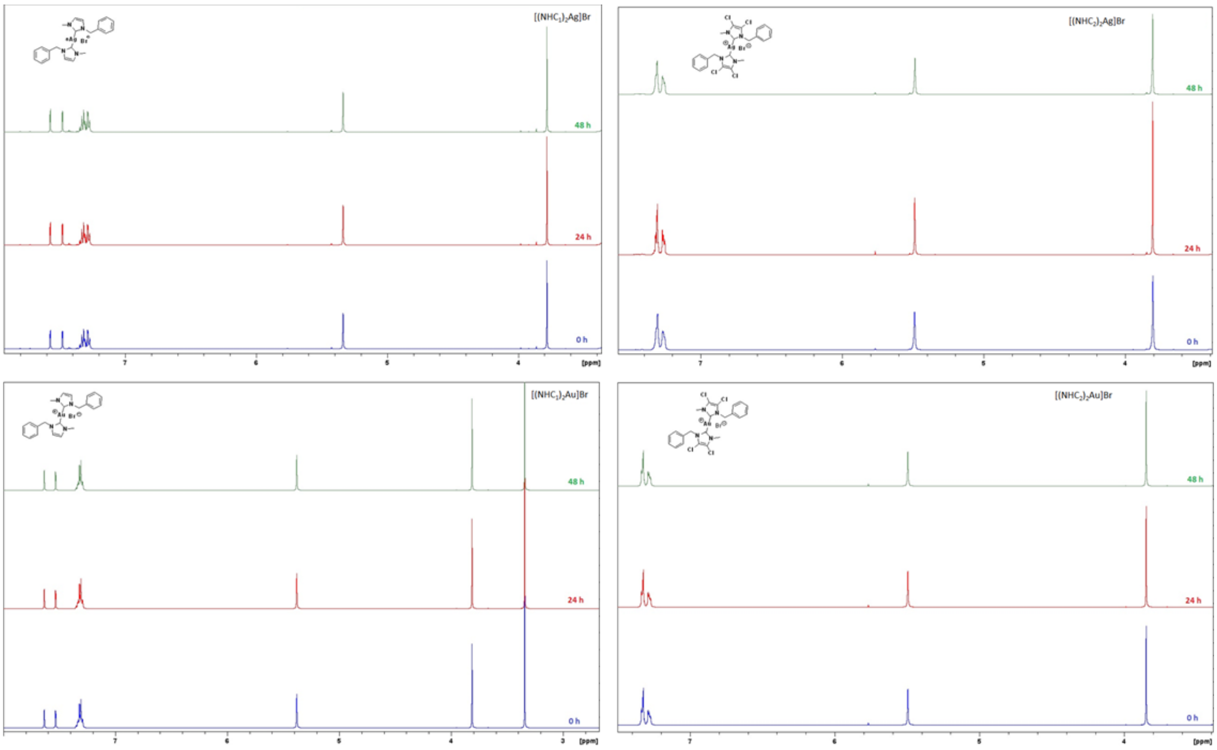

Figure S14. ^{1}H-NMR stability studies of bis-NHC complexes for 48 h in DMSO-d6.

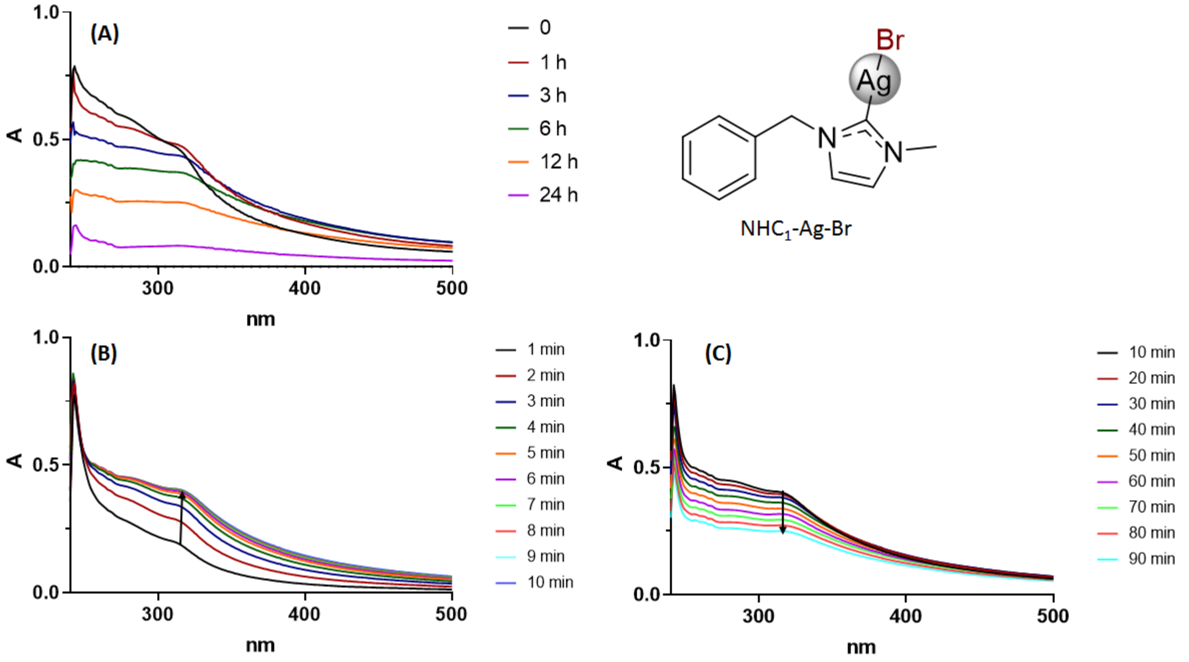

Figure S15. UV-Vis stability measurements of NHC_1-Ag-Br: (A) changes are observed within the first hour, with precipitation over time; (B) within the first 10 min hydrolysis takes place; and (C) after 10 min only precipitation is observed.

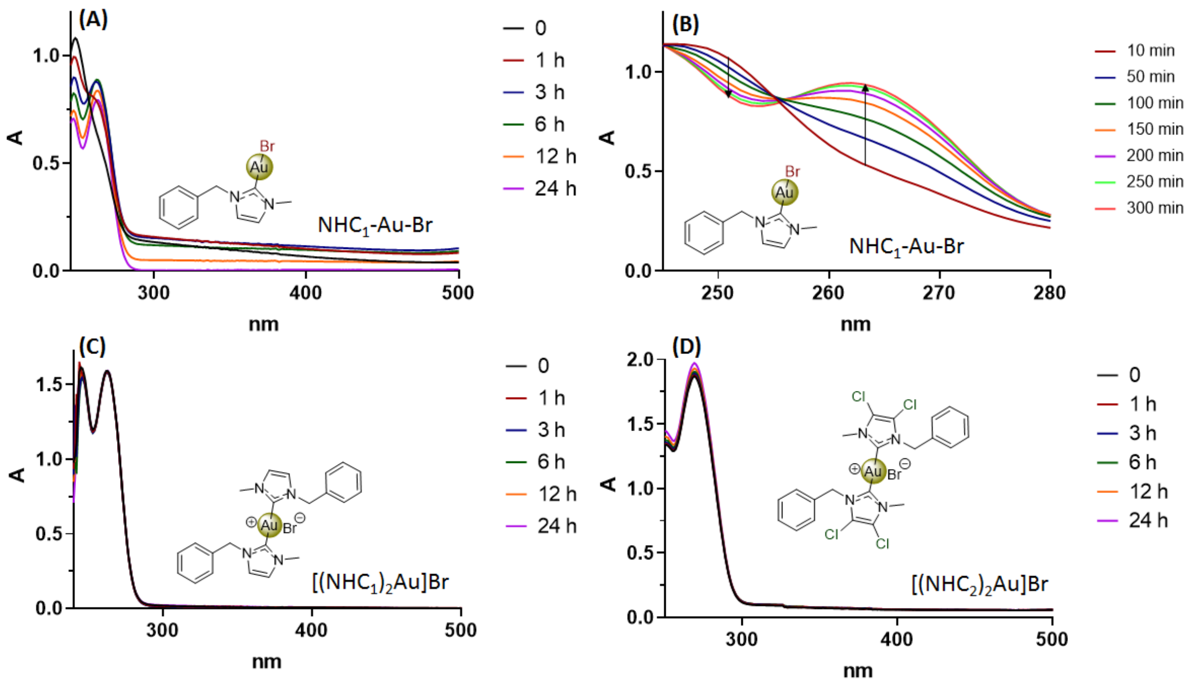

Figure S16. UV-Vis stability measurements of NHC-gold(I) compounds: (A) changes are observed for NHC_1-Au-Br within the first hours, with a slight precipitation over time; (B) within 5 h the mono to bis interchange is observed. The spectra of (C) $[(NHC_1)_2Au]Br$ and (D) $[(NHC_2)_2Au]Br$ do not show any changes within 24 h.

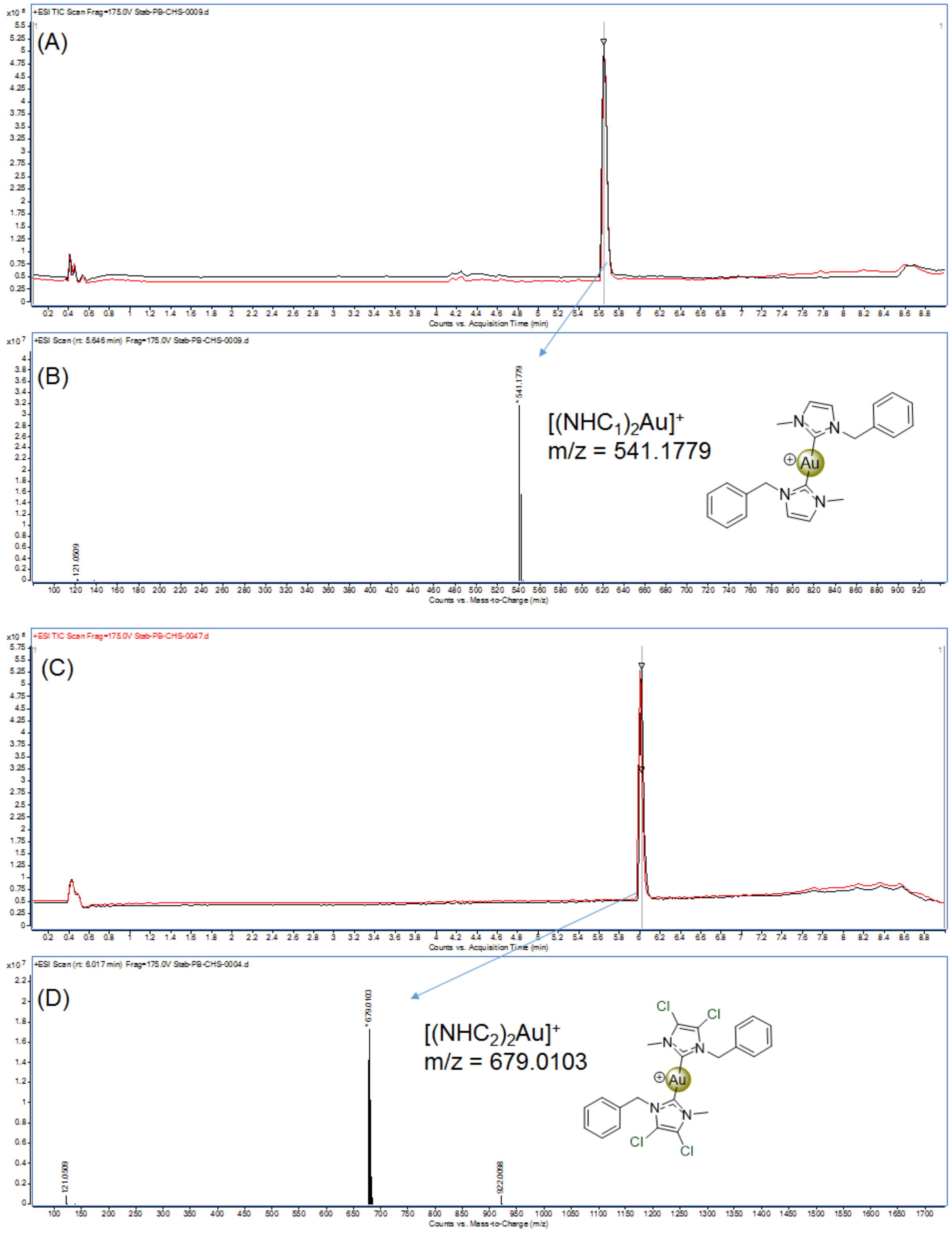

Figure S17. HPLC-MS measurement of (A+B) $[(NHC_1)_2Au]Br$ and (C+D) $[(NHC_2)_2Au]Br$ at 0 h (black) and after 24 h (red) in PB (10 µM, < 1 % DMSO). No visible formation of new peaks was observed, confirming the compound structures in PB solution. (A+C) are total ion chromatograms. Two internal standards are visible in the MS spectra [121.05 and 922.01 m/z].

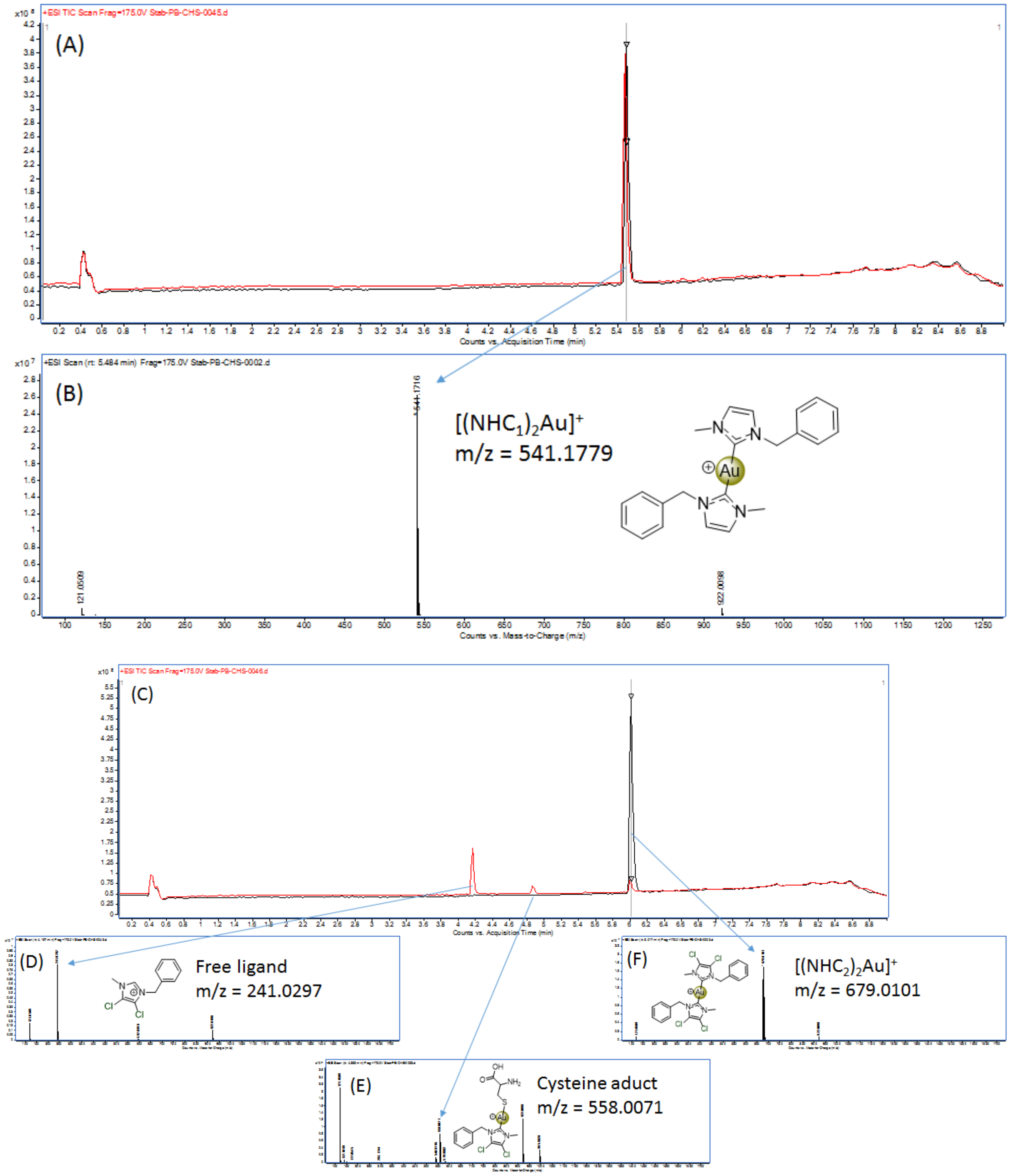

Figure S18. HPLC-MS measurement of (A+B) $[(NHC_1)_2Au]Br$ and (C+D+E+F) $[(NHC_2)_2Au]Br$ at 0 h (black) and after 24 h (red) with 5 eq. of L-cysteine (10 µM of gold compound, < 1 % DMSO). Only for $[(NHC_2)_2Au]Br$ the formation of (D) the free ligand and (E) the cysteine-adduct (NHC_2-Au-Cys) could be observed. (A+C) are total ion chromatograms. Two internal standards are visible in the MS spectra [121.05 and 922.01 m/z].

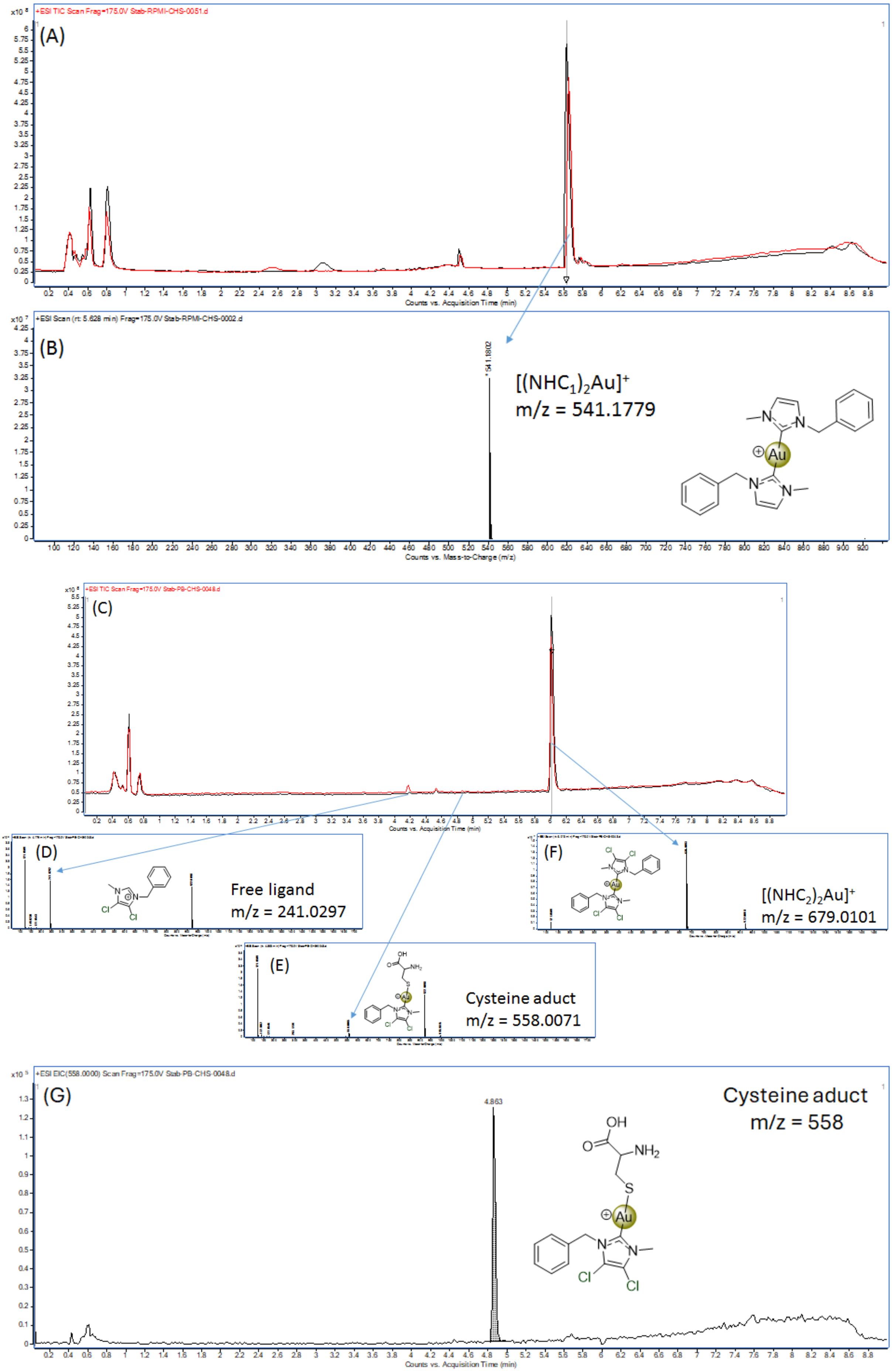
+ESI TIC Scan Frag=175.0V Stab-RPMI-CHS-0051.d
(A)
Counts vs. Acquisition Time (min)
+ESI Scan (rt: 5.628 min) Frag=175.0V Stab-RPMI-CHS-0002.d
(B)
541.1802
$[(NHC_1)_2Au]^+$
m/z = 541.1779
Counts vs. Mass-to-Charge (m/z)
+ESI TIC Scan Frag=175.0V Stab-PB-CHS-0048.d
(C)
Counts vs. Acquisition Time (min)
(D)
Free ligand
m/z = 241.0297
(F)
$[(NHC_2)_2Au]^+$
m/z = 679.0101
(E)
Cysteine aduct
m/z = 558.0071
+ESI EIC(558.0000) Scan Frag=175.0V Stab-PB-CHS-0048.d
(G)
4.863
Cysteine aduct
m/z = 558
Counts vs. Acquisition Time (min)

Figure S19. HPLC-MS measurement of (A+B) $[(NHC_1)_2Au]Br$ and (C+D+E+F) $[(NHC_2)_2Au]Br$ at 0 h (black) and after 24 h (red) in RPMI (10 µM, < 1 % DMSO). Only for $[(NHC_2)_2Au]Br$ the formation of (D) the free ligand and (E) the cysteine-adduct (NHC_2-Au-Cys) could be observed. (A+C) are total ion chromatograms, while (G) is the extracted ion chromatogram (EIC) for NHC_2-Au-Cys ([558.0 ± 0.5] m/z). Two internal standards are visible in the MS spectra [121.05 and 922.01 m/z].

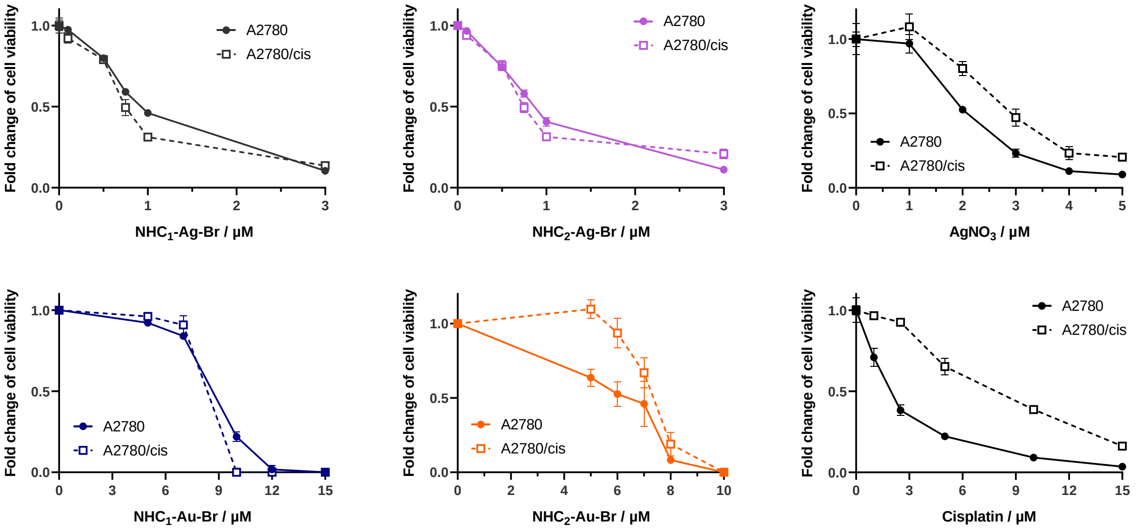

Figure S20. Resistance of A2780/cis cells against mono-NHC silver and gold complexes, Auranofin, and Cisplatin. Cell viability was evaluated by the MTT viability assay after 72 h drug incubation in A2780 vs A2780/cis cells. Concentration-response curves and mean ± standard deviation (SD) were calculated from triplicates of one representative experiment out of three.

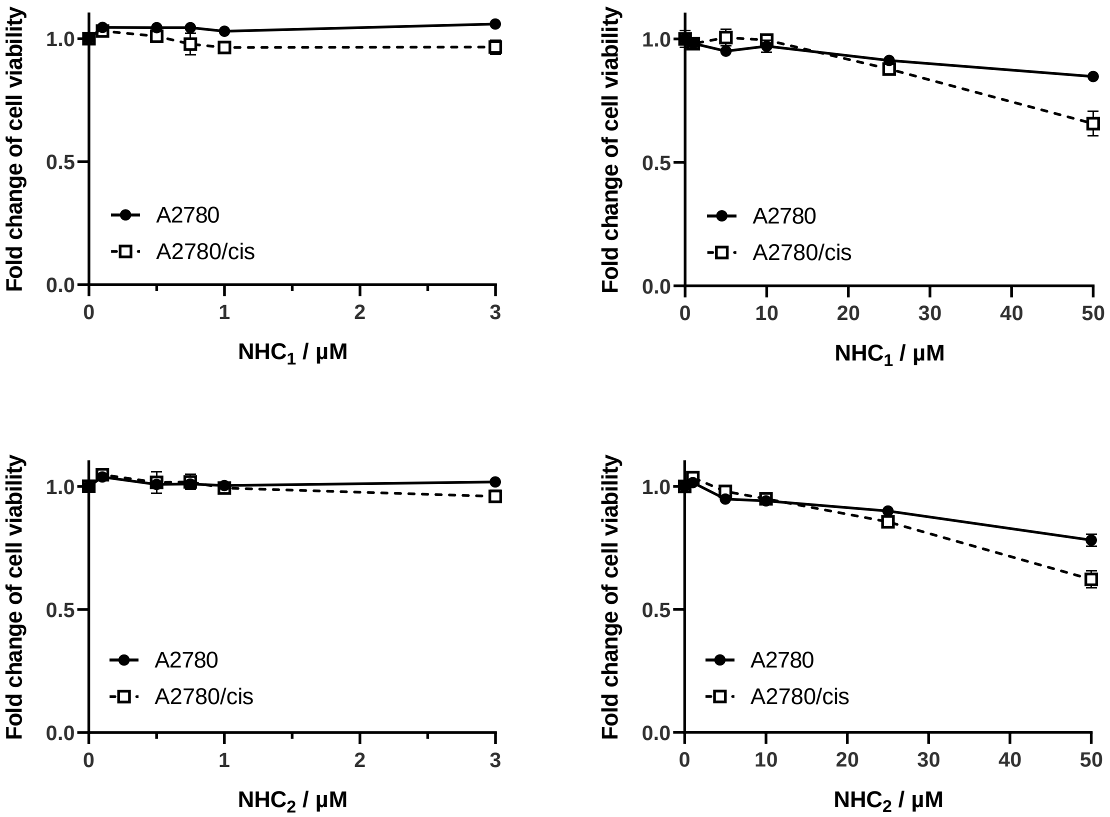

Figure S21. Anticancer activity of NHC_1 and NHC_2. Cell viability was evaluated by the MTT viability assay after 72 h drug incubation in A2780 vs A2780/cis cells. Concentration-response curves and mean ± standard deviation (SD) were calculated from triplicates of one representative experiment.

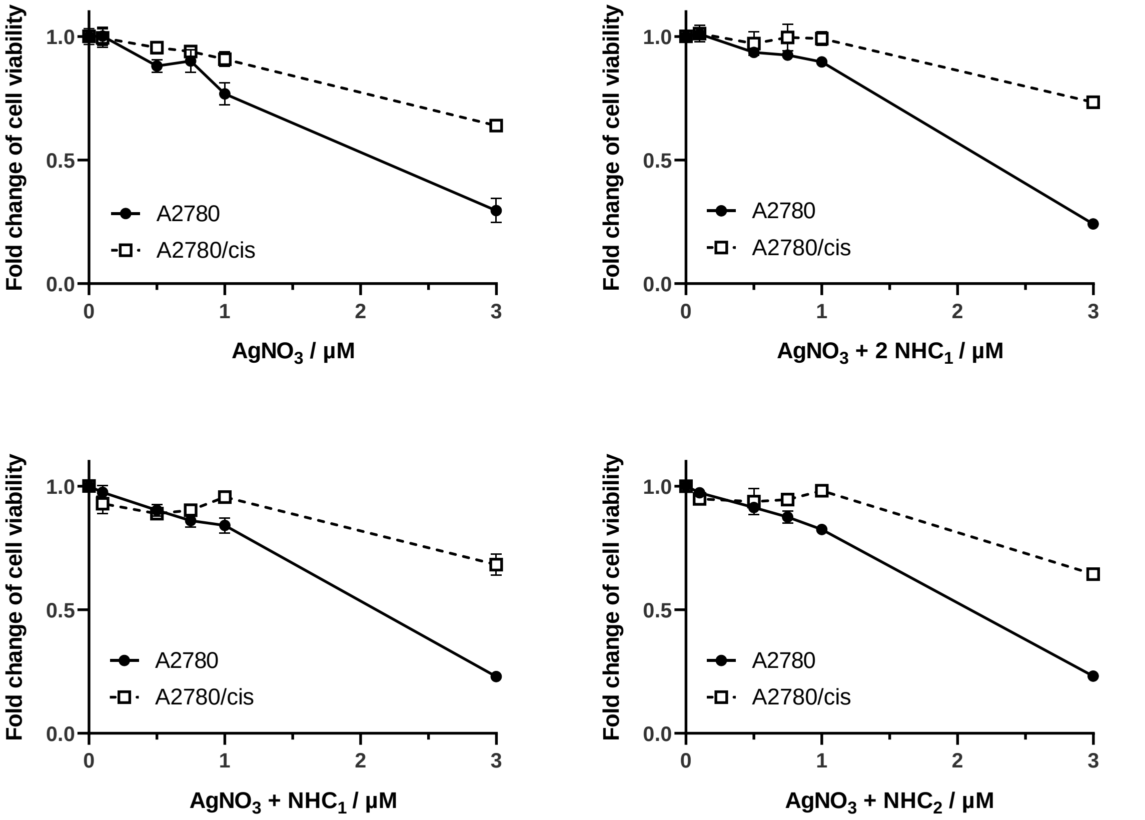

Figure S22. Anticancer activity of $AgNO_3$ alone and in combination with the free ligands. Cell viability was evaluated by the MTT viability assay after 72 h drug incubation in A2780 vs A2780/cis cells. Concentration-response curves and mean ± standard deviation (SD) were calculated from triplicates of one representative experiment.

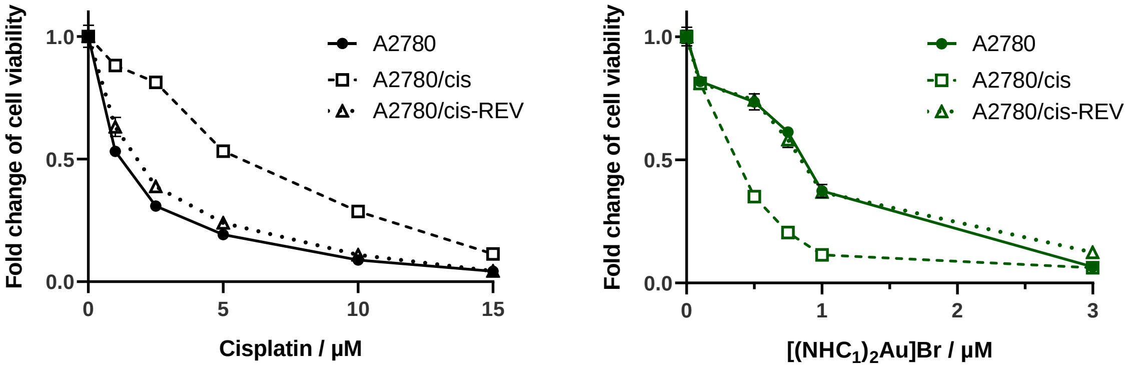

Figure S23. Activity of Cisplatin and $[(NHC_1)_2Au]Br$ in A2780/cis-REV (revertant) cells. Cell viability was evaluated by the MTT viability assay after 72 h drug incubation in A2780, A2780/cis and A2780/cis-REV cells. Concentration-response curves and mean ± standard deviation (SD) were calculated from triplicates of one representative experiment.

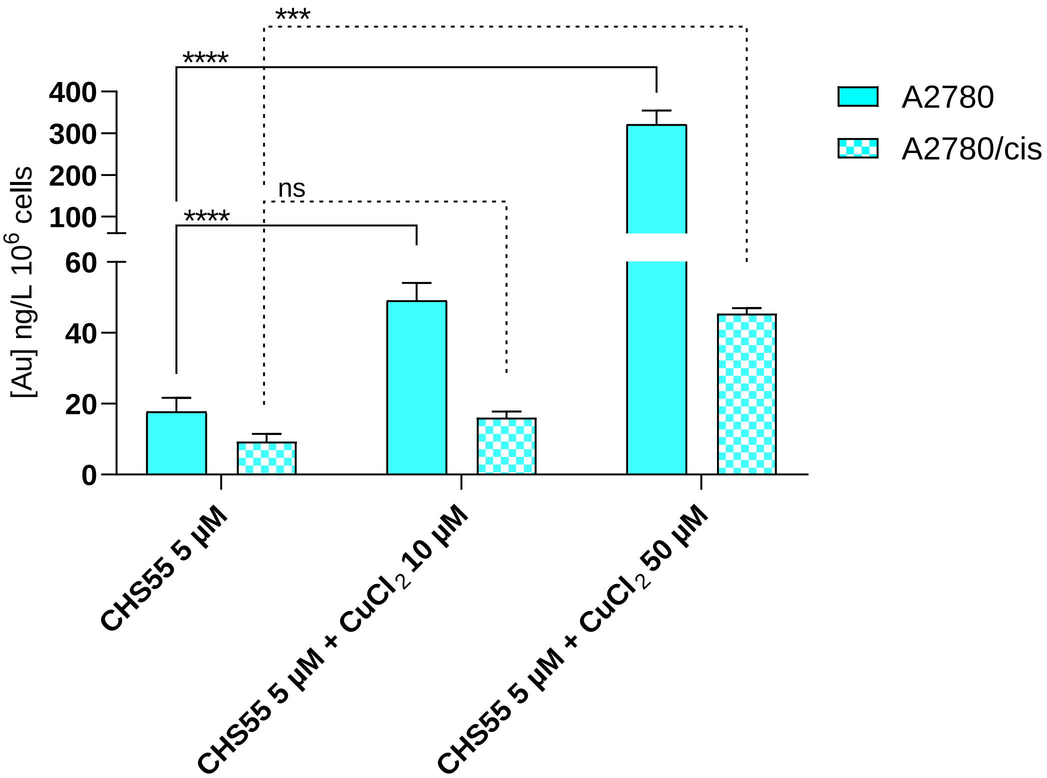

Figure S24. Intracellular gold levels after treatment with $[(NHC_2)_2Au]Br$ at 5 µM alone or in combination with 10 µM or 50 µM of $CuCl_2$ in A2780 and A2780/cis cells after 5 h incubation at 37 °C. Cells were digested and measured by ICP-MS. Results were normalized to the cell number, and values are given as mean ± SD of two experiments. Statistical significance was calculated using two-way ANOVA (***p < 0.001; ****p < 0.0001; ns: not significant).

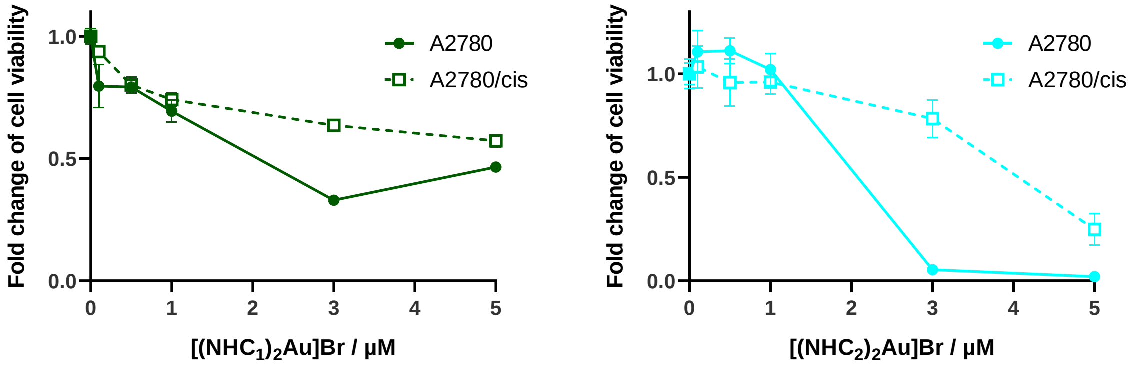

Figure S25. Viability assays of A2780 and A2780/cis cells after 24 h treatment with $[(NHC_1)_2Au]Br$ and $[(NHC_2)_2Au]Br$. Anticancer activity was evaluated by the MTT viability assay after 24 h drug incubation in A2780 vs A2780/cis cells. Concentration-response curves and mean ± standard deviation (SD) were calculated from triplicates of one representative experiment.

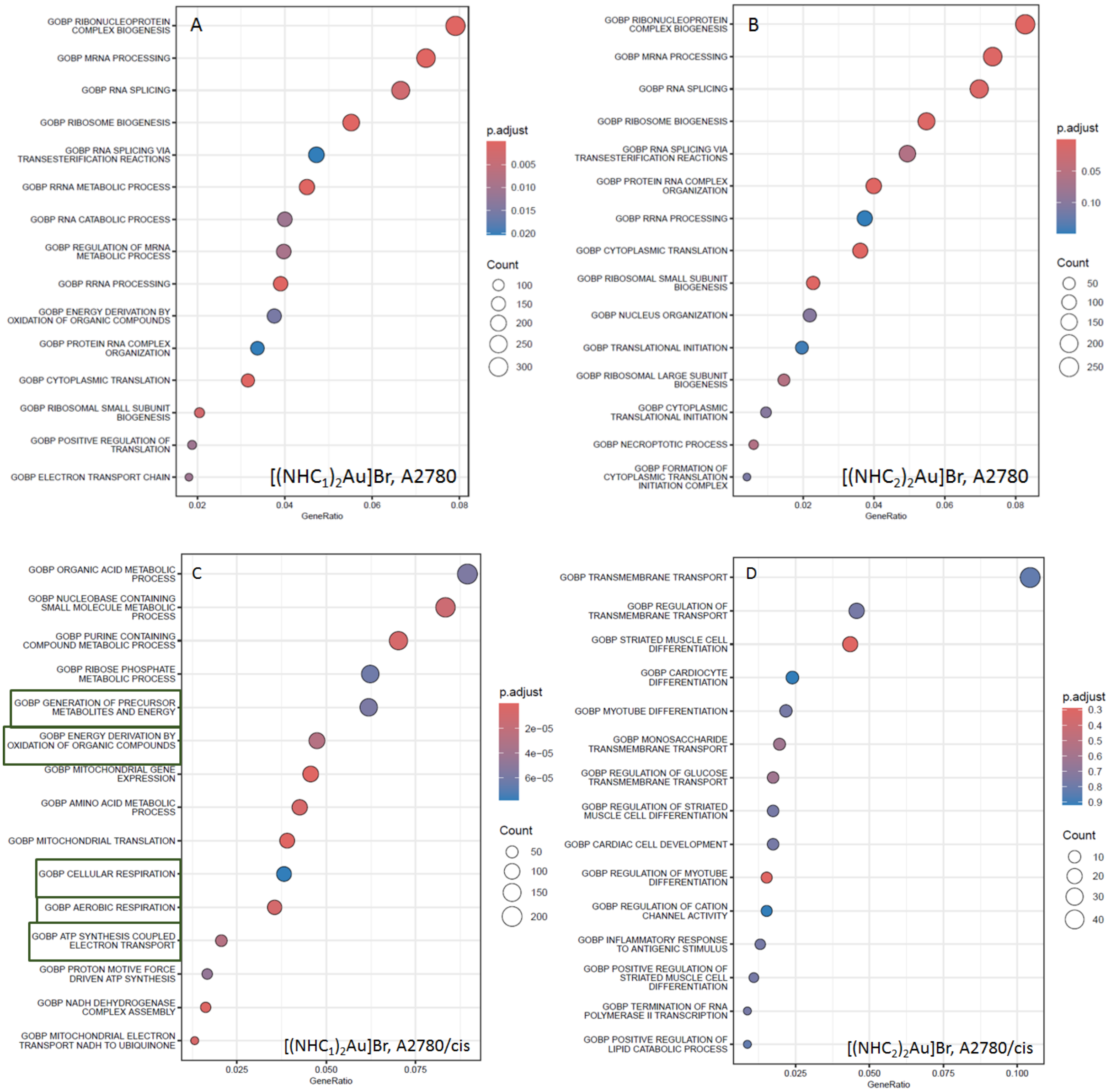

Figure S26. Top 15 GOBP terms from (A+B) A2780 or (C+D) A2780/cis cells treated with (A+C) $[(NHC_1)_2Au]Br$ or (B+D) $[(NHC_2)_2Au]Br$ compared to the solvent-treated group. Cells were treated with 1 µM of either bis-NHC gold drug for 16 h, followed by shotgun proteomics.

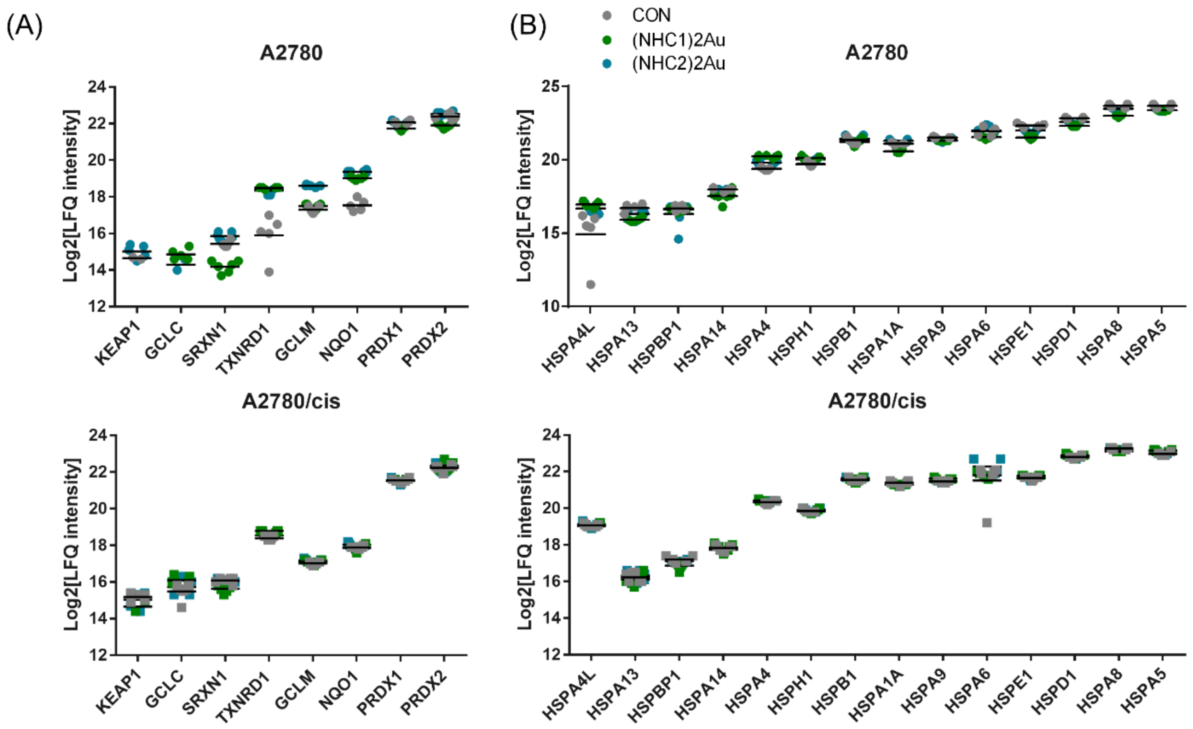

Figure S27. Dot plots of proteins related to the NRF2-KEAP1 (A) and heat shock (B) stress responses. Both A2780 and A2780/cis cells were treated with vehicle control (CON), $[(NHC_1)_2Au]Br$, or $[(NHC_2)_2Au]Br$ in hexuplicates.

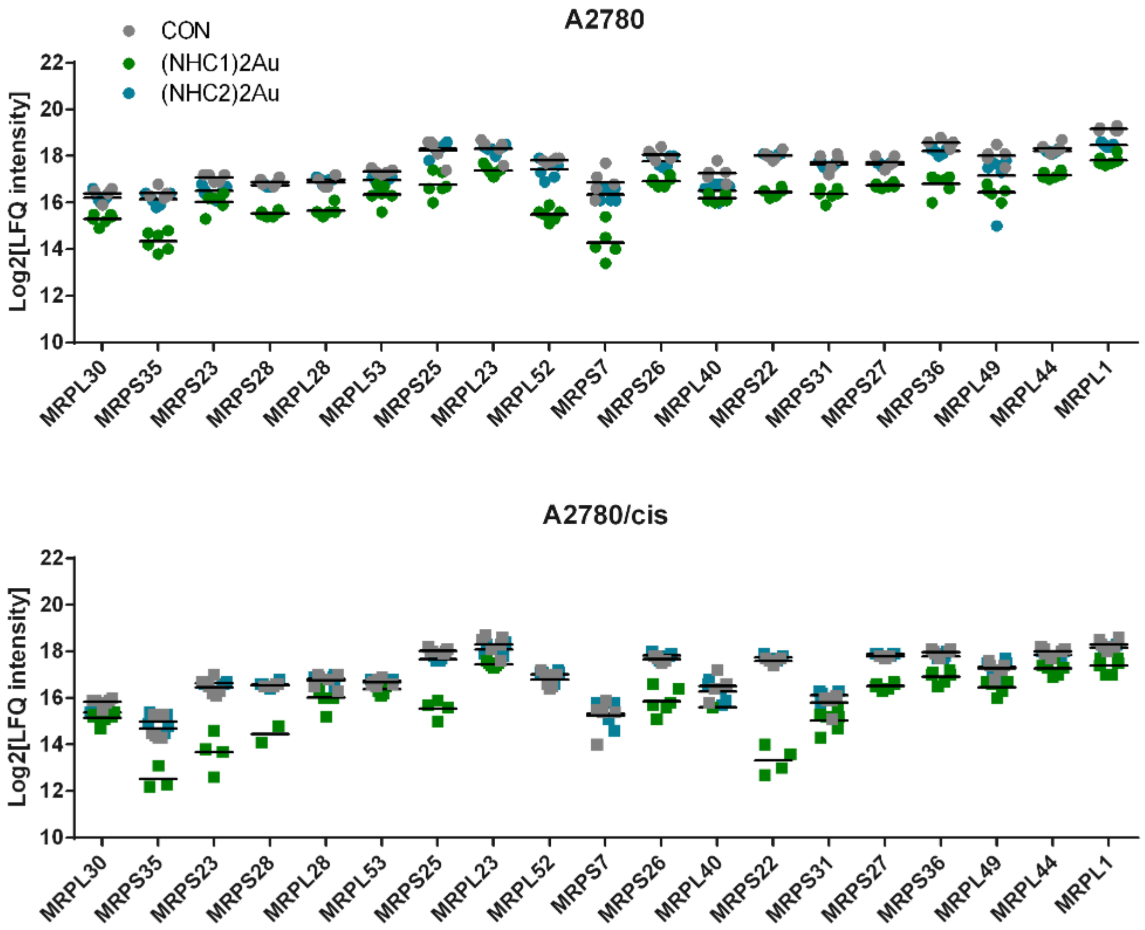

Figure S28. Dot plots of proteins related to the mitochondrial translation. Both A2780 and A2780/cis cells were treated with vehicle control (CON), [$(NHC_1)_2$Au]Br, or [$(NHC_2)_2$Au]Br in hexuplicates.

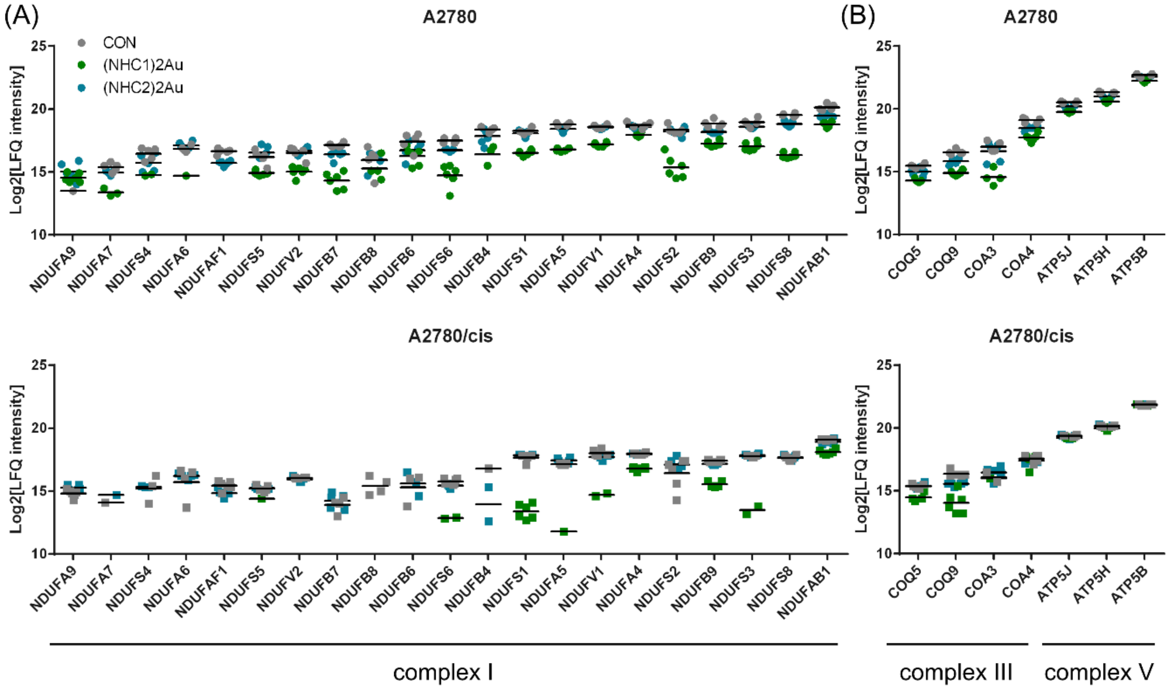

Figure S29. Dot plots of proteins related to oxidative phosphorylation, especially complex I, complex III and complex V. Both A2780 and A2780/cis cells were treated with vehicle control (CON), $[(NHC_1)_2Au]Br$, or $[(NHC_2)_2Au]Br$ in hexuplicates.

Table S1. Gene ontology biological processes (GOBP) that are either upregulated in A2780/cis or A2780 are shown, including the number of proteins per term and adjusted p-value according to Benjamini-Hochberg. Proteins were considered that had a fold-change ≥2 in either direction.

**GOBP (A2780/cis up)**	**N° proteins**	**Adj. P-value**
rRNA processing	40	4.62E-14
Vesicle-mediated transport	50	2.37E-11
Mitochondrial translation	23	3.28E-6
Translation	37	4.89E-6
Glutamate metabolic process	8	3.97E-3
Pentose-phosphate shunt	7	3.99E-3
Fructose 6-phosphate metabolic process	7	9.98E3
**GOBP (A2780 up)**	**N° proteins**	**Adj. P-value**
Oxidative phosphorylation	28	5.03E-10
Chemical carcinogenesis - reactive oxygen species	30	1.04E-6
Focal adhesion	18	4.45E-2

Table S2. Number of identified proteins in each perturbation of $[(NHC_1)_2Au]Br$- and $[(NHC_2)_2Au]Br$-treated parental A2780 and resistant A2780/cis cells. Cells were treated at 1 µM for 16 h and whole cell lysates were collected for proteome profiling. Regulated proteins were identified by statistical significance using multiple-testing correction (FDR 0.05, S0 = 0.1).

Cell Line	Treatment	N° identified proteins	N° regulated proteins	Percentage of regulated proteins
A2780	$[(NHC_1)_2Au]Br$	3975	2849	72%
A2780	$[(NHC_2)_2Au]Br$	3948	2097	53%
A2780/cis	$[(NHC_1)_2Au]Br$	3957	983	47%
A2780/cis	$[(NHC_2)_2Au]Br$	4210	107	11%

Table S3. ICP-MS equipment parameters for the measurement of silver and gold.

RF power	1550 W
Nebulizer	MicroMist
Spray chamber	Scott double-pass
Monitored isotopes	^{115}In, ^{107}Ag for silver; ^{185}Re, ^{197}Au for gold
Measurement modes	standard mode
Plasma gas	15 L min^{-1}
Nebulizer gas	1.08 L min^{-1}
Auxiliary gas	0.90 L min^{-1}
Cones	Ni
Integration time	0.1
Number of replicates	6
Number of sweeps	100
